# Bioenergetic characterization of a shallow-sea hydrothermal vent system: Milos Island, Greece

**DOI:** 10.1371/journal.pone.0234175

**Published:** 2020-06-05

**Authors:** Guang-Sin Lu, Douglas E. LaRowe, David A. Fike, Gregory K. Druschel, William P. Gilhooly, Roy E. Price, Jan P. Amend

**Affiliations:** 1 Department of Earth Sciences, University of Southern California, Los Angeles, California, United States of America; 2 Department of Earth and Planetary Sciences, Washington University in St. Louis, St. Louis, Missouri, United States of America; 3 Department of Earth Sciences, Indiana University Purdue University Indianapolis, Indianapolis, Indiana, United States of America; 4 School of Marine and Atmospheric Sciences, The State University of New York, Stony Brook, New York, United States of America; 5 Department of Biological Sciences, University of Southern California, Los Angeles, California, United States of America; The University of Akron, UNITED STATES

## Abstract

Shallow-sea hydrothermal systems, like their deep-sea and terrestrial counterparts, can serve as relatively accessible portals into the microbial ecology of subsurface environments. In this study, we determined the chemical composition of 47 sediment porewater samples along a transect from a diffuse shallow-sea hydrothermal vent to a non-thermal background area in Paleochori Bay, Milos Island, Greece. These geochemical data were combined with thermodynamic calculations to quantify potential sources of energy that may support *in situ* chemolithotrophy. The Gibbs energies (Δ*G*_*r*_) of 730 redox reactions involving 23 inorganic H-, O-, C-, N-, S-, Fe-, Mn-, and As-bearing compounds were calculated. Of these reactions, 379 were exergonic at one or more sampling locations. The greatest energy yields were from anaerobic CO oxidation with NO_2_^-^ (-136 to -162 kJ/mol e^-^), followed by reactions in which the electron acceptor/donor pairs were O_2_/CO, NO_3_^-^/CO, and NO_2_^-^/H_2_S. When expressed as energy densities (where the concentration of the limiting reactant is taken into account), a different set of redox reactions are the most exergonic: in sediments affected by hydrothermal input, sulfide oxidation with a range of electron acceptors or nitrite reduction with different electron donors provide 85~245 J per kg of sediment, whereas in sediments less affected or unaffected by hydrothermal input, various S^0^ oxidation reactions and aerobic respiration reactions with several different electron donors are most energy-yielding (80~95 J per kg of sediment). A model that considers seawater mixing with hydrothermal fluids revealed that there is up to ~50 times more energy available for microorganisms that can use S^0^ or H_2_S as electron donors and NO_2_^-^ or O_2_ as electron acceptors compared to other reactions. In addition to revealing likely metabolic pathways in the near-surface and subsurface mixing zones, thermodynamic calculations like these can help guide novel microbial cultivation efforts to isolate new species.

## Introduction

Hydrothermal systems are prevalent in tectonically active settings, including plate boundaries and hot spots [1–5]. They are commonly categorized by location and water depth into (1) terrestrial, (2) deep-sea (water depth >200 m), and (3) shallow-sea (water depth <200 m) [4, 6, 7]. Due to their accessibility, terrestrial hydrothermal systems (often synonymous with geothermal springs) were the first to be explored [1, 3, 7]. Since the discovery in 1977 of the first deep-sea hydrothermal systems near the Galápagos Islands [8], ~700 hydrothermal vents (with 644 confirmed or inferred to be active) have been reported along the ~60,000 km-long ocean ridge system as well as in back-arc basins in every ocean basin (InterRidge Vents Database: https://vents-data.interridge.org/). Approximately 70 active shallow-sea hydrothermal vent systems have also been identified [6, 9, 10]. Compared to their deep-sea counterparts, they occur in more diverse tectonically active settings, including near submarine volcanoes, island and intra-oceanic arcs, ridge environments, intraplate oceanic volcanoes, continental margins, and rift basins. Corresponding to their setting, the source of water for these systems can be a mixture of meteoric, magmatic, groundwater, and/or seawater. Unlike deep-sea systems, their location in the euphotic zone allows for photosynthetic activity as well [6, 9, 10]. Perhaps because they are influenced by and transitional between terrestrial and off-shore geologic environments, shallow-sea hydrothermal vent systems are typically complex and dynamic, establishing unique microbial ecosystems.

The microbial ecology and physiology in and around hydrothermal systems—terrestrial and marine—have been studied for several decades, but the factors that control community composition and metabolic function remain elusive. What is known, however, is that these systems contain the necessary ingredients for life—carbon sources, chemical energy from thermodynamic disequilibrium, mineral surfaces, and compositional gradients. Because all biological processes, including anabolism, catabolism, growth, development, and reproduction, are dependent on energy transformations [11–17], quantifying the amounts of energy associated with biological processes guides our understanding of ecosystem dynamics. The amount of energy that microorganisms can gain by catalyzing catabolic reactions in their environment can be quantified by calculating the Gibbs energy of redox reactions (Δ*G*_*r*_), which depends on physicochemical variables, including temperature, pressure, pH, concentrations of products and reactants, and ionic strength. These physicochemical variables and consequently the redox reaction energy yields can vary considerably from one hydrothermal system to the next, with the structure and function of the resident microbial community closely tied to the geologic setting. We can build upon earlier studies that have shown that thermophilic archaea and bacteria in these environments can catalyze a tremendous array of redox reactions to gain energy. Many of these thermophiles are chemolithoautotrophic, i.e., they use metabolic strategies that rely only on inorganic compounds as sources of energy and carbon [14, 18–22]. In fact, a number of studies have quantified the energetic potentials in terrestrial geothermal springs [23–29], deep-sea hydrothermal systems [30–35], and shallow-sea hydrothermal systems [22, 36–42].

Several studies have described the geology, geochemistry and microbiology of the shallow-sea hydrothermal system at Milos [43–49], but the bioenergetic potential there has not been quantified. In this study, we quantify the energetics of 730 inorganic redox reactions in a shallow-sea hydrothermal system of Milos Island, Greece. The reactions include electron donors and acceptors of five major elements (H, C, N, O, S) and three trace elements (Fe, Mn, As) that are commonly enriched in hydrothermal fluids. The thermodynamic calculations can be used to link the energetic potential of microbial communities to molecular evidence of their identities and metabolic capacity.

## Materials and methods

### Field work and chemical analyses

**Fig 1:**
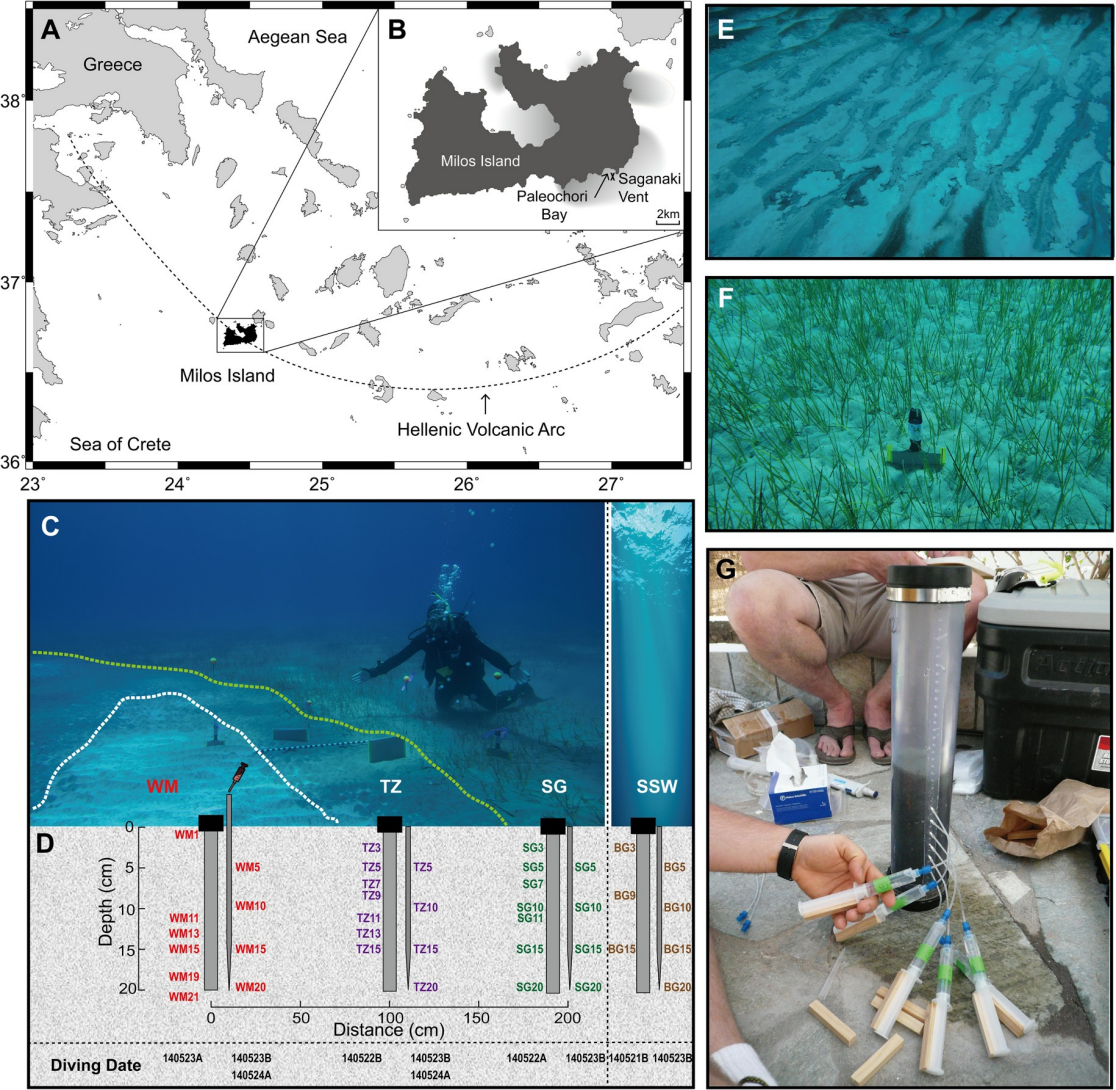
Site map. (A) and (B) Location of the Saganaki diffuse vent in Paleochori Bay (~300 m offshore, 12 m water depth). (C) Photograph of Saganaki showing three of the biogeographic zones: white mat (WM), transition zone (TZ), and seagrass area (SG). (D) Schematic of sampling methods (push cores and long pipettes indicating the position and depth (in cm) of samples collected for geochemistry and sequencing analysis. (E) Photograph of white mat area. (F) Photograph of seagrass area. (G) Photograph of porewater sampling from sediment cores using rhizons.

Samples were collected in May 2014 from Paleochori Bay, Milos Island (Greece) under a permit from the Greek Ephorate of Underwater Antiquities. In this study, we investigated samples from the Saganaki diffuse vent (36°40’24N, 24°30’50E), located under ~12 m of water and ∼300 m offshore (Fig 1A and 1B). Milos Island is located in the South Aegean Sea and is part of the Hellenic Volcanic Arc [50, 51] (Fig 1A). The volcanic activity that is responsible for active gaseous hydrothermal venting on and around the island has occurred since the Pliocene. The field sampling, sample preservation, and analytical protocols used in this study were based on those described in detail elsewhere [36, 38, 47, 49, 52–54], with minor modifications for our specific field location.

A SCUBA diving team measured *in situ* temperatures and collected fluid (via long pipette) and gas samples from the vent system. Large volumes of fluids with coarse resolution were collected under water through long pipette tips inserted directly into the sediments (~5 cm depth interval, from 5 to 20 cm deep) and attached to 60-mL syringes (Fig 1C and 1D). Free gas samples were obtained with a stainless-steel funnel placed on sites with visible gas bubbles. Glass serum bottles with blue rubber stoppers were filled with seawater before the dive and then connected to the top of the funnel. Once the gas completely replaced the seawater and flushed through for approximately 5 minutes, the valves were closed. Sediment cores (in polycarbonate tubes) were collected and sealed underwater with rubber caps. Sites included the center of a white mat (WM), through a transition zone (TZ) and a sea grass-covered region (SG), ending in a background area (BG) (Fig 1C, 1D, 1E, and 1F).

On shore, waters were carefully transferred into pre-cleaned serum bottles and capped without trapping any air (for dissolved gas analyses) or filtered (0.2 μm) and stored as described below for other analyses. Rhizons (0.2 μm filter) were inserted into pre-drilled holes of the polycarbonate tubes at 2 cm intervals from 0 to 20 cm to obtain high-resolution samples while avoiding fluid reflux from different depths (Fig 1G). All porefluids were analyzed for pH and then subsampled and treated for later geochemical measurements. The subsamples were stored in acid-washed polypropylene bottles for organic acid and anion analyses; acidified with 2% ultrapure HNO_3_ in acid-washed plastic bottles for cation analyses; acidified with 2% HCl and kept in opaque acid-washed glass bottles for arsenic speciation; fixed with zinc acetate solution for sulfide analysis; and stored in acid-washed and combusted glass vials for dissolved organic carbon (DOC) and total dissolved nitrogen (TDN) analyses.

Samples for major anions (F^-^, Cl^-^, Br^-^, SO_4_^2-^, NO_3_^-^, NO_2_^-^, PO_4_^3-^) were analyzed on a Metrohm 850 Professional ion chromatograph (IC). Major and minor cation (Li^+^, Na^+^, K^+^, Mg^2+^, Ca^2+^, Sr^2+^, Ba^2+^, Si^2+^, B^3+^, Mn^2+^, Fe^2+^) samples were measured on a Perkin-Elmer Optima inductively coupled plasma atomic emission spectrometer (ICP-OES). Samples set aside for arsenic (As^3+^, As^5+^, monomethylarsonic acid (MMA), and dimethylarsinic acid (DMA)) concentrations were analyzed on a Dionex ion chromatograph coupled to a PSAnalytical atomic fluorescence spectrometer (IC-AFS). Sulfide samples were analyzed by spectrophotometry with the Cline method. Dissolved organic carbon (DOC) was determined using high-temperature combustion on a Shimadzu Total Organic Carbon Analyzer (TOC-V) at the DOM Analytical Lab, Marine Science Institute, University of California-Santa Barbara. The dissolved gases were extracted from water samples after equilibrium was attained between the water sample and a known volume of high purity argon, which was injected directly into the serum bottles. Both free and dissolved gases were measured with a Shimadzu GC-2014ATF headspace gas chromatograph equipped with TCD and FID detectors.

### Thermodynamic modeling

The maximum amount of available energy from potential chemolithoautotrophic reactions at the temperature, pressure, and chemical composition of interest is given by the Gibbs energy (Δ*G*_*r*_). Values of Δ*G*_*r*_ were calculated using the relation

$$\Delta G_{r}=\Delta G_{r}^{0}+RTlnQ_{r}$$ (1)

where Δ*G*_*r*_^0^ denotes the standard state Gibbs energy of reaction, *R* designates the universal gas constant, *T* stands for the temperature in Kelvin, and *Q*_*r*_ represents the reaction quotient. Values Δ*G*_*r*_^0^ were calculated at the temperatures and pressures of interest with the revised-Helgeson-Kirkham-Flowers (HKF) [55–57] equations of state using OrganoBioGeoTherm (OBIGT)—which is a user-friendly version of the SCUPCRT92 software package [58]—and thermodynamic data from several sources [56, 59–65].

Values of *Q*_*r*_ were calculated with

$$Q_{r}=\prod a_{i}^{v_{i,r}}$$ (2)

where *a*_*i*_ designates the activity of the *i*^th^ species raised to its stoichiometric reaction coefficient *v*_*i*,*r*,_ in the *r*^th^ reaction, which is positive for products and negative for reactants. The activities of pure minerals (pyrite (FeS_2_), elemental sulfur (S^0^), magnetite (Fe_3_O_4_), hematite (Fe_2_O_3_), goethite (FeOOH), ferrihydrite (FeOOH), and pyrolusite (MnO_2_)) and water are taken to be unity (*a*_*i*_ = 1). Molalities of the *i*^th^ species in solution (*m*_*i*_) were obtained as noted above, and converted into activities using the individual activity coefficient of the *i*^th^ species, *γ*_*i*_:

$$a_{i}=m_{i}\gamma_{i}$$ (3)

Values of activity coefficients were calculated using the program SPEC8 (Geochemist’s Workbench Version 11, Aqueous Solutions LLC) employing the extended Debye-Hückel equation [66]. Aqueous activities of dissolved gases (H_2_, CH_4_, O_2_, CO, CO_2_, CH_4_) were calculated from free gas composition data assuming equilibrium. The reactions under consideration include numerous potential electron acceptors (O_2_, CO, CO_2_, HCO_3_^-^, N_2_, NO_2_^-^, NO_3_^-^, pyrite, elemental sulfur, magnetite, hematite, goethite, ferrihydrite, pyrolusite, H_2_AsO_4_^-^,) and donors (H_2_, CH_4_, CO, NH_4_^+^, N_2_, NO_2_^-^, H_2_S, pyrite, elemental sulfur, magnetite, Mn^2+^, H_3_AsO_3_,) (S1 Table). To permit us to evaluate the energetics of both the forward and reverse direction of every reaction, we also include H_2_O as a potential electron acceptor (where H in H_2_O can be reduced) and electron donor (where O in H_2_O can be oxidized).

To facilitate comparisons among reactions, values of Δ*G*_*r*_ were normalized to the number of moles of electrons transferred in the redox process [14]. In order to scale energy availability to the limiting reactant, the Gibbs energies are also presented in terms of energy densities, *E*_*r*_ [67]. To normalize the *E*_*r*_ on a ‘per kg of venting fluid’ and on a ‘per kg of sediment’ basis, values of Δ*G*_*r*_ were multiplied by the concentration of the limiting reactant in the fluid. The energy densities in fluid (*E*_*fluid*_) were calculated by

$$E_{fluid}=\left|\frac{\Delta G_{r}}{V_{i}}\right|\left[m_{i,\;fluid}\right]$$ (4)

where [*m*_*i*,*fluid*_] refers to the molal concentration of the *i*^th^ limiting electron donor or acceptor per kg of fluid, taking the stoichiometry of the reaction into account. The energy densities in sediment (*E*_*sediment*_) were calculated with

$$E_{sediment}=\left|\frac{\Delta G_{r}}{V_{i}}\right|\left[m_{i,\;sediment}\right]$$ (5)

where [*m*_*i*, *sediment*_] is the molal concentration of the *i*^th^ limiting electron donor or acceptor per kg of sediment, considering the porosity of sediments and the density of grains in them (Table 1). We did not evaluate the energy densities for reactions in which solid phases serve as both electron donor and acceptor (Reactions K66-68, L38-43, N17-19, O33-38, P33-38, Q33-38, R16-20, R25-27).

**Table 1:** Selected sediment properties and concentrations of Fe, Mg and S in solid phases in the Milos shallow-sea hydrothermal system.

Parameter		Reference
Porosity (%)	36	[68]
Grain density (g/cm^3^)	2.66	[68]
Wet density (g/cm^3^)	2.07	This study
Mean composition of clay pelites (%)	6.53	[69]
Mean Fe concentration in pelites (%)	3.4	[69]
Mean Mn concentration in pelites (ppm)	1685	[69]
Total Fe in sediment (%)	0.22	This study
Total Mn in sediment (%)	0.011	This study
Total S in 1g dry sediment	10 μM	Unpublished data

### Mixing model

Values of Δ*G*_*r*_ for all of the reactions listed in S1 Table were also calculated for different mixing ratios of end-member hydrothermal fluid (HF, also referred to as vent fluid) with seawater. The composition of the end-member HF was taken to be the average of white mat samples, and that for seawater was taken from Table 2. The activities of species that are very low in the end-member HF (e.g., oxygen) were taken to be 10^−9^. Although the compositions of the mixed fluids are simply proportional to the ratio of end-member fluid to seawater, the temperatures of the mixed fluids (*T*_*mix*_) are not a linear combination of the source fluids due to their differing heat capacities. These values were calculated using

$$T_{mix}=\frac{\sum_{i}m_{i}C_{pi}T_{i}}{\sum_{i}m_{i}C_{pi}}$$ (6)

where *m*_*i*_, *C*_*pi*_, and *T*_*i*_ refer to the mass, specific heat capacity, and temperature (K) of the *i*^th^ fluid. Values of *C*_*pi*_ were calculated using the equations of state for water in SUPCRT92 [70, 71]. The temperature of the end-member HF was estimated by extrapolation using the [Mg] = 0 method [72, 73].

**Table 2:** Temperature, pH and composition of porefluids and seawater sampled at or near the Saganaki diffuse vent, Milos Island, Greece.

Date-Dive-Type-Depth(cm)	T	pH	Na^+^	K^+^	Mg^2+^	Ca^2+^	Sr^2+^	Ba^2+^	Fe^2+^	Mn^2+^	As^3+^	As^5+^	Cl^-^	Br^-^	NO_2_^-^	NO_3_^-^	SO_4_^2-^	SiO_2_	H_2_S/HS^-^	DOC
	oC		mM	mM	mM	mM	μM	μM	μM	μM	μM	μM	mM	mM	mM	mM	mM	mM	μM	μM
**White Mat**																				
140523A-WM1		6.55	531.0	11.0	58.8	11.9	99.4	0.32	1.84	1.79			610.7	1.51	b.d.	b.d.	26.70	0.21	3.84	
140523A-WM5		5.37	498.9	12.4	53.8	11.9	91.3	0.75	1.91	28.31			573.7	0.72	b.d.	b.d.	25.97	2.72	324.67	
140523A-WM11	39.5	4.97	490.4	12.4	52.9	11.9	91.7	0.74	1.16	29.56	0.24	b.d.	564.0	0.72	b.d.	b.d.	24.29	2.95	1402.31	
140523A-WM13		4.89	496.6	13.5	53.0	12.6	96.6	0.67	0.34	30.75	0.23	b.d.	571.1	0.71	b.d.	b.d.	28.27	2.89	1115.29	
140523A-WM15	47.5	4.86	499.3	13.2	53.7	12.5	95.7	0.69	0.28	28.42	0.17	b.d.	574.2	0.74	b.d.	b.d.	24.07	2.90	1366.07	
140523A-WM19	53.0	5.12	477.1	12.5	51.6	11.9	91.6	0.70	0.43	27.11	0.24	b.d.	548.7	0.71	b.d.	b.d.	28.04	2.77	711.94	
140523A-WM21		4.94	471.9	11.9	51.6	11.7	90.0	0.61	0.15	24.57			542.7	0.74	b.d.	b.d.	24.46	2.70	1169.07	
140523B-WM5	51.8	4.56	401.2	25.8	26.6	15.0	115.1	1.13	93.43	78.30	0.13	0.002	461.4	0.60	0.16	b.d.	14.35	3.73		960.40
140523B-WM10	66.7	4.44	402.3	25.9	26.7	14.9	115.1	1.23	95.65	73.63	0.23	0.003	462.6	0.62	0.17	b.d.	13.73	3.70		1133.67
140523B-WM15	72.6	4.66	412.1	22.7	31.9	13.4	107.1	1.10	69.98	51.06	0.44	0.010	473.9	0.66	0.18	b.d.	17.38	3.25		1043.87
140523B-WM20	76.2	4.59	414.2	23.7	30.9	14.0	110.7	1.17	65.50	53.99	0.61	0.003	476.3	0.67	0.15	b.d.	13.39	3.37		1325.41
140524A-WM5	38.9	4.71	411.3	10.4	44.6	8.1	66.0	0.60	6.99	44.80	0.32	0.002	473.0	0.60	0.16	b.d.	23.17	3.05	991.53	1036.98
140524A-WM10	57.6	4.70	410.7	10.1	44.5	8.1	66.2	0.68	6.09	43.14	0.28	0.001	472.3	0.62	0.16	b.d.	22.83	3.02	838.92	1086.84
140524A-WM15	66.5	4.68	409.6	10.2	44.4	8.1	65.9	0.71	5.80	42.97	0.27	0.001	471.0	0.66	0.18	b.d.	21.37	3.03	1037.74	1203.65
140524A-WM20	71.3	4.67	413.6	10.0	44.9	8.2	66.9	0.81	7.07	40.25	0.24	0.001	475.6	0.67	0.17	b.d.	20.81	2.94	1631.73	1198.96
**Transition Zone**																				
140522B-TZ3	19.1	5.56	522.4	10.7	58.3	11.7	98.4	0.24	69.13	3.22	0.94	b.d.	600.8	0.90	b.d.	b.d.	33.00	0.72	11.02	
140522B-TZ5	23.0	5.64	520.5	10.6	58.1	11.7	98.1	0.30	50.58	3.32	0.37	b.d.	598.6	0.75	b.d.	b.d.	32.36	1.04	469.85	
140522B-TZ7	25.0	5.27	518.4	10.6	58.0	11.6	97.7	0.34	11.17	3.21	0.85	b.d.	596.2	0.79	b.d.	b.d.	34.45	1.21	823.59	
140522B-TZ9	27.0	5.21	521.1	10.6	58.4	11.7	99.2	0.36	2.77	3.32	0.66	b.d.	599.3	0.77	b.d.	b.d.	33.17	1.32	275.05	
140522B-TZ11	28.0	5.19	516.2	10.4	57.9	11.6	97.9	0.35	0.37	3.42	0.84	b.d.	593.6	0.77	b.d.	b.d.	33.00	1.36	729.99	
140522B-TZ13	30.0	5.12	526.1	10.6	59.0	11.8	99.8	0.44	0.99	3.01	1.40	b.d.	605.0	0.74	b.d.	b.d.	32.37	1.29	1228.93	
140522B-TZ15	31.0	5.12	524.3	10.6	58.8	11.8	99.5	0.38	0.22	3.73	0.47	b.d.	602.9	0.74	b.d.	b.d.	31.57	1.40	1162.86	
140523B-TZ5	23.1	5.15	518.4	10.5	57.6	12.1	96.6	0.39	8.10	7.91	0.06	0.001	596.2	0.77	0.22	b.d.	28.77	1.59		1122.10
140523B-TZ10	27.2	4.88	528.0	10.8	58.4	12.4	99.2	0.41	0.28	9.46	0.04	b.d.	607.2	0.78	0.23	b.d.	28.55	1.88		1168.38
140523B-TZ15	31.0	4.87	518.2	10.6	57.3	12.2	97.4	0.40	b.d.	9.25	0.02	b.d.	595.9	0.77	0.21	b.d.	25.97	1.86		1233.12
140523B-TZ20	34.2	4.76	512.3	10.5	56.7	12.1	96.0	0.39	b.d.	9.32	0.03	b.d.	589.1	0.77	0.21	b.d.	29.50	1.90		1190.97
140524A-TZ5	21.9	5.00	513.5	10.4	57.3	11.6	96.1	0.25	9.20	2.38	0.10	0.001	590.5	0.77	0.21	b.d.	27.99	0.78	896.83	1287.39
140524A-TZ10	23.9	4.77	512.2	10.5	57.0	11.6	96.8	0.33	2.36	2.67	0.08	0.001	589.0	0.78	0.21	b.d.	26.87	1.22	825.40	1133.40
140524A-TZ15	25.9	4.79	503.9	10.3	56.1	11.4	96.0	0.34	4.16	2.45	0.11	0.001	579.5	0.77	0.23	b.d.	28.66	1.26	1619.58	1063.70
140524A-TZ20	28.5	4.80	511.2	10.4	56.8	11.6	97.1	0.36	2.03	2.39	0.18	0.002	587.9	0.77	0.21	b.d.	24.74	1.30	1081.85	1188.49
**Seagrass**																				
140522A-SG3	19.1	7.04	530.8	11.7	59.4	11.6	98.6	0.14	23.52	1.08	0.50	0.058	610.4	0.93	b.d.	b.d.	26.47	0.02	0.28	
140522A-SG5	19.1	7.12	525.7	11.6	58.9	11.5	98.1	0.20	11.21	0.82			604.6	0.87	b.d.	b.d.	25.97	0.02	1.27	
140522A-SG7	19.1		538.3	12.1	59.4	11.7	99.5	0.16	11.05	1.12	0.66	0.053	619.0	0.91	b.d.	b.d.	25.41	0.02	0.21	
140522A-SG11	19.1	7.13	538.1	12.1	59.5	11.7	99.0	0.18	12.01	0.91	0.41	0.077	618.8	0.94	b.d.	b.d.	26.31	0.03	0.42	
140522A-SG15	19.1	7.05	535.6	11.9	59.3	11.7	99.3	0.19	11.84	0.86	1.16	0.135	615.9	0.01	b.d.	b.d.	25.58	0.03	0.78	
140523B-SG5	19.8	6.80	534.3	11.8	59.1	11.6	98.7	0.13	14.38	0.83	0.49	0.001	614.4	0.87	0.21	b.d.	26.36	0.03	1.20	1219.35
140523B-SG10	20.3	6.54	534.9	11.8	59.3	11.6	98.5	0.15	17.02	1.17	0.41	0.001	615.1	0.98	0.23	b.d.	28.04	0.04	0.69	1210.81
140523B-SG15	20.8	6.84	530.1	11.7	58.7	11.5	98.2	0.16	20.56	1.36	0.41	0.017	609.6	0.86	0.23	b.d.	28.27	0.05	2.84	1160.12
140523B-SG20	21.3	6.93	536.4	11.9	59.3	11.7	98.8	0.18	21.20	1.51	0.45	0.011	616.9	0.90	0.20	b.d.	25.69	0.05	1.89	1323.48
**Background**																				
140521B-BG3	19.1	6.34	524.8	11.4	58.7	11.4	97.1	0.08	b.d.	b.d.	0.10	b.d.	603.5	0.90	b.d.	b.d.	32.31	b.d.	0.21	481.33
140521B-BG9	19.1	6.75	527.4	11.5	59.1	11.5	98.2	0.13	2.28	0.07	0.01	b.d.	606.5	0.84	b.d.	b.d.	29.81	0.02	b.d.	773.76
140521B-BG15	19.2	6.64	528.4	11.5	59.1	11.5	98.6	0.15	14.74	0.39	0.17	b.d.	607.7	0.90	b.d.	b.d.	32.05	0.04	1.41	649.64
140523B-BG5	19.2	6.88	531.4	12.0	58.6	11.6	98.6	0.12	0.83	2.07	0.21	0.002	611.1	0.91	0.21	b.d.	23.39	0.05	0.19	1198.69
140523B-BG10	19.1	6.62	529.2	11.6	58.6	11.5	97.3	0.11	b.d.	0.18	0.04	0.001	608.6	0.82	0.23	b.d.	30.14	0.01	0.32	1134.78
140523B-BG15	19.1	7.09	527.7	11.6	58.5	11.5	97.6	0.13	0.34	0.05	0.06	0.002	606.9	0.81	0.20	b.d.	21.71	0.02	0.06	1019.07
140523B-BG20	19.0	7.14	539.2	11.9	59.6	11.7	99.2	0.15	1.33	0.03	0.09	0.003	620.1	0.92	0.21	b.d.	25.75	0.03	0.26	1089.60
**Seawater**																				
140525A-SW	19.0	7.4	528.5	11.6	59.2	11.6	98.5	0.06	b.d.	0.2	0.1	b.d.	607.8	1.01	0.23	b.d.	28.4	0.01	0.100	528.85
**Calculated Endmember**																				
Average	199.41	4.12	119.98	29.56	0.00	11.57	61.04	3.75	121.82	204.17	0.79	0.01	18.09	0	0	0	0	14.73	5180.57	-

b.d. = below detection.

## Results and discussion

### Sample location and geochemistry

**Fig 2:**
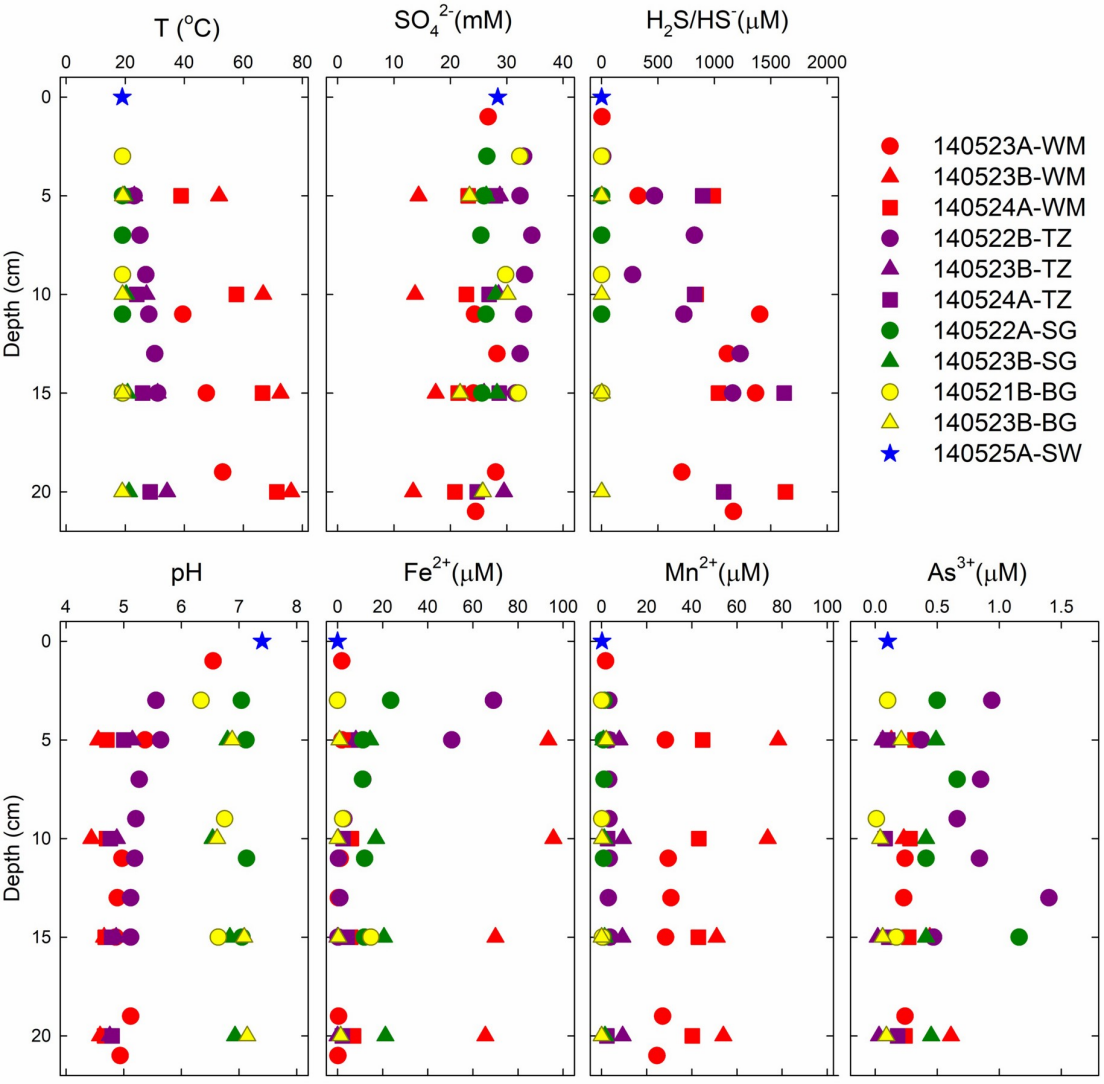
Geochemical profiles of temperature, pH, and selected ions. Red symbols refer to white mat (WM), purple to transition zone (TZ), green to seagrass (SG), yellow to background (BG) and blue to surface seawater (SW). Different symbols of the same color indicate different date/time of the sampling dives.

The 47 fluid samples from the Saganaki vent area (Fig 1B and Table 2) represent different mixing ratios and physicochemical properties of venting fluids in this system. The hottest area (up to 76.2°C) was covered by a ~1 cm thick, fluffy, white mat (WM) (Figs 1C, 1E, 2 and Table 2). A ~1 m wide transition zone (TZ) separates a flourishing seagrass (SG) area from the diffuse venting site (Fig 1C). Fifteen samples were taken from WM, 15 from TZ, 9 from SG, 7 from background sediment (BG) and 1 from surface seawater (SW).

Fluid measurements (Fig 2 and Table 2) reveal a wide range of temperature (19.1–76.2°C) and pH (4.4–7.4), as well as sharp differences in geochemistry for the five sampling regions. For example, concentrations of SO_4_^2-^ (13.4–34.5 mM), Na^+^ (401.2–539.2 mM), and Mg^2+^ (26.6–59.6 mM) increase as a function of distance from the diffuse vent in the WM area. In contrast, K^+^ levels were almost twice as high (22.7–25.9 mM) at WM than at other sites (10.3–12.1 mM). NO_3_^-^ concentrations were below detection (b.d., 8 μM) in all samples, but NO_2_^-^ levels were relatively high (0.15–0.23 mM) in several samples. We note that very low nitrate levels (< 0.5–3.5 μM) have previously been reported in Mediterranean surface waters, especially around Greece [74], and near mM concentrations of nitrite are known to occur in marine sediments [75]. Concentrations of reduced species, such as H_2_S, Fe^2+^, and Mn^2+^ range from b.d. (1 μM, 0.1 μM, and 0.04 μM, respectively) to relatively high levels (93 μM, 78 μM, and 1631 μM, respectively). Consistent with the stable and conservative nature of salinity in seawater (i.e., the principle of constant proportions), and to maintain required charge balance, concentrations of Cl^-^ in Table 2 were calculated from a well-established Cl^-^:Na^+^ ratio of 1.15 (e.g., [76]).

### Porefluid energetic potential

**Fig 3:**
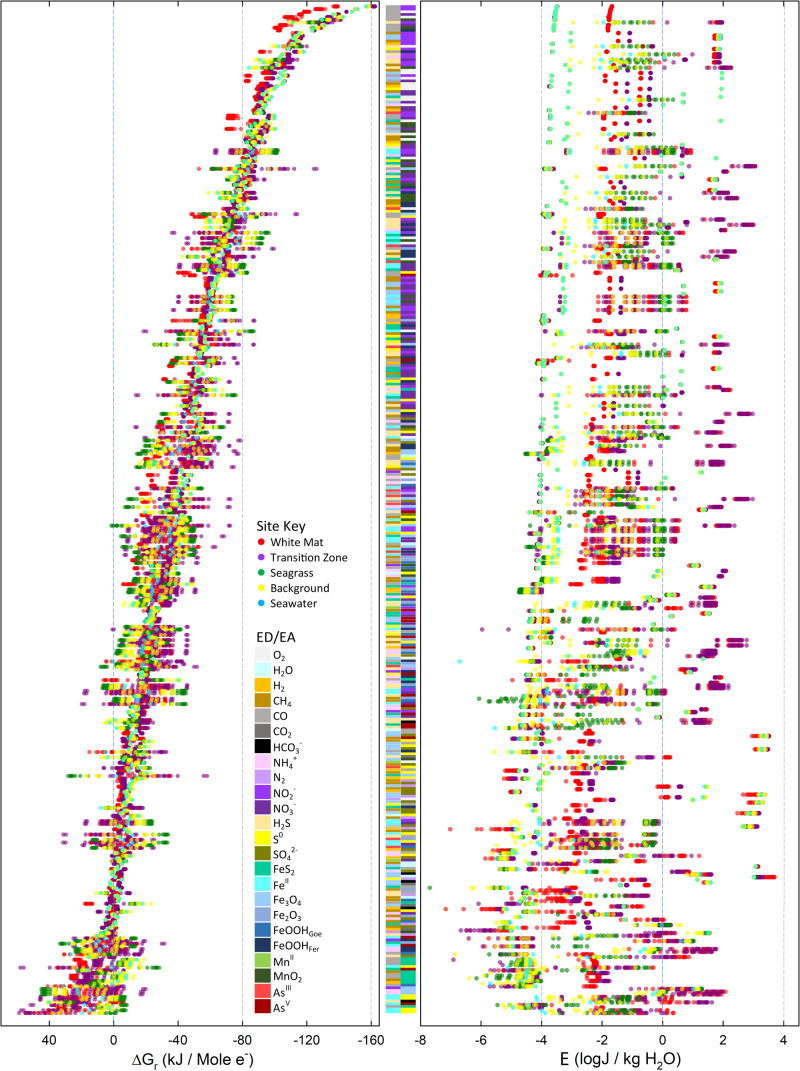
Overall Gibbs energy yields of the catabolic reactions considered in this study. Circles refer to values of Δ*G*_*r*_ of the 379 exergonic reactions shown in S2 Table at individual sample sites in units of kJ/mol e^-^ (A) and J/kg fluid (B). The colors of the circles encode for the five biogeographic regions (see key). The colored bars in the middle of the two panels refer to the identities of electron acceptors and donors in the reactions as noted in the key. The reactions are ordered from most exergonic at the top to least exergonic at the bottom, based on the averages of Δ*G*_*r*_ values from all samples.

Values of Δ*G*_*r*_ for the 730 reactions listed in S1 Table were calculated with Eqs (1–4) using the fluid compositions and environmental conditions given in Tables 2 and 3. Values of Δ*G*_*r*_ in kJ per mole of electron transferred are depicted in Fig 3A for the 379 reactions that are exergonic (Δ*G*_*r*_<0) in at least one sampling location. The reactions are plotted from top (-162.3 kJ/mole e^-^) to bottom (near 0 kJ/mole e^-^) in order of the average energy yield at all 47 sampling sites. It can clearly be seen that for most reactions, the energy yields vary by 20–50 kJ/mol e^-^ across the different locations and sample depths; in a number of examples, especially reactions with iron (e.g. L34: Fe^2+^*/*S^0,^ and N16: H_2_S/Fe_3_O_4_), the range approaches and even exceeds 80 kJ/mol e^-^. Reactions with nitrite (purple bar) and oxygen (light grey bar) as electron acceptors and with CO as electron donor are the most exergonic, with the top 10 reactions (electron donor/acceptor) being CO/NO_2_^-^ (reaction H13, -162.3 kJ/mole e^-^), CO/NO_2_^-^(H14, -151.4 kJ/mole e^-^), CO/O_2_ (B5, -147.9 kJ/mole e^-^), CO/NO_3_^-^ (I18, -139.9 kJ/mole e^-^), CO/O_2_ (B6, -142.6 kJ/mole e^-^), CO/MnO_2_ (R5, -140.9 kJ/mole e^-^), H_2_S/NO_2_^-^ (H19, -136.0 kJ/mole e^-^), CO/NO_3_^-^ (I19, -131.0 kJ/mole e^-^), CO/MnO_2_ (R6, -130.7 kJ/mole e^-^), CO/FeOOH_Fer_ (Q13, -124.9 kJ/mole e^-^) (Fig 3A).

**Table 3:** Dissolved and free gas composition in white mat (WM), transition zone (TZ) and background (BG) areas.

Sample	H_2_	O_2_	N_2_	CO_2_	CH_4_	CO
**WM Free Gas (%)**	b.d.	2.72	18.00	61.36	0.299	0.025
**WM Dissolved Gas at 10cm (μM)**	b.d.	0.496	38.5	11.93	0.038	0.076
**TZ Dissolved Gas at 5cm (μM)**	b.d.	1.001	76.6	2.725	0.089	b.d.
**BG (μM)**	b.d.	220	456.00	2.638	b.d.	b.d.
**End-member (average) (μM)**	0	0	0	47.304	0.183	0.365

**Fig 4:**
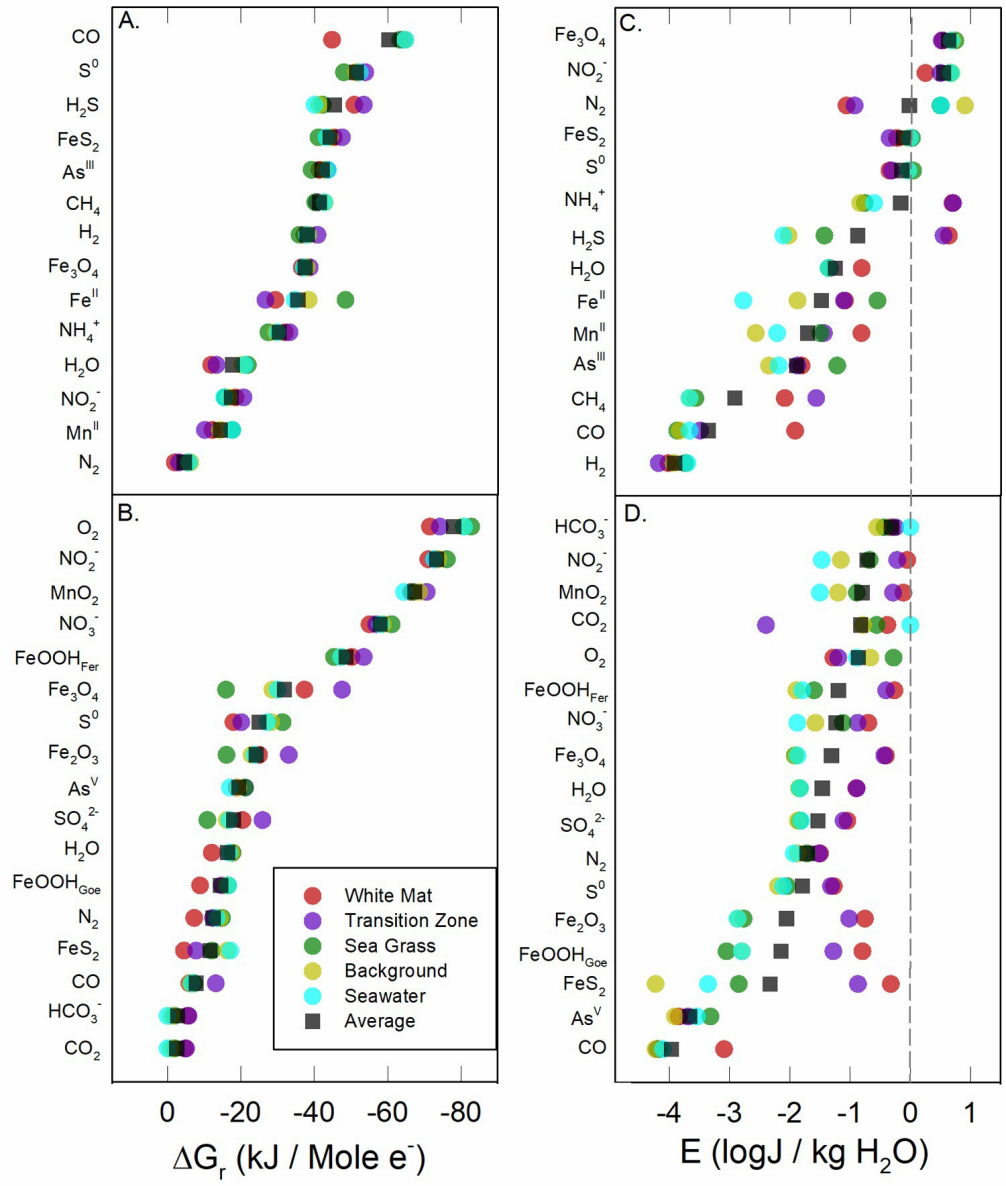
Average Gibbs energy yields of exergonic reactions. Average values of Δ*G*_*r*_ (panels A and B, in kJ/mol e^-^) and *E*_*fluid*_ (panels C and D, in J/kg H_2_O) grouped by electron donors (panels A and C) and electron acceptors (panels B and D) for the five biogeographic regions considered in this study.

In Fig 3B, energy yields are plotted as energy densities (*E*_*fluid*_). Again, the range of energy yields across sites and sample depths vary tremendously, often exceeding 6 orders of magnitude in J/kg H_2_O. This broad range is due to the rapid dilution of hydrothermal fluid with seawater, whereby the concentrations of some key redox species change by 2–3 orders of magnitude over short distances (Table 2). For example, the concentration of Mn in WM (28.31–78.30 μM) is 150–400 times that in seawater, and therefore *E*_*fluid*_ of Mn-redox reactions point to a much larger energy potential than the corresponding value of Δ*G*_*r*_ would indicate. In addition, one of the consequences of setting the activities of pure minerals to 1.0 means that energy densities of reactions involving hematite, goethite, ferrihydrite, pyrolusite, pyrite, elemental sulfur, and magnetite are high (100–10,000 J/kg H_2_O). Finally, we note that the most exergonic reactions in Fig 3B are not the same as those in Fig 3A. In terms of energy density, the oxidation of sulfide, sulfur, and ammonia are thermodynamically most favorable. A phylogenetic analysis (16S rRNA) of hydrothermal sediments at Milos showed that heterotrophs and sulfur oxidizers were among the most abundant [77]. In fact, several aerobic and anaerobic sulfur oxidizers have been isolated from Milos, including *Halothiobacillus kellyi* [78], *Stetteria hydrogenophila* [79], *Thiomicrospira sp*. Milos-T1 [80], and *Gamma Proteobacteria* Milos strain ODI4G, OBII5, ODIII6, OBII5 [81].Values of Δ*G*_*r*_ and *E*_*fluid*_ for the exergonic reactions shown in Fig 3 are re-plotted as colored circles in Fig 4, but with average values for each of the five regions and classified by electron donors (Fig 4A and 4C) and electron acceptors (Fig 4B and 4D). In addition, the total average energy yields for all 47 samples are also plotted (black squares). In terms of Δ*G*_*r*_, reactions with O_2_ as the electron acceptor supplied the most energy, followed by reactions with NO_3_^-^, NO_2_^-^, and MnO_2_, and then reactions with iron minerals, S^0^, As^V^, SO_4_^2-^, and inorganic carbon. In units of energy density (*E*_*fluid*_), reactions with ammonia and sulfide as electron donors are the most exergonic, especially in the WM and TZ regions (Fig 4C). When color-coded by electron acceptor (Fig 4D), we note that values of *E*_*fluid*_ are generally highest (with some exceptions) in the WM region, followed by the TZ, SG, BG, and SW. See LaRowe and Amend (2019) for a discussion on reaction energetics in molal versus density units [82]. This difference can best be seen in reactions with CO, where energy values are -132 to -162 kJ/mol e^-^, but only 1.7 to 3.5 J/kg H_2_O (e.g. reaction H13). Similarly, for the oxidation of methane, carbon monoxide, and ammonium, values of the energy densities are more exergonic than the per-electron counterparts reveal, particularly in the WM and TZ regions. Because the energy densities of sulfide oxidation are so variable, it is likely that the importance of this process is highly localized, with notable potential in the more hydrothermally influenced areas (WM, TZ). Energy densities for iron, manganese, and arsenite oxidation reactions are also scattered, while those for the oxidation of elemental sulfur, pyrite and magnetite show no clear trends. Energy densities for the WM and TZ regions appearing on the right hand (i.e., more exergonic) side of Fig 4D indicates that chemolithotrophic primary production has a positive correlation with temperature, or hydrothermal source.

Similar studies of redox reaction energetics for putative chemotrophic metabolisms have been carried out for geochemically diverse hot springs in Yellowstone National Park [26], shallow-sea hydrothermal systems in the Aeolian Islands (Italy) [22, 38] and the continental subsurface at the Sanford Underground Research Facility (SURF) in South Dakota (USA) [83]. Analogous to the present study at Milos, those communications concluded that reactions with energy yields >100 kJ/mol e^-^ are rare and involve O_2_ or NO_3_^-^/NO_2_^-^ as electron acceptors. It should be noted that the maximum yields in the Milos system are at ~160 kJ/mol e^-^, while those in the Aeolian Islands are ~120 kJ/mol e^-^, in Yellowstone hot springs are ~110 kJ/mol e^-^, and at SURF are ~100 kJ/mol e^-^. Nine of the most exergonic reactions at Milos are aerobic or anaerobic carbon monoxide oxidation. We note two reasons for the higher Δ*G*_*r*_ yields at Milos than at other sites: First, CO and NO_2_^-^ have large and opposite redox potentials, which tends to lead to large Gibbs energies of reaction. Second, we measured and considered a larger range of redox sensitive species than most other studies, which rarely include CO and NO_2_^-^ because they decay quickly, or their concentrations are below detection limits.

This set of investigations also demonstrated that in a number of examples, changes in chemical composition—with pH being a major driver—can ‘flip’ a reaction from exergonic to endergonic. In other words, the forward reaction may serve as a putative metabolism in some environments, while the reverse direction could do so at very different geochemical conditions. Similarities among these different studies are also observed when energy densities are considered. (Note that studies focused on the shallow-sea vents at Vulcano, Aeolian Islands [22] and Yellowstone hot springs [26] did not provide such results. In each case, the most exergonic reactions in terms of energy density are different from those labeled as most exergonic in Δ*G*_*r*_ space.

### Bulk sediment energetic potential

**Fig 5:**
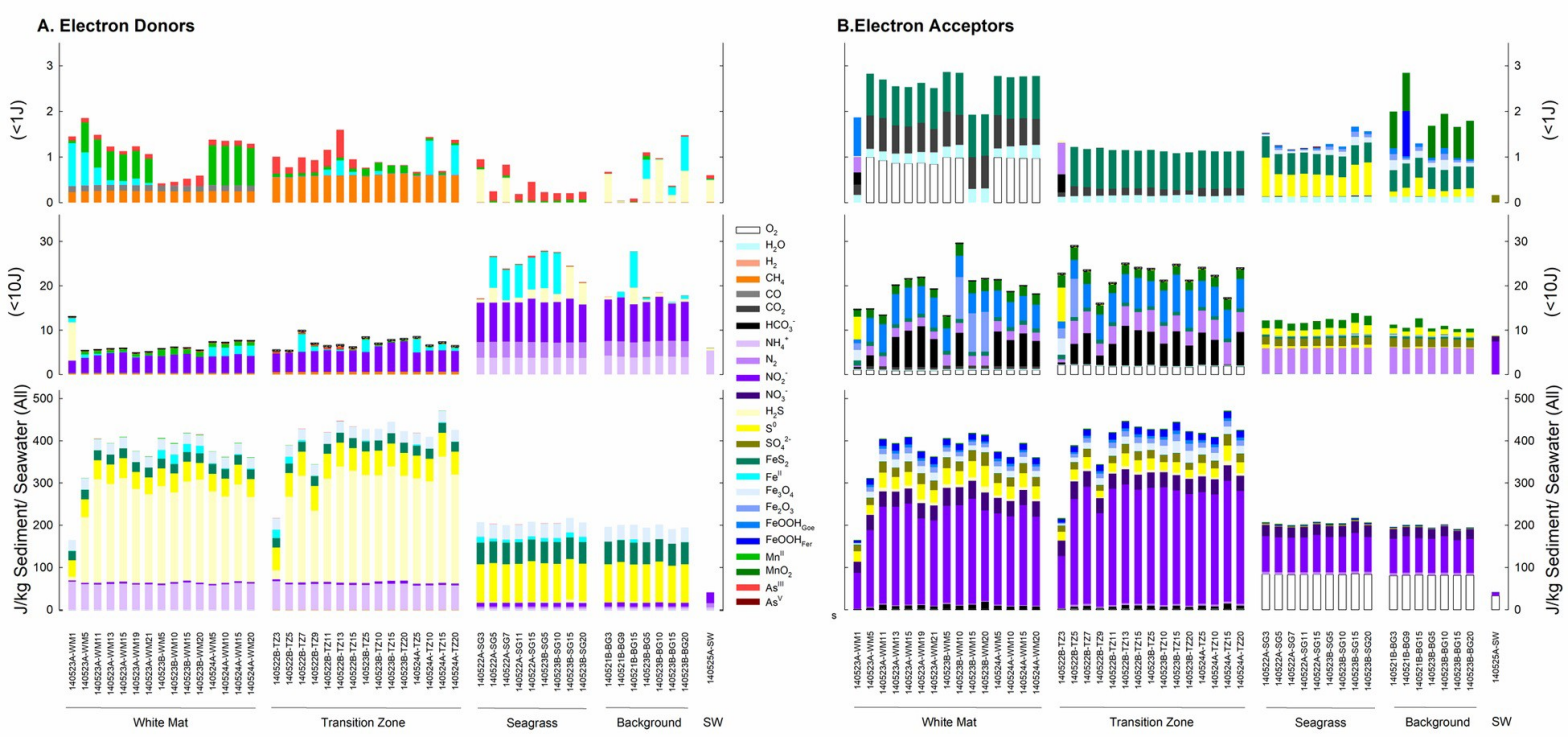
Energy densities at all sample locations and depths. Colored bars refer to identities of different electron donors (A) and electron acceptors (B). The upper and middle panels zoom in on reactions with low energy yields (<1 J and <10 J, respectively). The energy densities refer to either those in 1 kg sediment or 1 kg seawater. See S1 Table for details on the reactions.

The calculations summarized above only considered porefluids and not the solid phase minerals that can be used as energy sources. Following the same color scheme as in Fig 3, the amount of energy available per kg of sediment (*E*_*sediment*_, J/kg sediment) is shown in Fig 5A (by electron donor) and 4B (by electron acceptor). The bottom panels reveal total *E*_*sediment*_ for all reactions, while the middle and top panels show values of *E*_*sediment*_ <1 J/kg sediment and <10 J/kg sediment, respectively. It can be seen in the bottom panel in Fig 5A that reactions with ammonia, sulfide, sulfur, magnetite, and pyrite as electron donors are the most exergonic; the bottom panel in Fig 5B shows that reactions with nitrate, nitrite, sulfur and sulfate as electron acceptors provide the most energy. These panels reveal three energy-based habitats: (1) hydrothermal-influenced sediments in the WM and TZ regions, (2) seawater-influenced sediments in the SG and BG areas and (3) surface seawater. Energy yields are similar in the WM and TZ regions, increasing with depth at both locations. Conversely, depth is not a correlating factor in the SG and BG areas. Reactions that provide relatively small amounts of energy are shown in the middle and top panels in Fig 5, with patterns of energy yields changing with the distance from the diffuse vent. Both the WM and TZ settings show increasing energy yield with sediment depth, and the dominance of aerobic respiration in seawater-influenced sediments and seawater.

From an energetics perspective, oxygen/nitrate/nitrite and sulfur/sulfide/ammonia should be the main electron acceptors and donors, respectively, to be used by chemolithotrophs at Milos (Fig 5). Based on values of Gibbs energy and elevated levels of abundance, sulfide oxidation coupled to nitrite reduction could support much of the chemolithotrophic primary production. Some evidence, though limited, is available to support this claim: three chemolithotrophic nitrate-reducing sulfate-oxidizing bacteria have been isolated from Milos [81] and isotopic evidence of microbial sulfate reduction in the TZ has been reported [84].

### Mixed fluid (SW:HF) energetic potential

**Fig 6:**
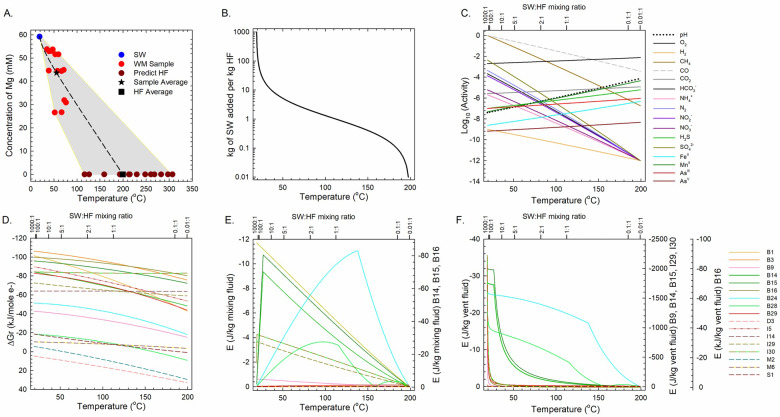
Temperature, composition and energetics resulting from the hydrothermal fluid-seawater mixing model. (A) Extrapolated temperature of end-member HF at [Mg] = 0 (brown circles) based on values of [Mg] in WM samples (red circles) and SW (blue circle). The temperature, composition and energetics of redox reactions resulting from fluid mixing were calculated using the average WM composition (black star) and average HF composition (black square). (B) Fluid temperature as a function of SW:HF mixing ratio. (C) Activities of redox-sensitive species as a function of temperature and SW:HF mixing ratio. Values of Δ*G*_*r*_ (D) and *E*_*fluid*_ (E, F) of exergonic reactions listed in Table 4. Because the results span several orders of magnitude, reactions B14, B15 and B16 have a different Y-axis scale on right side in (E); and reactions B9, B14, B15, I29, I30, and B16 refer to the two Y-axis scales on the right side in (F).

The estimated, weighted average temperature of the end-member HF, based on Mg content in 13 WM samples, is 199.4°C (Fig 6A). The curve shown in Fig 6B depicts the calculated temperature of a fluid that results from titrating cold seawater (SW) into 1 kg of this end-member HF, and Fig 6C presents how the activities of major redox-active species change with the mass ratios of SW:HF from 10^3^:1 to 10^−2^:1, as well as a temperature range from 19.0 to 199.4°C. The remaining panels in Fig 6 show how much energy is available from the 17 reactions listed in Table 4 normalized to kJ per mole electron transferred (Fig 6D), J per kg mixed fluid (Fig 6E), and J per kg vent fluid (Fig 6F), where solid lines refer to aerobic processes, dashed lines represent anaerobic processes, and the different colors identify different reactions.

**Table 4:** Catabolic reactions used to define metabolic groups in the analysis shown in Fig 5.

Metabolic Group	#	Reaction
Hydrogen Oxidizers	B1	2H_2_ + O_2_ →2 H_2_O
	I5	5H_2_ + 2NO_3_^-^ + 2H^+^ → N_2_ + 6H_2_O
Methane Oxidizers	B3	CH_4_ + 2O_2_ → CO_2_ + 2H_2_O
	I14	CH_4_ + 4NO_3_^-^ → 4NO_2_^-^ + CO_2_ + 2H_2_O
	M6	CH_4_ + SO_4_^2-^ + 2H^+^ → H_2_S + CO_2_ + 2H_2_O
Ammonium Oxidizers	B9	NH_4_^+^ + 2 O_2_ → NO_3_^-^ + 2 H^+^ + H_2_O
Sulfur Oxidizers	B14	2 H_2_S + O_2_ → 2 S + 2 H_2_O
	B15	H_2_S + 2 O_2_ → SO_4_^2-^ + 2 H^+^
	B16	2 S + 3 O_2_ + 2 H_2_O → 2 SO_4_^2-^ + 4 H^+^
	I29	5 H_2_S + 2 NO_3_^-^ + 2 H^+^ → 5 S + N_2_ + 6 H_2_O
	I30	5 H_2_S + 8 NO_3_^-^ → 5 SO_4_^2-^ + 4 N_2_ + 2 H^+^ + 4 H_2_O
Iron Oxidizers	B24	4 Fe^2+^ + O_2_ + 6 H_2_O → 4 FeOOH _Ferrihydrite_ + 8 H^+^
Manganese Oxidizer	B28	2 Mn^2+^ + O_2_ + 2 H_2_O → 2 MnO_2 Pyrolusite_ + 4 H^+^
Arsenite Oxidizers	B29	2 H_3_AsO_3_ + O_2_ → 2 H_2_AsO_4_^-^ + 2 H^+^
Methanogens	D3	4H_2_ + CO_2_ → CH_4_ + 2H_2_O
Sulfate Reducers	M2	4H_2_ + SO_4_^2-^ + 2H^+^ → H_2_S + 4H_2_O
Arsenate Reducers	S1	H_2_ + H_2_AsO_4_^-^ + H^+^ → H_3_AsO_3_ + H_2_O

Thermodynamic predictions of redox reaction energetics can change substantially when different normalization schemes are used [85, 86] For instance, when normalized to kJ per mole electron transferred (Δ*G*_*r*_/e^-^) for the SW:HF mixing calculations, the energy yields of all reactions decreased with increasing proportion of HF (Fig 6D). When normalized per kg of mixed fluid (*E*_*fluid*_), however, a very different picture emerges (Fig 6E); here, the reactions fall into two groups: three reactions (B14-B16) that can provide >60 J/kg mixed fluid at optimal SW:HF ratios, and the other 14 reactions where energy yields maximize at ~11 J/kg mixed fluid for one example (B24) and ~4 J/kg mixed fluid for the rest. Finally, the potential energy per kg of pure vent fluid is given in Fig 6F. In this normalization, the reactions are most exergonic at a SW:HF mixing ratio of ~100:1, corresponding to a temperature of ~20°C. At these conditions, the energy yields from sulfide oxidation with O_2_ or NO_3_^-^ (B14, B15, I29, I30) and aerobic ammonia oxidation (B9) exceed by several orders of magnitude those of the other reactions. A similar story emerges when normalizing values of Δ*G*_*r*_ per kg mixed fluid and per kg vent fluid for aerobic and anaerobic sulfur/sulfide oxidation in the WM area (Fig 6E and 6F). This can be seen most clearly at temperatures <40°C, where these reactions are 20–50 times as exergonic as the other reactions. As noted above, isotopic data also point to a dynamic sulfur cycle at Milos with microbial sulfate reduction, sulfide oxidation, and rapid recycling of sulfur intermediates that vary with location and time [47, 84].

The curves in Fig 6E (J/kg mixed fluid) and 6F (J/kg hydrothermal fluid) show how the energy densities of the reactions in Table 4 change with HF:SW ratio. The multiple y-axes are used in Fig 6E and 6F because the results span several orders of magnitude. It can clearly be seen that as the proportion of HF decreases, the energy yields for most reactions also decrease, especially when these are plotted per kg of vent fluid (Fig 6F). The kinks and inflection points in some of the curves in Fig 6E and 6F are a result of calculating the reaction energies using the concentration of the limiting electron donor or acceptor. These abrupt changes in slope correspond to points where the concentrations of the electron donors and acceptors are equal. The inflection points in Fig 6E also illustrate the temperatures at which the reactions are most exergonic. For example, the sulfur and sulfide oxidation reactions B14 and B15 have inflection points at 27°C, above which their potential energy yields decrease. Similarly, the limiting reactant for reactions B24 and B28 changes from iron and manganese, respectively, to oxygen as temperature increases (Fig 6E). Our mixing model suggests that microbial metabolic strategies often shift with mixing ratio, and therefore temperature, a notion that is supported by other lines of evidence. Note that cell abundances of sulfur oxidizing bacteria, sulfate reducing bacteria and dissimilatory iron-reducing bacteria at Milos decrease with increasing depth and temperature [87]. Also at Milos, microbial cell numbers are highest in the shallowest sediment [77].

### Conclusions

Seawater and porewater chemistries, a fluid mixing model, and thermodynamic calculations were combined to determine the energy yields of more than 700 redox reactions in fluids and sediments of a Milos Island shallow-sea hydrothermal environment. These yields were reported in several normalization schemes—in kJ per mole electrons transferred and in J per kg water or sediment—revealing potential chemolithotrophic microbial metabolisms as a function of depth and distance from a diffuse vent area. We demonstrated that in this system at Milos, analogous to other shallow-sea hydrothermal systems, hot spring environments, and the deep continental subsurface, a large number of inorganic redox reactions can be exergonic, suggesting that diverse chemolithotrophic metabolisms may be occurring simultaneously. Based on modeling of SW:HF mixed solutions in shallow sediments, we also showed that energy yields can change dramatically along the posited steep gradients in temperature, pH, and composition of redox-sensitive aqueous solutes, together with consideration of redox-sensitive minerals. In the Milos hydrothermal system, this applies to a transect from the White Mat area across the Transition Zone to the Seagrass and Background zones and into seawater. An environmental energy framework of the type provided here can help interpret biodiversity data and ecosystem function, and also guide efforts to cultivate dominant as well as important minor members of a chemolithotrophic microbial community.

## Supporting information

S1 Table Redox reactions considered in this study. (DOCX)

S2 Table Order of reactions shown in Fig 3 from top (1) to bottom (379). (DOCX)
