# Supplementary material for: Bioenergetic characterization of a shallow-sea hydrothermal vent system: Milos Island, Greece
Source: PLoS One. 2020 Jun 5;15(6):e0234175. doi: 10.1371/journal.pone.0234175 (PMC7274409; doi:10.1371/journal.pone.0234175)
Supplement: S1 Table — (DOCX) [file pone.0234175.s001.docx]

Table S1. Redox reactions considered in this study

| **#** | RXN. | e^-^ |
| --- | --- | --- |
|  | **Oxidation of C, N, S, Fe, Mn, and As Species (with H_2_O as Oxidant)** |  |
| **A1** | CH_4_ + H_2_O → CO + 3 H_2_ | 6 |
| **A2** | CH_4_ + 2 H_2_O → CO_2_ + 4 H_2_ | 8 |
| **A3** | CH_4_ + 3 H_2_O → HCO_3_^-^ + 4 H_2_ + H^+^ | 8 |
| **A4** | CO + H_2_O → CO_2_ + H_2_ | 2 |
| **A5** | CO + 2 H_2_O → HCO_3_^-^ + H_2_ + H^+^ | 2 |
| **A6** | NH_4_^+^ + 2 H_2_O → NO_2_^-^ + 3 H_2_ + 2 H^+^ | 6 |
| **A7** | NH_4_^+^ + 3 H_2_O → NO_3_^-^ + 4 H_2_ + 2 H^+^ | 8 |
| **A8** | N_2_ + 4 H_2_O → 2 NO_2_^-^ + 3 H_2_ + 2 H^+^ | 6 |
| **A9** | N_2_ + 6 H_2_O → 2 NO_3_^-^ + 5 H_2_ + 2 H^+^ | 10 |
| **A10** | NO_2_^-^ + H_2_O → NO_3_^-^ + H_2_ | 2 |
| **A11** | H_2_S + 4 H_2_O → SO_4_^2-^ + 4 H_2_ + 2 H^+^ | 8 |
| **A12** | S + 4 H_2_O → SO_4_^2-^ + 3 H_2_ + 2 H^+^ | 6 |
| **A13** | FeS_2 Pyrite_ + 8 H_2_O → 2 SO_4_^2-^ + Fe^2+^ + 7 H_2_ + 2 H^+^ | 14 |
| **A14** | 3 FeS_2 Pyrite_ + 4 H_2_O → 6 S + Fe_3_O_4 Magnetite_ + 4 H_2_ | 8 |
| **A15** | 3 FeS_2 Pyrite_ + 28 H_2_O → 6 SO_4_^2-^ + Fe_3_O_4 Magnetite_ + 22 H_2_ + 12 H^+^ | 44 |
| **A16** | 3 Fe^2+^ + 4 H_2_O → Fe_3_O_4 Magnetite_ + H_2_ + 6 H^+^ | 2 |
| **A17** | 2 Fe^2+^ + 3 H_2_O → Fe_2_O_3 Hematite_ + H_2_ + 4 H^+^ | 2 |
| **A18** | 2 Fe^2+^ + 4 H_2_O → 2 FeOOH _Goethite_ + H_2_ + 4 H^+^ | 2 |
| **A19** | 2 Fe^2+^ + 4 H_2_O → 2 FeOOH _Ferrihydrite_ + H_2_ + 4 H^+^ | 2 |
| **A20** | 2 Fe_3_O_4 Magnetite_ + H_2_O → 3 Fe_2_O_3 Hematite_ + H_2_ | 2 |
| **A21** | 2 Fe_3_O_4 Magnetite_ + 4 H_2_O → 6 FeOOH _Goethite_ + H_2_ | 2 |
| **A22** | 2 Fe_3_O_4 Magnetite_ + 4 H_2_O → 6 FeOOH _Ferrihydrite_ + H_2_ | 2 |
| **A23** | Mn^2+^ + 2 H_2_O → MnO_2 Pyrolusite_ + H_2_ + 2 H^+^ | 2 |
| **A24** | H_3_AsO_3_ + H_2_O → H_2_AsO_4_^-^ + H_2_ + H^+^ | 2 |
|  | **Oxidation of H, C, N, S, Fe, Mn, and As Species (with O_2_ as Oxidant)** |  |
| **B1** | 2H_2_ + O_2_ →2 H_2_O | 4 |
| **B2** | 2CH_4_ + 3O_2_ → 2CO + 4H_2_O | 12 |
| **B3** | CH_4_ + 2O_2_ → CO_2_ + 2H_2_O | 8 |
| **B4** | CH_4_ + 2O_2_ → HCO_3_^-^ + H_2_O + H^+^ | 8 |
| **B5** | 2 CO + O_2_ → 2 CO_2_ | 4 |
| **B6** | 2 CO + O_2_ + 2 H_2_O → 2 HCO_3_^-^ + 2 H^+^ | 4 |
| **B7** | 4 NH_4_^+^ + 3 O_2_ → 2 N_2_ + 4 H^+^ + 6 H_2_O | 12 |
| **B8** | 2 NH_4_^+^ + 3 O_2_ → 2 NO_2_^-^ + 4 H^+^ + 2 H_2_O | 12 |
| **B9** | NH_4_^+^ + 2 O_2_ → NO_3_^-^ + 2 H^+^ + H_2_O | 8 |
| **B10** | 2 N_2_ + 3 O_2_ + 2 H_2_O → 4 NO_2_^-^ + 4 H^+^ | 12 |
| **B11** | 2 N_2_ + 5 O_2_ + 2 H_2_O → 4 NO_3_^-^ + 4 H^+^ | 20 |
| **B12** | 2 NO_2_^-^ + O_2_ → 2 NO_3_^-^ | 4 |
| **B13** | 4 H_2_S + O_2_ + 2 Fe^2+^ → 2 FeS_2 Pyrite_ + 4 H^+^ + 2 H_2_O | 4 |
| **B14** | 2 H_2_S + O_2_ → 2 S + 2 H_2_O | 4 |
| **B15** | H_2_S + 2 O_2_ → SO_4_^2-^ + 2 H^+^ | 8 |
| **B16** | 2 S + 3 O_2_ + 2 H_2_O → 2 SO_4_^2-^ + 4 H^+^ | 12 |
| **B17** | 2 FeS_2 Pyrite_ + O_2_ + 4 H^+^ → 4 S + 2 Fe^2+^ + 2 H_2_O | 4 |
| **B18** | 2 FeS_2 Pyrite_ + 7 O_2_ + 2 H_2_O → 4 SO_4_^2-^ + 2 Fe^2+^ + 4 H^+^ | 28 |
| **B19** | 3 FeS_2 Pyrite_ + 2 O_2_ → 6 S + Fe_3_O_4 Magnetite_ | 6 |
| **B20** | 3 FeS_2 Pyrite_ + 11 O_2_ + 6 H_2_O → 6 SO_4_^2-^ + Fe_3_O_4 Magnetite_ + 12 H^+^ | 42 |
| **B21** | 6 Fe^2+^ + O_2_ + 6 H_2_O → 2 Fe_3_O_4 Magnetite_ + 12 H^+^ | 4 |
| **B22** | 4 Fe^2+^ + O_2_ + 4 H_2_O → 2 Fe_2_O_3 Hematite_ + 8 H^+^ | 4 |
| **B23** | 4 Fe^2+^ + O_2_ + 6 H_2_O → 4 FeOOH _Goethite_ + 8 H^+^ | 4 |
| **B24** | 4 Fe^2+^ + O_2_ + 6 H_2_O → 4 FeOOH _Ferrihydrite_ + 8 H^+^ | 4 |
| **B25** | 4 Fe_3_O_4 Magnetite_ + O_2_ → 6 Fe_2_O_3 Hematite_ | 4 |
| **B26** | 4 Fe_3_O_4 Magnetite_ + O_2_ + 6 H_2_O → 12 FeOOH _Goethite_ | 4 |
| **B27** | 4 Fe_3_O_4 Magnetite_ + O_2_ + 6 H_2_O → 12 FeOOH _Ferrihydrite_ | 4 |
| **B28** | 2 Mn^2+^ + O_2_ + 2 H_2_O → 2 MnO_2 Pyrolusite_ + 4 H^+^ | 4 |
| **B29** | 2 H_3_AsO_3_ + O_2_ → 2 H_2_AsO_4_^-^ + 2 H^+^ | 4 |
|  | **Reduction of CO** |  |
| **C1** | 2 CO + 4 H_2_O → 3 O_2_ + 2 CH_4_ | 12 |
| **C2** | 4 CO + 2 H_2_O → 3 CO_2_ + CH_4_ | 24 |
| **C3** | 3H_2_ + CO → CH_4_ + H_2_O | 6 |
| **C4** | 2 NH_4_^+^ + CO → N_2_ + CH_4_ + 2 H^+^ + H_2_O | 6 |
| **C5** | NH_4_^+^ + CO + H_2_O → NO_2_^-^ + CH_4_ + 2 H^+^ | 6 |
| **C6** | 3 NH_4_^+^ + 4 CO + 5 H_2_O → 3 NO_3_^-^ + 4 CH_4_ + 6 H^+^ | 24 |
| **C7** | N_2_ + CO + 3 H_2_O → 2 NO_2_^-^ + CH_4_ + 2 H^+^ | 6 |
| **C8** | 3 N_2_ + 5 CO + 13 H_2_O → 6 NO_3_^-^ + 5 CH_4_ + 6 H^+^ | 30 |
| **C9** | 3 NO_2_^-^ + CO + 2 H_2_O → 3 NO_3_^-^ + CH_4_ | 6 |
| **C10** | 6 H_2_S + CO + 3 Fe^2+^ → 3 FeS_2 Pyrite_ + CH_4_ + 6 H^+^ + H_2_O | 6 |
| **C11** | 3 H_2_S + CO → 3 S + CH_4_ + H_2_O | 6 |
| **C12** | 3 H_2_S + 4 CO + 8 H_2_O → 3 SO_4_^2-^ + 4 CH_4_ + 6 H^+^ | 24 |
| **C13** | S + CO + 3 H_2_O → SO_4_^2-^ + CH_4_ + 2 H^+^ | 6 |
| **C14** | 3 FeS_2 Pyrite_ + CO + 6 H^+^ → 6 S + 3 Fe^2+^ + CH_4_ + H_2_O | 6 |
| **C15** | 3 FeS_2 Pyrite_ + 7 CO + 17 H_2_O → 6 SO_4_^2-^ + 3 Fe^2+^ + 7 CH_4_ + 6 H^+^ | 42 |
| **C16** | 9 FeS_2 Pyrite_ + 4 CO + 8 H_2_O → 18 S + 3 Fe_3_O_4 Magnetite_ + 4 CH_4_ | 24 |
| **C17** | 9 FeS_2 Pyrite_ + 22 CO + 62 H_2_O → 18 SO_4_^2-^ + 3 Fe_3_O_4 Magnetite_ + 22 CH_4_ + 36 H^+^ | 132 |
| **C18** | 9 Fe^2+^ + CO + 11 H_2_O → 3 Fe_3_O_4 Magnetite_ + CH_4_ + 18 H^+^ | 6 |
| **C19** | 6 Fe^2+^ + CO + 8 H_2_O → 3 Fe_2_O_3 Hematite_ + CH_4_ + 12 H^+^ | 6 |
| **C20** | 6 Fe^2+^ + CO + 11 H_2_O → 6 FeOOH _Goethite_ + CH_4_ + 12 H^+^ | 6 |
| **C21** | 6 Fe^2+^ + CO + 11 H_2_O → 6 FeOOH _Ferrihydrite_ + CH_4_ + 12 H^+^ | 6 |
| **C22** | 6 Fe_3_O_4 Magnetite_ + CO + 2 H_2_O → 9 Fe_2_O_3 Hematite_ + CH_4_ | 6 |
| **C23** | 6 Fe_3_O_4 Magnetite_ + CO + 11 H_2_O → 18 FeOOH _Goethite_ + CH_4_ | 6 |
| **C24** | 6 Fe_3_O_4 Magnetite_ + CO + 11 H_2_O → 18 FeOOH _Ferrihydrite_ + CH_4_ | 6 |
| **C25** | 3 Mn^2+^ + CO + 5 H_2_O → 3 MnO_2 Pyrolusite_ + CH_4_ + 6 H^+^ | 6 |
| **C26** | 3 H_3_AsO_3_ + CO + 2 H_2_O → 3 H_2_AsO_4_^-^ + CH_4_ + 3 H^+^ | 6 |
|  | **Reduction of CO_2_** |  |
| **D1** | 2 CO_2_ → 2 CO + O_2_ | 4 |
| **D2** | CO_2_ + 2 H_2_O → CH_4_ + 2 O_2_ | 8 |
| **D3** | 4H_2_ + CO_2_ → CH_4_ + 2H_2_O | 8 |
| **D4** | CH_4_ + 3CO_2_ → 4CO + 2H_2_O | 6 |
| **D5** | 8 NH_4_^+^ + 3 CO_2_ → 4 N_2_ + 3 CH_4_ + 8 H^+^ + 6 H_2_O | 24 |
| **D6** | 4 NH_4_^+^ + 3 CO_2_ + 2 H_2_O → 4 NO_2_^-^ + 3 CH_4_ + 8 H^+^ | 24 |
| **D7** | NH_4_^+^ + CO_2_ + H_2_O → NO_3_^-^ + CH_4_ + 2 H^+^ | 8 |
| **D8** | 2 NH_4_^+^ + 3 CO_2_ → N_2_ + 3 CO + 2 H^+^ + 3 H_2_O | 6 |
| **D9** | NH_4_^+^ + 3 CO_2_ → NO_2_^-^ + 3 CO + 2 H^+^ + H_2_O | 6 |
| **D10** | NH_4_^+^ + 4 CO_2_ → NO_3_^-^ + 4 CO + 2 H^+^ + H_2_O | 8 |
| **D11** | 4 N_2_ + 3 CO_2_ + 10 H_2_O → 8 NO_2_^-^ + 3 CH_4_ + 8 H^+^ | 24 |
| **D12** | 4 N_2_ + 5 CO_2_ + 14 H_2_O → 8 NO_3_^-^ + 5 CH_4_ + 8 H^+^ | 40 |
| **D13** | N_2_ + 3 CO_2_ + H_2_O → 2 NO_2_^-^ + 3 CO + 2 H^+^ | 6 |
| **D14** | N_2_ + 5 CO_2_ + H_2_O → 2 NO_3_^-^ + 5 CO + 2 H^+^ | 10 |
| **D15** | 4 NO_2_^-^ + CO_2_ + 2 H_2_O → 4 NO_3_^-^ + CH_4_ | 8 |
| **D16** | NO_2_^-^ + CO_2_ → NO_3_^-^ + CO | 2 |
| **D17** | 8 H_2_S + CO_2_ + 4 Fe^2+^ → 4 FeS_2 Pyrite_ + CH_4_ + 8 H^+^ + 2 H_2_O | 8 |
| **D18** | 4 H_2_S + CO_2_ → 4 S + CH_4_ + 2 H_2_O | 8 |
| **D19** | H_2_S + CO_2_ + 2 H_2_O → SO_4_^2-^ + CH_4_ + 2 H^+^ | 8 |
| **D20** | 2 H_2_S + CO_2_ + Fe^2+^ → FeS_2 Pyrite_ + CO + 2 H^+^ + H_2_O | 2 |
| **D21** | H_2_S + CO_2_ → S + CO + H_2_O | 2 |
| **D22** | H_2_S + 4 CO_2_ → SO_4_^2-^ + 4 CO + 2 H^+^ | 8 |
| **D23** | 4 S + 3 CO_2_ + 10 H_2_O → 4 SO_4_^2-^ + 3 CH_4_ + 8 H^+^ | 24 |
| **D24** | S + 3 CO_2_ + H_2_O → SO_4_^2-^ + 3 CO + 2 H^+^ | 6 |
| **D25** | 4 FeS_2 Pyrite_ + CO_2_ + 8 H^+^ → 8 S + 4 Fe^2+^ + CH_4_ + 2 H_2_O | 8 |
| **D26** | 4 FeS_2 Pyrite_ + 7 CO_2_ + 18 H_2_O → 8 SO_4_^2-^ + 4 Fe^2+^ + 7 CH_4_ + 8 H^+^ | 56 |
| **D27** | 3 FeS_2 Pyrite_ + CO_2_ + 2 H_2_O → 6 S + Fe_3_O_4 Magnetite_ + CH_4_ | 6 |
| **D28** | 6 FeS_2 Pyrite_ + 11 CO_2_ + 34 H_2_O → 12 SO_4_^2-^ + 2 Fe_3_O_4 Magnetite_ + 11 CH_4_ + 24 H^+^ | 88 |
| **D29** | FeS_2 Pyrite_ + CO_2_ + 2 H^+^ → 2 S + Fe^2+^ + CO + H_2_O | 2 |
| **D30** | FeS_2 Pyrite_ + 7 CO_2_ + H_2_O → 2 SO_4_^2-^ + Fe^2+^ + 7 CO + 2 H^+^ | 14 |
| **D31** | 3 FeS_2 Pyrite_ + 4 CO_2_ → 6 S + Fe_3_O_4 Magnetite_ + 4 CO | 8 |
| **D32** | 3 FeS_2 Pyrite_ + 22 CO_2_ + 6 H_2_O → 6 SO_4_^2-^ + Fe_3_O_4 Magnetite_ + 22 CO + 12 H^+^ | 44 |
| **D33** | 12 Fe^2+^ + CO_2_ + 14 H_2_O → 4 Fe_3_O_4 Magnetite_ + CH_4_ + 24 H^+^ | 8 |
| **D34** | 8 Fe^2+^ + CO_2_ + 10 H_2_O → 4 Fe_2_O_3 Hematite_ + CH_4_ + 16 H^+^ | 8 |
| **D35** | 8 Fe^2+^ + CO_2_ + 14 H_2_O → 8 FeOOH _Goethite_ + CH_4_ + 16 H^+^ | 8 |
| **D36** | 8 Fe^2+^ + CO_2_ + 14 H_2_O → 8 FeOOH _Ferrihydrite_ + CH_4_ + 16 H^+^ | 8 |
| **D37** | 3 Fe^2+^ + CO_2_ + 3 H_2_O → Fe_3_O_4 Magnetite_ + CO + 6 H^+^ | 2 |
| **D38** | 2 Fe^2+^ + CO_2_ + 2 H_2_O → Fe_2_O_3 Hematite_ + CO + 4 H^+^ | 2 |
| **D39** | 2 Fe^2+^ + CO_2_ + 3 H_2_O → 2 FeOOH _Goethite_ + CO + 4 H^+^ | 2 |
| **D40** | 2 Fe^2+^ + CO_2_ + 3 H_2_O → 2 FeOOH _Ferrihydrite_ + CO + 4 H^+^ | 2 |
| **D41** | 8 Fe_3_O_4 Magnetite_ + CO_2_ + 2 H_2_O → 12 Fe_2_O_3 Hematite_ + CH_4_ | 8 |
| **D42** | 8 Fe_3_O_4 Magnetite_ + CO_2_ + 14 H_2_O → 24 FeOOH _Goethite_ + CH_4_ | 8 |
| **D43** | 8 Fe_3_O_4 Magnetite_ + CO_2_ + 14 H_2_O → 24 FeOOH _Ferrihydrite_ + CH_4_ | 8 |
| **D44** | 2 Fe_3_O_4 Magnetite_ + CO_2_ → 3 Fe_2_O_3 Hematite_ + CO | 2 |
| **D45** | 2 Fe_3_O_4 Magnetite_ + CO_2_ + 3 H_2_O → 6 FeOOH _Goethite_ + CO | 2 |
| **D46** | 2 Fe_3_O_4 Magnetite_ + CO_2_ + 3 H_2_O → 6 FeOOH _Ferrihydrite_ + CO | 2 |
| **D47** | 4 Mn^2+^ + CO_2_ + 6 H_2_O → 4 MnO_2 Pyrolusite_ + CH_4_ + 8 H^+^ | 8 |
| **D48** | Mn^2+^ + CO_2_ + H_2_O → MnO_2 Pyrolusite_ + CO + 2 H^+^ | 2 |
| **D49** | 4 H_3_AsO_3_ + CO_2_ + 2 H_2_O → 4 H_2_AsO_4_^-^ + CH_4_ + 4 H^+^ | 8 |
| **D50** | H_3_AsO_3_ + CO_2_ → H_2_AsO_4_^-^ + CO + H^+^ | 2 |
|  | **Reduction of HCO_3_^-^** |  |
| **E1** | 2 HCO_3_^-^ + 2 H^+^ → 2 CO + O_2_ + 2 H_2_O | 4 |
| **E2** | HCO_3_^-^ + H_2_O + H^+^ → CH_4_ + 2 O_2_ | 8 |
| **E3** | 4H_2_ + HCO_3_^-^ + H^+^→ CH_4_ + 3H_2_O | 8 |
| **E4** | CH_4_ + 3HCO_3_^-^ + 3H^+^→ 4CO + 5H_2_O | 6 |
| **E5** | 8 NH_4_^+^ + 3 HCO_3_^-^ → 4 N_2_ + 3 CH_4_ + 5 H^+^ + 9 H_2_O | 24 |
| **E6** | 4 NH_4_^+^ + 3 HCO_3_^-^ → 4 NO_2_^-^ + 3 CH_4_ + 5 H^+^ + H_2_O | 24 |
| **E7** | NH_4_^+^ + HCO_3_^-^ → NO_3_^-^ + CH_4_ + H^+^ | 8 |
| **E8** | 2 NH_4_^+^ + 3 HCO_3_^-^ + H^+^ → N_2_ + 3 CO + 6 H_2_O | 6 |
| **E9** | NH_4_^+^ + 3 HCO_3_^-^ + H^+^ → NO_2_^-^ + 3 CO + 4 H_2_O | 6 |
| **E10** | NH_4_^+^ + 4 HCO_3_^-^ + 2 H^+^ → NO_3_^-^ + 4 CO + 5 H_2_O | 8 |
| **E11** | 4 N_2_ + 3 HCO_3_^-^ + 7 H_2_O → 8 NO_2_^-^ + 3 CH_4_ + 5 H^+^ | 24 |
| **E12** | 4 N_2_ + 5 HCO_3_^-^ + 9 H_2_O → 8 NO_3_^-^ + 5 CH_4_ + 3 H^+^ | 40 |
| **E13** | N_2_ + 3 HCO_3_^-^ + H^+^ → 2 NO_2_^-^ + 3 CO + 2 H_2_O | 6 |
| **E14** | N_2_ + 5 HCO_3_^-^ + 3 H^+^ → 2 NO_3_^-^ + 5 CO + 4 H_2_O | 10 |
| **E15** | 4 NO_2_^-^ + HCO_3_^-^ + H^+^ + H_2_O → 4 NO_3_^-^ + CH_4_ | 8 |
| **E16** | NO_2_^-^ + HCO_3_^-^ + H^+^ → NO_3_^-^ + CO + H_2_O | 2 |
| **E17** | 8 H_2_S + HCO_3_^-^ + 4 Fe^2+^ → 4 FeS_2 Pyrite_ + CH_4_ + 7 H^+^ + 3 H_2_O | 8 |
| **E18** | 4 H_2_S + HCO_3_^-^ + H^+^ → 4 S + CH_4_ + 3 H_2_O | 8 |
| **E19** | H_2_S + HCO_3_^-^ + H_2_O → SO_4_^2-^ + CH_4_ + H^+^ | 8 |
| **E20** | 2 H_2_S + HCO_3_^-^ + Fe^2+^ → FeS_2 Pyrite_ + CO + H^+^ + 2 H_2_O | 2 |
| **E21** | H_2_S + HCO_3_^-^ + H^+^ → S + CO + 2 H_2_O | 2 |
| **E22** | H_2_S + 4 HCO_3_^-^ + 2 H^+^ → SO_4_^2-^ + 4 CO + 4 H_2_O | 8 |
| **E23** | 4 S + 3 HCO_3_^-^ + 7 H_2_O → 4 SO_4_^2-^ + 3 CH_4_ + 5 H^+^ | 24 |
| **E24** | S + 3 HCO_3_^-^ + H^+^ → SO_4_^2-^ + 3 CO + 2 H_2_O | 6 |
| **E25** | 4 FeS_2 Pyrite_ + HCO_3_^-^ + 9 H^+^ → 8 S + 4 Fe^2+^ + CH_4_ + 3 H_2_O | 8 |
| **E26** | 4 FeS_2 Pyrite_ + 7 HCO_3_^-^ + 11 H_2_O → 8 SO_4_^2-^ + 4 Fe^2+^ + 7 CH_4_ + H^+^ | 56 |
| **E27** | 3 FeS_2 Pyrite_ + HCO_3_^-^ + H^+^ + H_2_O → 6 S + Fe_3_O_4 Magnetite_ + CH_4_ | 6 |
| **E28** | 6 FeS_2 Pyrite_ + 11 HCO_3_^-^ + 23 H_2_O → 12 SO_4_^2-^ + 2 Fe_3_O_4 Magnetite_ + 11 CH_4_ + 13 H^+^ | 88 |
| **E29** | FeS_2 Pyrite_ + HCO_3_^-^ + 3 H^+^ → 2 S + Fe^2+^ + CO + 2 H_2_O | 2 |
| **E30** | FeS_2 Pyrite_ + 7 HCO_3_^-^ + 5 H^+^ → 2 SO_4_^2-^ + Fe^2+^ + 7 CO + 6 H_2_O | 14 |
| **E31** | 3 FeS_2 Pyrite_ + 4 HCO_3_^-^ + 4 H^+^ → 6 S + Fe_3_O_4 Magnetite_ + 4 CO + 4 H_2_O | 8 |
| **E32** | 3 FeS_2 Pyrite_ + 22 HCO_3_^-^ + 10 H^+^ → 6 SO_4_^2-^ + Fe_3_O_4 Magnetite_ + 22 CO + 16 H_2_O | 44 |
| **E33** | 12 Fe^2+^ + HCO_3_^-^ + 13 H_2_O → 4 Fe_3_O_4 Magnetite_ + CH_4_ + 23 H^+^ | 8 |
| **E34** | 8 Fe^2+^ + HCO_3_^-^ + 9 H_2_O → 4 Fe_2_O_3 Hematite_ + CH_4_ + 15 H^+^ | 8 |
| **E35** | 8 Fe^2+^ + HCO_3_^-^ + 13 H_2_O → 8 FeOOH _Goethite_ + CH_4_ + 15 H^+^ | 8 |
| **E36** | 8 Fe^2+^ + HCO_3_^-^ + 13 H_2_O → 8 FeOOH _Ferrihydrite_ + CH_4_ + 15 H^+^ | 8 |
| **E37** | 3 Fe^2+^ + HCO_3_^-^ + 2 H_2_O → Fe_3_O_4 Magnetite_ + CO + 5 H^+^ | 2 |
| **E38** | 2 Fe^2+^ + HCO_3_^-^ + H_2_O → Fe_2_O_3 Hematite_ + CO + 3 H^+^ | 2 |
| **E39** | 2 Fe^2+^ + HCO_3_^-^ + 2 H_2_O → 2 FeOOH _Goethite_ + CO + 3 H^+^ | 2 |
| **E40** | 2 Fe^2+^ + HCO_3_^-^ + 2 H_2_O → 2 FeOOH _Ferrihydrite_ + CO + 3 H^+^ | 2 |
| **E41** | 8 Fe_3_O_4 Magnetite_ + HCO_3_^-^ + H^+^ + H_2_O → 12 Fe_2_O_3 Hematite_ + CH_4_ | 8 |
| **E42** | 8 Fe_3_O_4 Magnetite_ + HCO_3_^-^ + H^+^ + 13 H_2_O → 24 FeOOH _Goethite_ + CH_4_ | 8 |
| **E43** | 8 Fe_3_O_4 Magnetite_ + HCO_3_^-^ + H^+^ + 13 H_2_O → 24 FeOOH _Ferrihydrite_ + CH_4_ | 8 |
| **E44** | 2 Fe_3_O_4 Magnetite_ + HCO_3_^-^ + H^+^ → 3 Fe_2_O_3 Hematite_ + CO + H_2_O | 2 |
| **E45** | 2 Fe_3_O_4 Magnetite_ + HCO_3_^-^ + H^+^ + 2 H_2_O → 6 FeOOH _Goethite_ + CO | 2 |
| **E46** | 2 Fe_3_O_4 Magnetite_ + HCO_3_^-^ + H^+^ + 2 H_2_O → 6 FeOOH _Ferrihydrite_ + CO | 2 |
| **E47** | 4 Mn^2+^ + HCO_3_^-^ + 5 H_2_O → 4 MnO_2 Pyrolusite_ + CH_4_ + 7 H^+^ | 8 |
| **E48** | Mn^2+^ + HCO_3_^-^ → MnO_2 Pyrolusite_ + CO + H^+^ | 2 |
| **E49** | 4 H_3_AsO_3_ + HCO_3_^-^ + H_2_O → 4 H_2_AsO_4_^-^ + CH_4_ + 3 H^+^ | 8 |
| **E50** | H_3_AsO_3_ + HCO_3_^-^ → H_2_AsO_4_^-^ + CO + H_2_O | 2 |
|  | **Oxidation of NH_4_^+^** |  |
| **F1** | 2 NH_4_^+^ → N_2_ + 3 H_2_ + 2 H^+^ | 6 |
|  | **Reduction of N_2_** |  |
| **G1** | 2 N_2_ + 6 H_2_O + 4 H^+^ → 4 NH_4_^+^ + 3 O_2_ | 12 |
| **G2** | 3H_2_ + N_2_ + 2H^+^ → 2NH_4_^+^ | 6 |
| **G3** | CH_4_ + N_2_ + 2H^+^ + H_2_O → 2NH_4_^+^ + CO | 6 |
| **G4** | 3CH_4_ + 4N_2_ + 8H^+^ + 6H_2_O → 8NH_4_^+^ + 3CO_2_ | 24 |
| **G5** | 3CH_4_ + 4N_2_ + 5H^+^ + 9H_2_O → 8NH_4_^+^ + 3HCO_3_^-^ | 24 |
| **G6** | 3 CO + N_2_ + 2 H^+^ + 3 H_2_O → 2 NH_4_^+^ + 3 CO_2_ | 6 |
| **G7** | 3 CO + N_2_ + 6 H_2_O → 2 NH_4_^+^ + 3 HCO_3_^-^ + H^+^ | 6 |
| **G8** | 6 H_2_S + N_2_ + 3 Fe^2+^ → 3 FeS_2 Pyrite_ + 2 NH_4_^+^ + 4 H^+^ | 6 |
| **G9** | 3 H_2_S + N_2_ + 2 H^+^ → 3 S + 2 NH_4_^+^ | 6 |
| **G10** | 3 H_2_S + 4 N_2_ + 2 H^+^ + 12 H_2_O → 3 SO_4_^2-^ + 8 NH_4_^+^ | 24 |
| **G11** | S + N_2_ + 4 H_2_O → SO_4_^2-^ + 2 NH_4_^+^ | 6 |
| **G12** | 3 FeS_2 Pyrite_ + N_2_ + 8 H^+^ → 6 S + 3 Fe^2+^ + 2 NH_4_^+^ | 6 |
| **G13** | 3 FeS_2 Pyrite_ + 7 N_2_ + 8 H^+^ + 24 H_2_O → 6 SO_4_^2-^ + 3 Fe^2+^ + 14 NH_4_^+^ | 42 |
| **G14** | 9 FeS_2 Pyrite_ + 4 N_2_ + 8 H^+^ + 12 H_2_O → 18 S + 3 Fe_3_O_4 Magnetite_ + 8 NH_4_^+^ | 24 |
| **G15** | 9 FeS_2 Pyrite_ + 22 N_2_ + 8 H^+^ + 84 H_2_O → 18 SO_4_^2-^ + 3 Fe_3_O_4 Magnetite_ + 44 NH_4_^+^ | 132 |
| **G16** | 9 Fe^2+^ + N_2_ + 12 H_2_O → 3 Fe_3_O_4 Magnetite_ + 2 NH_4_^+^ + 16 H^+^ | 6 |
| **G17** | 6 Fe^2+^ + N_2_ + 9 H_2_O → 3 Fe_2_O_3 Hematite_ + 2 NH_4_^+^ + 10 H^+^ | 6 |
| **G18** | 6 Fe^2+^ + N_2_ + 12 H_2_O → 6 FeOOH _Goethite_ + 2 NH_4_^+^ + 10 H^+^ | 6 |
| **G19** | 6 Fe^2+^ + N_2_ + 12 H_2_O → 6 FeOOH _Ferrihydrite_ + 2 NH_4_^+^ + 10 H^+^ | 6 |
| **G20** | 6 Fe_3_O_4 Magnetite_ + N_2_ + 2 H^+^ + 3 H_2_O → 9 Fe_2_O_3 Hematite_ + 2 NH_4_^+^ | 6 |
| **G21** | 6 Fe_3_O_4 Magnetite_ + N_2_ + 2 H^+^ + 12 H_2_O → 18 FeOOH _Goethite_ + 2 NH_4_^+^ | 6 |
| **G22** | 6 Fe_3_O_4 Magnetite_ + N_2_ + 2 H^+^ + 12 H_2_O → 18 FeOOH _Ferrihydrite_ + 2 NH_4_^+^ | 6 |
| **G23** | 3 Mn^2+^ + N_2_ + 6 H_2_O → 3 MnO_2 Pyrolusite_ + 2 NH_4_^+^ + 4 H^+^ | 6 |
| **G24** | 3 H_3_AsO_3_ + N_2_ + 3 H_2_O → 3 H_2_AsO_4_^-^ + 2 NH_4_^+^ + H^+^ | 6 |
|  | **Reduction of NO_2_^-^** |  |
| **H1** | 4 NO_2_^-^ + 4 H^+^ → 2 N_2_ + 3 O_2_ + 2 H_2_O | 12 |
| **H2** | 2 NO_2_^-^ + 4 H^+^ + 2 H_2_O → 2 NH_4_^+^ + 3 O_2_ | 12 |
| **H3** | 3H_2_ + NO_2_^-^ + 2H^+^ → NH_4_^+^ + 2H_2_O | 6 |
| **H4** | 3H_2_ + 2NO_2_^-^ + 2H^+^ → N_2_ + 4H_2_O | 6 |
| **H5** | CH_4_ + NO_2_^-^ + 2H^+^ → NH_4_^+^ + CO + H_2_O | 6 |
| **H6** | 3CH_4_ + 4NO_2_^-^ + 8H^+^ → 4NH_4_^+^ + 3CO_2_ + 2H_2_O | 24 |
| **H7** | 3CH_4_ + 4NO_2_^-^ + 5H^+^ +H_2_O→ 4NH_4_^+^ + 3HCO_3_^-^ | 24 |
| **H8** | CH_4_ + 2NO_2_^-^ + 2H^+^ → N_2_ + CO + 3H_2_O | 6 |
| **H9** | 3CH_4_ + 8NO_2_^-^ + 8H^+^ → 4N_2_ + 3CO_2_ + 10H_2_O | 24 |
| **H10** | 3CH_4_ + 8NO_2_^-^ + 5H^+^ → 4N_2_ + 3HCO_3_^-^ + 7H_2_O | 24 |
| **H11** | 3 CO + NO_2_^-^ + 2 H^+^ + H_2_O → NH_4_^+^ + 3 CO_2_ | 6 |
| **H12** | 3 CO + NO_2_^-^ + 4 H_2_O → NH_4_^+^ + 3 HCO_3_^-^ + H^+^ | 6 |
| **H13** | 3 CO + 2 NO_2_^-^ + 2 H^+^ → N_2_ + 3 CO_2_ + H_2_O | 6 |
| **H14** | 3 CO + 2 NO_2_^-^ + 2 H_2_O → N_2_ + 3 HCO_3_^-^ + H^+^ | 6 |
| **H15** | NH_4_^+^ + NO_2_^-^ → N_2_ + 2 H_2_O | 3 |
| **H16** | 6 H_2_S + NO_2_^-^ + 3 Fe^2+^ → 3 FeS_2 Pyrite_ + NH_4_^+^ + 4 H^+^ + 2 H_2_O | 6 |
| **H17** | 3 H_2_S + NO_2_^-^ + 2 H^+^ → 3 S + NH_4_^+^ + 2 H_2_O | 6 |
| **H18** | 3 H_2_S + 4 NO_2_^-^ + 2 H^+^ + 4 H_2_O → 3 SO_4_^2-^ + 4 NH_4_^+^ | 24 |
| **H19** | 6 H_2_S + 2 NO_2_^-^ + 3 Fe^2+^ → 3 FeS_2 Pyrite_ + N_2_ + 4 H^+^ + 4 H_2_O | 6 |
| **H20** | 3 H_2_S + 2 NO_2_^-^ + 2 H^+^ → 3 S + N_2_ + 4 H_2_O | 6 |
| **H21** | 3 H_2_S + 8 NO_2_^-^ + 2 H^+^ → 3 SO_4_^2-^ + 4 N_2_ + 4 H_2_O | 24 |
| **H22** | S + NO_2_^-^ + 2 H_2_O → SO_4_^2-^ + NH_4_^+^ | 6 |
| **H23** | S + 2 NO_2_^-^ → SO_4_^2-^ + N_2_ | 6 |
| **H24** | 3 FeS_2 Pyrite_ + NO_2_^-^ + 8 H^+^ → 6 S + 3 Fe^2+^ + NH_4_^+^ + 2 H_2_O | 6 |
| **H25** | 3 FeS_2 Pyrite_ + 7 NO_2_^-^ + 8 H^+^ + 10 H_2_O → 6 SO_4_^2-^ + 3 Fe^2+^ + 7 NH_4_^+^ | 42 |
| **H26** | 9 FeS_2 Pyrite_ + 4 NO_2_^-^ + 8 H^+^ + 4 H_2_O → 18 S + 3 Fe_3_O_4 Magnetite_ + 4 NH_4_^+^ | 24 |
| **H27** | 9 FeS_2 Pyrite_ + 22 NO_2_^-^ + 8 H^+^ + 40 H_2_O → 18 SO_4_^2-^ + 3 Fe_3_O_4 Magnetite_ + 22 NH_4_^+^ | 132 |
| **H28** | 3 FeS_2 Pyrite_ + 2 NO_2_^-^ + 8 H^+^ → 6 S + 3 Fe^2+^ + N_2_ + 4 H_2_O | 6 |
| **H29** | 3 FeS_2 Pyrite_ + 14 NO_2_^-^ + 8 H^+^ → 6 SO_4_^2-^ + 3 Fe^2+^ + 7 N_2_ + 4 H_2_O | 42 |
| **H30** | 9 FeS_2 Pyrite_ + 8 NO_2_^-^ + 8 H^+^ → 18 S + 3 Fe_3_O_4 Magnetite_ + 4 N_2_ + 4 H_2_O | 24 |
| **H31** | 9 FeS_2 Pyrite_ + 44 NO_2_^-^ + 8 H^+^ → 18 SO_4_^2-^ + 3 Fe_3_O_4 Magnetite_ + 22 N_2_ + 4 H_2_O | 132 |
| **H32** | 9 Fe^2+^ + NO_2_^-^ + 10 H_2_O → 3 Fe_3_O_4 Magnetite_ + NH_4_^+^ + 16 H^+^ | 6 |
| **H33** | 6 Fe^2+^ + NO_2_^-^ + 7 H_2_O → 3 Fe_2_O_3 Hematite_ + NH_4_^+^ + 10 H^+^ | 6 |
| **H34** | 6 Fe^2+^ + NO_2_^-^ + 10 H_2_O → 6 FeOOH _Goethite_ + NH_4_^+^ + 10 H^+^ | 6 |
| **H35** | 6 Fe^2+^ + NO_2_^-^ + 10 H_2_O → 6 FeOOH _Ferrihydrite_ + NH_4_^+^ + 10 H^+^ | 6 |
| **H36** | 9 Fe^2+^ + 2 NO_2_^-^ + 8 H_2_O → 3 Fe_3_O_4 Magnetite_ + N_2_ + 16 H^+^ | 6 |
| **H37** | 6 Fe^2+^ + 2 NO_2_^-^ + 5 H_2_O → 3 Fe_2_O_3 Hematite_ + N_2_ + 10 H^+^ | 6 |
| **H38** | 6 Fe^2+^ + 2 NO_2_^-^ + 8 H_2_O → 6 FeOOH _Goethite_ + N_2_ + 10 H^+^ | 6 |
| **H39** | 6 Fe^2+^ + 2 NO_2_^-^ + 8 H_2_O → 6 FeOOH _Ferrihydrite_ + N_2_ + 10 H^+^ | 6 |
| **H40** | 6 Fe_3_O_4 Magnetite_ + NO_2_^-^ + 2 H^+^ + H_2_O → 9 Fe_2_O_3 Hematite_ + NH_4_^+^ | 6 |
| **H41** | 6 Fe_3_O_4 Magnetite_ + NO_2_^-^ + 2 H^+^ + 10 H_2_O → 18 FeOOH _Goethite_ + NH_4_^+^ | 6 |
| **H42** | 6 Fe_3_O_4 Magnetite_ + NO_2_^-^ + 2 H^+^ + 10 H_2_O → 18 FeOOH _Ferrihydrite_ + NH_4_^+^ | 6 |
| **H43** | 6 Fe_3_O_4 Magnetite_ + 2 NO_2_^-^ + 2 H^+^ → 9 Fe_2_O_3 Hematite_ + N_2_ + H_2_O | 6 |
| **H44** | 6 Fe_3_O_4 Magnetite_ + 2 NO_2_^-^ + 2 H^+^ + 8 H_2_O → 18 FeOOH _Goethite_ + N_2_ | 6 |
| **H45** | 6 Fe_3_O_4 Magnetite_ + 2 NO_2_^-^ + 2 H^+^ + 8 H_2_O → 18 FeOOH _Ferrihydrite_ + N_2_ | 6 |
| **H46** | 3 Mn^2+^ + NO_2_^-^ + 4 H_2_O → 3 MnO_2 Pyrolusite_ + NH_4_^+^ + 4 H^+^ | 6 |
| **H47** | 3 Mn^2+^ + 2 NO_2_^-^ + 2 H_2_O → 3 MnO_2 Pyrolusite_ + N_2_ + 4 H^+^ | 6 |
| **H48** | 3 H_3_AsO_3_ + NO_2_^-^ + H_2_O → 3 H_2_AsO_4_^-^ + NH_4_^+^ + H^+^ | 6 |
| **H49** | 3 H_3_AsO_3_ + 2 NO_2_^-^ → 3 H_2_AsO_4_^-^ + N_2_ + H^+^ + H_2_O | 6 |
|  | **Reduction of NO_3_^-^** |  |
| **I1** | 4 NO_3_^-^ + 4 H^+^ → 2 N_2_ + 5 O_2_ + 2 H_2_O | 20 |
| **I2** | 2 NO_3_^-^ → 2 NO_2_^-^ + O_2_ | 4 |
| **I3** | NO_3_^-^ + 2 H^+^ + H_2_O → NH_4_^+^ + 2 O_2_ | 8 |
| **I4** | 4H_2_ + NO_3_^-^ + 2H^+^ → NH_4_^+^ + 3H_2_O | 8 |
| **I5** | 5H_2_ + 2NO_3_^-^ + 2H^+^ → N_2_ + 6H_2_O | 10 |
| **I6** | H_2_ + NO_3_^-^  → NO_2_^-^ + H_2_O | 2 |
| **I7** | 4CH_4_ + 3NO_3_^-^ + 6H^+^ → 3NH^+^ + 4CO + 5H_2_O | 24 |
| **I8** | CH_4_ + NO_3_^-^ + 2H^+^ → NH_4_^+^ + CO_2_ +H_2_O | 8 |
| **I9** | CH_4_ + NO_3_^-^ + H^+^ → NH_4_^+^ + HCO_3_^-^ | 8 |
| **I10** | 5CH_4_ + 6NO_3_^-^ + 6H^+^ → 3N_2_ + 5CO + 13H_2_O | 30 |
| **I11** | 5CH_4_ + 8NO_3_^-^ + 8H^+^ → 4N_2_ + 5CO_2_ + 14H_2_O | 40 |
| **I12** | 5CH_4_ + 8NO_3_^-^ + 3H^+^ → 4N_2_ + 5HCO_3_^-^ + 9H_2_O | 40 |
| **I13** | CH_4_ + 3NO_3_^-^ → 3NO_2_^-^ + CO + 2H_2_O | 6 |
| **I14** | CH_4_ + 4NO_3_^-^ → 4NO_2_^-^ + CO_2_ + 2H_2_O | 8 |
| **I15** | CH_4_ + 4NO_3_^-^ → 4NO_2_^-^ + HCO_3_^-^ + H_2_O + H^+^ | 8 |
| **I16** | 4 CO + NO_3_^-^ + 2 H^+^ + H_2_O → NH_4_^+^ + 4 CO_2_ | 8 |
| **I17** | 4 CO + NO_3_^-^ + 5 H_2_O → NH_4_^+^ + 4 HCO_3_^-^ + 2 H^+^ | 8 |
| **I18** | 5 CO + 2 NO_3_^-^ + 2 H^+^ → N_2_ + 5 CO_2_ + H_2_O | 10 |
| **I19** | 5 CO + 2 NO_3_^-^ + 4 H_2_O → N_2_ + 5 HCO_3_^-^ + 3 H^+^ | 10 |
| **I20** | CO + NO_3_^-^ → NO_2_^-^ + CO_2_ | 2 |
| **I21** | CO + NO_3_^-^ + H_2_O → NO_2_^-^ + HCO_3_^-^ + H^+^ | 2 |
| **I22** | 5 NH_4_^+^ + 3 NO_3_^-^ → 4 N_2_ + 2 H^+^ + 9 H_2_O | 15 |
| **I23** | NH_4_^+^ + 3 NO_3_^-^ → 4 NO_2_^-^ + 2 H^+^ + H_2_O | 6 |
| **I24** | N_2_ + 3 NO_3_^-^ + H_2_O → 5 NO_2_^-^ + 2 H^+^ | 6 |
| **I25** | 8 H_2_S + NO_3_^-^ + 4 Fe^2+^ → 4 FeS_2 Pyrite_ + NH_4_^+^ + 6 H^+^ + 3 H_2_O | 8 |
| **I26** | 4 H_2_S + NO_3_^-^ + 2 H^+^ → 4 S + NH_4_^+^ + 3 H_2_O | 8 |
| **I27** | H_2_S + NO_3_^-^ + H_2_O → SO_4_^2-^ + NH_4_^+^ | 8 |
| **I28** | 10 H_2_S + 2 NO_3_^-^ + 5 Fe^2+^ → 5 FeS_2 Pyrite_ + N_2_ + 8 H^+^ + 6 H_2_O | 10 |
| **I29** | 5 H_2_S + 2 NO_3_^-^ + 2 H^+^ → 5 S + N_2_ + 6 H_2_O | 10 |
| **I30** | 5 H_2_S + 8 NO_3_^-^ → 5 SO_4_^2-^ + 4 N_2_ + 2 H^+^ + 4 H_2_O | 40 |
| **I31** | 2 H_2_S + NO_3_^-^ + Fe^2+^ → FeS_2 Pyrite_ + NO_2_^-^ + 2 H^+^ + H_2_O | 2 |
| **I32** | H_2_S + NO_3_^-^ → S + NO_2_^-^ + H_2_O | 2 |
| **I33** | H_2_S + 4 NO_3_^-^ → SO_4_^2-^ + 4 NO_2_^-^ + 2 H^+^ | 8 |
| **I34** | 4 S + 3 NO_3_^-^ + 7 H_2_O → 4 SO_4_^2-^ + 3 NH_4_^+^ + 2 H^+^ | 24 |
| **I35** | 5 S + 6 NO_3_^-^ + 2 H_2_O → 5 SO_4_^2-^ + 3 N_2_ + 4 H^+^ | 30 |
| **I36** | S + 3 NO_3_^-^ + H_2_O → SO_4_^2-^ + 3 NO_2_^-^ + 2 H^+^ | 6 |
| **I37** | 4 FeS_2 Pyrite_ + NO_3_^-^ + 10 H^+^ → 8 S + 4 Fe^2+^ + NH_4_^+^ + 3 H_2_O | 8 |
| **I38** | 4 FeS_2 Pyrite_ + 7 NO_3_^-^ + 6 H^+^ + 11 H_2_O → 8 SO_4_^2-^ + 4 Fe^2+^ + 7 NH_4_^+^ | 56 |
| **I39** | 3 FeS_2 Pyrite_ + NO_3_^-^ + 2 H^+^ + H_2_O → 6 S + Fe_3_O_4 Magnetite_ + NH_4_^+^ | 8 |
| **I40** | 6 FeS_2 Pyrite_ + 11 NO_3_^-^ + 23 H_2_O → 12 SO_4_^2-^ + 2 Fe_3_O_4 Magnetite_ + 11 NH_4_^+^ + 2 H^+^ | 88 |
| **I41** | 5 FeS_2 Pyrite_ + 2 NO_3_^-^ + 12 H^+^ → 10 S + 5 Fe^2+^ + N_2_ + 6 H_2_O | 10 |
| **I42** | 5 FeS_2 Pyrite_ + 14 NO_3_^-^ + 4 H^+^ → 10 SO_4_^2-^ + 5 Fe^2+^ + 7 N_2_ + 2 H_2_O | 70 |
| **I43** | 15 FeS_2 Pyrite_ + 8 NO_3_^-^ + 8 H^+^ → 30 S + 5 Fe_3_O_4 Magnetite_ + 4 N_2_ + 4 H_2_O | 40 |
| **I44** | 15 FeS_2 Pyrite_ + 44 NO_3_^-^ + 8 H_2_O → 30 SO_4_^2-^ + 5 Fe_3_O_4 Magnetite_ + 22 N_2_ + 16 H^+^ | 220 |
| **I45** | FeS_2 Pyrite_ + NO_3_^-^ + 2 H^+^ → 2 S + Fe^2+^ + NO_2_^-^ + H_2_O | 2 |
| **I46** | FeS_2 Pyrite_ + 7 NO_3_^-^ + H_2_O → 2 SO_4_^2-^ + Fe^2+^ + 7 NO_2_^-^ + 2 H^+^ | 14 |
| **I47** | 3 FeS_2 Pyrite_ + 4 NO_3_^-^ → 6 S + Fe_3_O_4 Magnetite_ + 4 NO_2_^-^ | 8 |
| **I48** | 3 FeS_2 Pyrite_ + 22 NO_3_^-^ + 6 H_2_O → 6 SO_4_^2-^ + Fe_3_O_4 Magnetite_ + 22 NO_2_^-^ + 12 H^+^ | 44 |
| **I49** | 12 Fe^2+^ + NO_3_^-^ + 13 H_2_O → 4 Fe_3_O_4 Magnetite_ + NH_4_^+^ + 22 H^+^ | 8 |
| **I50** | 8 Fe^2+^ + NO_3_^-^ + 9 H_2_O → 4 Fe_2_O_3 Hematite_ + NH_4_^+^ + 14 H^+^ | 8 |
| **I51** | 8 Fe^2+^ + NO_3_^-^ + 13 H_2_O → 8 FeOOH _Goethite_ + NH_4_^+^ + 14 H^+^ | 8 |
| **I52** | 8 Fe^2+^ + NO_3_^-^ + 13 H_2_O → 8 FeOOH _Ferrihydrite_ + NH_4_^+^ + 14 H^+^ | 8 |
| **I53** | 15 Fe^2+^ + 2 NO_3_^-^ + 14 H_2_O → 5 Fe_3_O_4 Magnetite_ + N_2_ + 28 H^+^ | 10 |
| **I54** | 10 Fe^2+^ + 2 NO_3_^-^ + 9 H_2_O → 5 Fe_2_O_3 Hematite_ + N_2_ + 18 H^+^ | 10 |
| **I55** | 10 Fe^2+^ + 2 NO_3_^-^ + 14 H_2_O → 10 FeOOH _Goethite_ + N_2_ + 18 H^+^ | 10 |
| **I56** | 10 Fe^2+^ + 2 NO_3_^-^ + 14 H_2_O → 10 FeOOH _Ferrihydrite_ + N_2_ + 18 H^+^ | 10 |
| **I57** | 3 Fe^2+^ + NO_3_^-^ + 3 H_2_O → Fe_3_O_4 Magnetite_ + NO_2_^-^ + 6 H^+^ | 2 |
| **I58** | 2 Fe^2+^ + NO_3_^-^ + 2 H_2_O → Fe_2_O_3 Hematite_ + NO_2_^-^ + 4 H^+^ | 2 |
| **I59** | 2 Fe^2+^ + NO_3_^-^ + 3 H_2_O → 2 FeOOH _Goethite_ + NO_2_^-^ + 4 H^+^ | 2 |
| **I60** | 2 Fe^2+^ + NO_3_^-^ + 3 H_2_O → 2 FeOOH _Ferrihydrite_ + NO_2_^-^ + 4 H^+^ | 2 |
| **I61** | 8 Fe_3_O_4 Magnetite_ + NO_3_^-^ + 2 H^+^ + H_2_O → 12 Fe_2_O_3 Hematite_ + NH_4_^+^ | 8 |
| **I62** | 8 Fe_3_O_4 Magnetite_ + NO_3_^-^ + 2 H^+^ + 13 H_2_O → 24 FeOOH _Goethite_ + NH_4_^+^ | 8 |
| **I63** | 8 Fe_3_O_4 Magnetite_ + NO_3_^-^ + 2 H^+^ + 13 H_2_O → 24 FeOOH _Ferrihydrite_ + NH_4_^+^ | 8 |
| **I64** | 10 Fe_3_O_4 Magnetite_ + 2 NO_3_^-^ + 2 H^+^ → 15 Fe_2_O_3 Hematite_ + N_2_ + H_2_O | 10 |
| **I65** | 10 Fe_3_O_4 Magnetite_ + 2 NO_3_^-^ + 2 H^+^ + 14 H_2_O → 30 FeOOH _Goethite_ + N_2_ | 10 |
| **I66** | 10 Fe_3_O_4 Magnetite_ + 2 NO_3_^-^ + 2 H^+^ + 14 H_2_O → 30 FeOOH _Ferrihydrite_ + N_2_ | 10 |
| **I67** | 2 Fe_3_O_4 Magnetite_ + NO_3_^-^ → 3 Fe_2_O_3 Hematite_ + NO_2_^-^ | 2 |
| **I68** | 2 Fe_3_O_4 Magnetite_ + NO_3_^-^ + 3 H_2_O → 6 FeOOH _Goethite_ + NO_2_^-^ | 2 |
| **I69** | 2 Fe_3_O_4 Magnetite_ + NO_3_^-^ + 3 H_2_O → 6 FeOOH _Ferrihydrite_ + NO_2_^-^ | 2 |
| **I70** | 4 Mn^2+^ + NO_3_^-^ + 5 H_2_O → 4 MnO_2 Pyrolusite_ + NH_4_^+^ + 6 H^+^ | 8 |
| **I71** | 5 Mn^2+^ + 2 NO_3_^-^ + 4 H_2_O → 5 MnO_2 Pyrolusite_ + N_2_ + 8 H^+^ | 10 |
| **I72** | Mn^2+^ + NO_3_^-^ + H_2_O → MnO_2 Pyrolusite_ + NO_2_^-^ + 2 H^+^ | 2 |
| **I73** | 4 H_3_AsO_3_ + NO_3_^-^ + H_2_O → 4 H_2_AsO_4_^-^ + NH_4_^+^ + 2 H^+^ | 8 |
| **I74** | 5 H_3_AsO_3_ + 2 NO_3_^-^ → 5 H_2_AsO_4_^-^ + N_2_ + 3 H^+^ + H_2_O | 10 |
| **I75** | H_3_AsO_3_ + NO_3_^-^ → H_2_AsO_4_^-^ + NO_2_^-^ + H^+^ | 2 |
|  | **Oxidation of Sulfide** |  |
| **J1** | 2 H_2_S + Fe^2+^ → H_2_ + FeS_2 Pyrite_ + 2 H^+^ | 2 |
| **J2** | H_2_S → S + H_2_ | 2 |
| **J3** | FeS_2 Pyrite_ + 2 H^+^ → 2 S + Fe^2+^ + H_2_ | 2 |
| **J4** | 4 H_2_O + 4 FeS_2 Pyrite_ + 6 H^+^ → SO_4_^2-^ + 7 H_2_S + 4 Fe^2+^ | 7 |
|  | **Reduction of Pyrite** |  |
| **K1** | 2 H_2_O + 2 FeS_2 Pyrite_ + 4 H^+^ → O_2_ + 4 H_2_S + 2 Fe^2+^ | 4 |
| **K2** | H_2_ + FeS_2 Pyrite_ + 2H^+^ → Fe^2+^ + 2H_2_S | 2 |
| **K3** | 2 H_2_ + 3 FeS_2 Pyrite_ + 4 H_2_O → Fe_3_O_4 Magnetite_ + 6 H_2_S | 6 |
| **K4** | H_2_ + 2 FeS_2 Pyrite_ + 3 H_2_O → Fe_2_O_3 Hematite_ + 4 H_2_S | 4 |
| **K5** | H_2_ + 2 FeS_2 Pyrite_ + 4 H_2_O → 2 FeOOH _Goethite_ + 4 H_2_S | 4 |
| **K6** | H_2_ + 2 FeS_2 Pyrite_ + 4 H_2_O → 2 FeOOH _Ferrihydrite_ + 4 H_2_S | 4 |
| **K7** | CH_4_ + 3FeS_2 Pyrite_ + 6H^+^ + H_2_O → 6H_2_S + 3Fe^2+^ + CO | 6 |
| **K8** | CH_4_ + 4FeS_2 Pyrite_ + 8H^+^ + 2H_2_O → 8H_2_S + 4Fe^2+^ + CO_2_ | 8 |
| **K9** | CH_4_ + 4FeS_2 Pyrite_ + 7H^+^ + 3H_2_O → 8H_2_S + 4Fe^2+^ + HCO_3_^-^ | 8 |
| **K10** | 2 CH_4_ + 9 FeS_2 Pyrite_ + 14 H_2_O → 3 Fe_3_O_4 Magnetite_ + 2 CO + 18 H_2_S | 18 |
| **K11** | CH_4_ + 6 FeS_2 Pyrite_ + 10 H_2_O → 2 Fe_3_O_4 Magnetite_ + CO_2_ + 12 H_2_S | 12 |
| **K12** | CH_4_ + 6 FeS_2 Pyrite_ + 11 H_2_O → 2 Fe_3_O_4 Magnetite_ + HCO_3_^-^ + 12 H_2_S + H^+^ | 12 |
| **K13** | CH_4_ + 6 FeS_2 Pyrite_ + 10 H_2_O → 3 Fe_2_O_3 Hematite_ + CO + 12 H_2_S | 12 |
| **K14** | CH_4_ + 8 FeS_2 Pyrite_ + 14 H_2_O → 4 Fe_2_O_3 Hematite_ + CO_2_ + 16 H_2_S | 16 |
| **K15** | CH_4_ + 8 FeS_2 Pyrite_ + 15 H_2_O → 4 Fe_2_O_3 Hematite_ + HCO_3_^-^ + 16 H_2_S + H^+^ | 16 |
| **K16** | CH_4_ + 6 FeS_2 Pyrite_ + 13 H_2_O → 6 FeOOH _Goethite_ + CO + 12 H_2_S | 12 |
| **K17** | CH_4_ + 8 FeS_2 Pyrite_ + 18 H_2_O → 8 FeOOH _Goethite_ + CO_2_ + 16 H_2_S | 16 |
| **K18** | CH_4_ + 8 FeS_2 Pyrite_ + 19 H_2_O → 8 FeOOH _Goethite_ + HCO_3_^-^ + 16 H_2_S + H^+^ | 16 |
| **K19** | CH_4_ + 6 FeS_2 Pyrite_ + 13 H_2_O → 6 FeOOH _Ferrihydrite_ + CO + 12 H_2_S | 12 |
| **K20** | CH_4_ + 8 FeS_2 Pyrite_ + 18 H_2_O → 8 FeOOH _Ferrihydrite_ + CO_2_ + 16 H_2_S | 16 |
| **K21** | CH_4_ + 8 FeS_2 Pyrite_ + 19 H_2_O → 8 FeOOH _Ferrihydrite_ + HCO_3_^-^ + 16 H_2_S + H^+^ | 16 |
| **K22** | CO + FeS_2 Pyrite_ + 2 H^+^ + H_2_O → 2 H_2_S + Fe^2+^ + CO_2_ | 2 |
| **K23** | CO + FeS_2 Pyrite_ + H^+^ + 2 H_2_O → 2 H_2_S + Fe^2+^ + HCO_3_^-^ | 2 |
| **K24** | 2 CO + 3 FeS_2 Pyrite_ + 6 H_2_O → Fe_3_O_4 Magnetite_ + 2 CO_2_ + 6 H_2_S | 6 |
| **K25** | 2 CO + 3 FeS_2 Pyrite_ + 8 H_2_O → Fe_3_O_4 Magnetite_ + 2 HCO_3_^-^ + 6 H_2_S + 2 H^+^ | 6 |
| **K26** | CO + 2 FeS_2 Pyrite_ + 4 H_2_O → Fe_2_O_3 Hematite_ + CO_2_ + 4 H_2_S | 4 |
| **K27** | CO + 2 FeS_2 Pyrite_ + 5 H_2_O → Fe_2_O_3 Hematite_ + HCO_3_^-^ + 4 H_2_S + H^+^ | 4 |
| **K28** | CO + 2 FeS_2 Pyrite_ + 5 H_2_O → 2 FeOOH _Goethite_ + CO_2_ + 4 H_2_S | 4 |
| **K29** | CO + 2 FeS_2 Pyrite_ + 6 H_2_O → 2 FeOOH _Goethite_ + HCO_3_^-^ + 4 H_2_S + H^+^ | 4 |
| **K30** | CO + 2 FeS_2 Pyrite_ + 5 H_2_O → 2 FeOOH _Ferrihydrite_ + CO_2_ + 4 H_2_S | 4 |
| **K31** | CO + 2 FeS_2 Pyrite_ + 6 H_2_O → 2 FeOOH _Ferrihydrite_ + HCO_3_^-^ + 4 H_2_S + H^+^ | 4 |
| **K32** | 2 NH_4_^+^ + 3 FeS_2 Pyrite_ + 4 H^+^ → N_2_ + 6 H_2_S + 3 Fe^2+^ | 6 |
| **K33** | NH_4_^+^ + 3 FeS_2 Pyrite_ + 4 H^+^ + 2 H_2_O → NO_2_^-^ + 6 H_2_S + 3 Fe^2+^ | 6 |
| **K34** | NH_4_^+^ + 4 FeS_2 Pyrite_ + 6 H^+^ + 3 H_2_O → NO_3_^-^ + 8 H_2_S + 4 Fe^2+^ | 8 |
| **K35** | 4 NH_4_^+^ + 9 FeS_2 Pyrite_ + 12 H_2_O → 3 Fe_3_O_4 Magnetite_ + 2 N_2_ + 18 H_2_S + 4 H^+^ | 18 |
| **K36** | 2 NH_4_^+^ + 9 FeS_2 Pyrite_ + 16 H_2_O → 3 Fe_3_O_4 Magnetite_ + 2 NO_2_^-^ + 18 H_2_S + 4 H^+^ | 18 |
| **K37** | NH_4_^+^ + 6 FeS_2 Pyrite_ + 11 H_2_O → 2 Fe_3_O_4 Magnetite_ + NO_3_^-^ + 12 H_2_S + 2 H^+^ | 12 |
| **K38** | 2 NH_4_^+^ + 6 FeS_2 Pyrite_ + 9 H_2_O → 3 Fe_2_O_3 Hematite_ + N_2_ + 12 H_2_S + 2 H^+^ | 12 |
| **K39** | NH_4_^+^ + 6 FeS_2 Pyrite_ + 11 H_2_O → 3 Fe_2_O_3 Hematite_ + NO_2_^-^ + 12 H_2_S + 2 H^+^ | 12 |
| **K40** | NH_4_^+^ + 8 FeS_2 Pyrite_ + 15 H_2_O → 4 Fe_2_O_3 Hematite_ + NO_3_^-^ + 16 H_2_S + 2 H^+^ | 16 |
| **K41** | 2 NH_4_^+^ + 6 FeS_2 Pyrite_ + 12 H_2_O → 6 FeOOH _Goethite_ + N_2_ + 12 H_2_S + 2 H^+^ | 12 |
| **K42** | NH_4_^+^ + 6 FeS_2 Pyrite_ + 14 H_2_O → 6 FeOOH _Goethite_ + NO_2_^-^ + 12 H_2_S + 2 H^+^ | 12 |
| **K43** | NH_4_^+^ + 8 FeS_2 Pyrite_ + 19 H_2_O → 8 FeOOH _Goethite_ + NO_3_^-^ + 16 H_2_S + 2 H^+^ | 16 |
| **K44** | 2 NH_4_^+^ + 6 FeS_2 Pyrite_ + 12 H_2_O → 6 FeOOH _Ferrihydrite_ + N_2_ + 12 H_2_S + 2 H^+^ | 12 |
| **K45** | NH_4_^+^ + 6 FeS_2 Pyrite_ + 14 H_2_O → 6 FeOOH _Ferrihydrite_ + NO_2_^-^ + 12 H_2_S + 2 H^+^ | 12 |
| **K46** | NH_4_^+^ + 8 FeS_2 Pyrite_ + 19 H_2_O → 8 FeOOH _Ferrihydrite_ + NO_3_^-^ + 16 H_2_S + 2 H^+^ | 16 |
| **K47** | N_2_ + 3 FeS_2 Pyrite_ + 4 H^+^ + 4 H_2_O → 2 NO_2_^-^ + 6 H_2_S + 3 Fe^2+^ | 6 |
| **K48** | N_2_ + 5 FeS_2 Pyrite_ + 8 H^+^ + 6 H_2_O → 2 NO_3_^-^ + 10 H_2_S + 5 Fe^2+^ | 10 |
| **K49** | 2 N_2_ + 9 FeS_2 Pyrite_ + 20 H_2_O → 3 Fe_3_O_4 Magnetite_ + 4 NO_2_^-^ + 18 H_2_S + 4 H^+^ | 18 |
| **K50** | 2 N_2_ + 15 FeS_2 Pyrite_ + 32 H_2_O → 5 Fe_3_O_4 Magnetite_ + 4 NO_3_^-^ + 30 H_2_S + 4 H^+^ | 30 |
| **K51** | N_2_ + 6 FeS_2 Pyrite_ + 13 H_2_O → 3 Fe_2_O_3 Hematite_ + 2 NO_2_^-^ + 12 H_2_S + 2 H^+^ | 12 |
| **K52** | N_2_ + 10 FeS_2 Pyrite_ + 21 H_2_O → 5 Fe_2_O_3 Hematite_ + 2 NO_3_^-^ + 20 H_2_S + 2 H^+^ | 20 |
| **K53** | N_2_ + 6 FeS_2 Pyrite_ + 16 H_2_O → 6 FeOOH _Goethite_ + 2 NO_2_^-^ + 12 H_2_S + 2 H^+^ | 12 |
| **K54** | N_2_ + 10 FeS_2 Pyrite_ + 26 H_2_O → 10 FeOOH _Goethite_ + 2 NO_3_^-^ + 20 H_2_S + 2 H^+^ | 20 |
| **K55** | N_2_ + 6 FeS_2 Pyrite_ + 16 H_2_O → 6 FeOOH _Ferrihydrite_ + 2 NO_2_^-^ + 12 H_2_S + 2 H^+^ | 12 |
| **K56** | N_2_ + 10 FeS_2 Pyrite_ + 26 H_2_O → 10 FeOOH _Ferrihydrite_ + 2 NO_3_^-^ + 20 H_2_S + 2 H^+^ | 20 |
| **K57** | NO_2_^-^ + FeS_2 Pyrite_ + 2 H^+^ + H_2_O → NO_3_^-^ + 2 H_2_S + Fe^2+^ | 2 |
| **K58** | 2 NO_2_^-^ + 3 FeS_2 Pyrite_ + 6 H_2_O → Fe_3_O_4 Magnetite_ + 2 NO_3_^-^ + 6 H_2_S | 6 |
| **K59** | NO_2_^-^ + 2 FeS_2 Pyrite_ + 4 H_2_O → Fe_2_O_3 Hematite_ + NO_3_^-^ + 4 H_2_S | 4 |
| **K60** | NO_2_^-^ + 2 FeS_2 Pyrite_ + 5 H_2_O → 2 FeOOH _Goethite_ + NO_3_^-^ + 4 H_2_S | 4 |
| **K61** | NO_2_^-^ + 2 FeS_2 Pyrite_ + 5 H_2_O → 2 FeOOH _Ferrihydrite_ + NO_3_^-^ + 4 H_2_S | 4 |
| **K62** | 2 Fe^2+^ + FeS_2 Pyrite_ + 4 H_2_O → Fe_3_O_4 Magnetite_ + 2 H_2_S + 4 H^+^ | 2 |
| **K63** | Fe^2+^ + FeS_2 Pyrite_ + 3 H_2_O → Fe_2_O_3 Hematite_ + 2 H_2_S + 2 H^+^ | 2 |
| **K64** | Fe^2+^ + FeS_2 Pyrite_ + 4 H_2_O → 2 FeOOH _Goethite_ + 2 H_2_S + 2 H^+^ | 2 |
| **K65** | Fe^2+^ + FeS_2 Pyrite_ + 4 H_2_O → 2 FeOOH _Ferrihydrite_ + 2 H_2_S + 2 H^+^ | 2 |
| **K66** | Fe_3_O_4 Magnetite_ + FeS_2 Pyrite_ + 2 H_2_O → 2 Fe_2_O_3 Hematite_ + 2 H_2_S | 2 |
| **K67** | Fe_3_O_4 Magnetite_ + FeS_2 Pyrite_ + 4 H_2_O → 4 FeOOH _Goethite_ + 2 H_2_S | 2 |
| **K68** | Fe_3_O_4 Magnetite_ + FeS_2 Pyrite_ + 4 H_2_O → 4 FeOOH _Ferrihydrite_ + 2 H_2_S | 2 |
| **K69** | Mn^2+^ + FeS_2 Pyrite_ + 2 H_2_O → MnO_2 Pyrolusite_ + Fe^2+^ + 2 H_2_S | 2 |
| **K70** | 2 Mn^2+^ + 3 FeS_2 Pyrite_ + 8 H_2_O → 2 MnO_2_ + Fe_3_O_4 Magnetite_ + 6 H_2_S + 4 H^+^ | 6 |
| **K71** | Mn^2+^ + 2 FeS_2 Pyrite_ + 5 H_2_O → MnO_2 Pyrolusite_ + Fe_2_O_3 Hematite_ + 4 H_2_S + 2 H^+^ | 4 |
| **K72** | Mn^2+^ + 2 FeS_2 Pyrite_ + 6 H_2_O → MnO_2_ + 2 FeOOH _Goethite_ + 4 H_2_S + 2 H^+^ | 4 |
| **K73** | Mn^2+^ + 2 FeS_2 Pyrite_ + 6 H_2_O → MnO_2_ + 2 FeOOH _Ferrihydrite_ + 4 H_2_S + 2 H^+^ | 4 |
| **K74** | H_3_AsO_3_ + FeS_2 Pyrite_ + H^+^ + H_2_O → H_2_AsO_4_^-^ + Fe^2+^ + 2 H_2_S | 2 |
| **K75** | 2 H_3_AsO_3_ + 3 FeS_2 Pyrite_ + 6 H_2_O → 2 H_2_AsO_4_^-^ + Fe_3_O_4 Magnetite_ + 6 H_2_S + 2 H^+^ | 6 |
| **K76** | H_3_AsO_3_ + 2 FeS_2 Pyrite_ + 4 H_2_O → H_2_AsO_4_^-^ + Fe_2_O_3 Hematite_ + 4 H_2_S + H^+^ | 4 |
| **K77** | H_3_AsO_3_ + 2 FeS_2 Pyrite_ + 5 H_2_O → H_2_AsO_4_^-^ + 2 FeOOH _Goethite_ + 4 H_2_S + H^+^ | 4 |
| **K78** | H_3_AsO_3_ + 2 FeS_2 Pyrite_ + 5 H_2_O → H_2_AsO_4_^-^ + 2 FeOOH _Ferrihydrite_ + 4 H_2_S + H^+^ | 4 |
|  | **Reduction and Disproportionation of Elemental Sulfur** |  |
| **L1** | 2 S + 2 H_2_O → 2 H_2_S + O_2_ | 4 |
| **L2** | 4 S + 4 H_2_O → 3 H_2_S + SO_4_^2-^ + 2 H^+^ | 6 |
| **L3** | 2 H_2_O + 4 S + 2 Fe^2+^ → 2 FeS_2 Pyrite_ + O_2_ + 4 H^+^ | 4 |
| **L4** | 4 H_2_O + 7 S + 3 Fe^2+^ → 3 FeS_2 Pyrite_ + SO_4_^2-^ + 8 H^+^ | 6 |
| **L5** | H_2_ + S → H_2_S | 2 |
| **L6** | H_2_ + 2 S + Fe^2+^ → FeS_2 Pyrite_ + 2 H^+^ | 2 |
| **L7** | CH_4_ + 3S + H_2_O → 3H_2_S + CO | 6 |
| **L8** | CH_4_ + 4S + 2H_2_O → 4H_2_S + CO_2_ | 8 |
| **L9** | CH_4_ + 4S + 3H_2_O → 4H_2_S + HCO_3_^-^ + H^+^ | 8 |
| **L10** | CH_4_ + 6 S + 3 Fe^2+^ + H_2_O → 3 FeS_2 Pyrite_ + CO + 6 H^+^ | 6 |
| **L11** | CH_4_ + 8 S + 4 Fe^2+^ + 2 H_2_O → 4 FeS_2 Pyrite_ + CO_2_ + 8 H^+^ | 8 |
| **L12** | CH_4_ + 8 S + 4 Fe^2+^ + 3 H_2_O → 4 FeS_2 Pyrite_ + HCO_3_^-^ + 9 H^+^ | 8 |
| **L13** | CO + S + H_2_O → CO_2_ + H_2_S | 2 |
| **L14** | CO + S + 2 H_2_O → HCO_3_^-^ + H_2_S + H^+^ | 2 |
| **L15** | CO + 2 S + Fe^2+^ + H_2_O → FeS_2 Pyrite_ + CO_2_ + 2 H^+^ | 2 |
| **L16** | CO + 2 S + Fe^2+^ + 2 H_2_O → FeS_2 Pyrite_ + HCO_3_^-^ + 3 H^+^ | 2 |
| **L17** | 2 NH_4_^+^ + 3 S → N_2_ + 3 H_2_S + 2 H^+^ | 6 |
| **L18** | NH_4_^+^ + 3 S + 2 H_2_O → NO_2_^-^ + 3 H_2_S + 2 H^+^ | 6 |
| **L19** | NH_4_^+^ + 4 S + 3 H_2_O → NO_3_^-^ + 4 H_2_S + 2 H^+^ | 8 |
| **L20** | 2 NH_4_^+^ + 6 S + 3 Fe^2+^ → 3 FeS_2 Pyrite_ + N_2_ + 8 H^+^ | 6 |
| **L21** | NH_4_^+^ + 6 S + 3 Fe^2+^ + 2 H_2_O → 3 FeS_2 Pyrite_ + NO_2_^-^ + 8 H^+^ | 6 |
| **L22** | NH_4_^+^ + 8 S + 4 Fe^2+^ + 3 H_2_O → 4 FeS_2 Pyrite_ + NO_3_^-^ + 10 H^+^ | 8 |
| **L23** | N_2_ + 3 S + 4 H_2_O → 2 NO_2_^-^ + 3 H_2_S + 2 H^+^ | 6 |
| **L24** | N_2_ + 5 S + 6 H_2_O → 2 NO_3_^-^ + 5 H_2_S + 2 H^+^ | 10 |
| **L25** | N_2_ + 6 S + 3 Fe^2+^ + 4 H_2_O → 3 FeS_2 Pyrite_ + 2 NO_2_^-^ + 8 H^+^ | 6 |
| **L26** | N_2_ + 10 S + 5 Fe^2+^ + 6 H_2_O → 5 FeS_2 Pyrite_ + 2 NO_3_^-^ + 12 H^+^ | 10 |
| **L27** | NO_2_^-^ + S + H_2_O → NO_3_^-^ + H_2_S | 2 |
| **L28** | NO_2_^-^ + 2 S + Fe^2+^ + H_2_O → FeS_2 Pyrite_ + NO_3_^-^ + 2 H^+^ | 2 |
| **L29** | H_2_S + S + Fe^2+^ → FeS_2 Pyrite_ + 2 H^+^ | 1 |
| **L30** | 3 Fe^2+^ + S + 4 H_2_O → Fe_3_O_4 Magnetite_ + H_2_S + 6 H^+^ | 2 |
| **L31** | 2 Fe^2+^ + S + 3 H_2_O → Fe_2_O_3 Hematite_ + H_2_S + 4 H^+^ | 2 |
| **L32** | 2 Fe^2+^ + S + 4 H_2_O → 2 FeOOH _Goethite_ + H_2_S + 4 H^+^ | 2 |
| **L33** | 2 Fe^2+^ + S + 4 H_2_O → 2 FeOOH _Ferrihydrite_ + H_2_S + 4 H^+^ | 2 |
| **L34** | 4 Fe^2+^ + 2 S + 4 H_2_O → Fe_3_O_4 Magnetite_ + FeS_2 Pyrite_ + 8 H^+^ | 2 |
| **L35** | 3 Fe^2+^ + 2 S + 3 H_2_O → Fe_2_O_3 Hematite_ + FeS_2 Pyrite_ + 6 H^+^ | 2 |
| **L36** | 3 Fe^2+^ + 2 S + 4 H_2_O → 2 FeOOH _Goethite_ + FeS_2 Pyrite_ + 6 H^+^ | 2 |
| **L37** | 3 Fe^2+^ + 2 S + 4 H_2_O → 2 FeOOH _Ferrihydrite_ + FeS_2 Pyrite_ + 6 H^+^ | 2 |
| **L38** | 2 Fe_3_O_4 Magnetite_ + S + H_2_O → 3 Fe_2_O_3 Hematite_ + H_2_S | 2 |
| **L39** | 2 Fe_3_O_4 Magnetite_ + S + 4 H_2_O → 6 FeOOH _Goethite_ + H_2_S | 2 |
| **L40** | 2 Fe_3_O_4 Magnetite_ + S + 4 H_2_O → 6 FeOOH _Ferrihydrite_+ H_2_S | 2 |
| **L41** | 3 Fe_3_O_4 Magnetite_ + 2 S → 4 Fe_2_O_3 Hematite_ + FeS_2 Pyrite_ | 2 |
| **L42** | 3 Fe_3_O_4 Magnetite_ + 2 S + 4 H_2_O → 8 FeOOH _Goethite_ + FeS_2 Pyrite_ | 2 |
| **L43** | 3 Fe_3_O_4 Magnetite_ + 2 S + 4 H_2_O → 8 FeOOH _Ferrihydrite_ + FeS_2 Pyrite_ | 2 |
| **L44** | Mn^2+^ + S + 2 H_2_O → MnO_2 Pyrolusite_ + H_2_S + 2 H^+^ | 2 |
| **L45** | Mn^2+^ + 2 S + Fe^2+^ + 2 H_2_O → MnO_2 Pyrolusite_ + FeS_2 Pyrite_ + 4 H^+^ | 2 |
| **L46** | H_3_AsO_3_ + S + H_2_O → H_2_AsO_4_^-^ + H_2_S + H^+^ | 2 |
| **L47** | H_3_AsO_3_ + 2 S + Fe^2+^ + H_2_O → H_2_AsO_4_^-^ + FeS_2 Pyrite_ + 3 H^+^ | 2 |
|  | **Reduction of SO_4_^2-^** |  |
| **M1** | SO_4_^2-^ + 2 H^+^ → H_2_S + 2 O_2_ | 8 |
| **M2** | 4H_2_ + SO_4_^2-^ + 2H^+^ → H_2_S + 4H_2_O | 8 |
| **M3** | 7 H_2_ + 2 SO_4_^2-^ + Fe^2+^ + 2 H^+^ → FeS_2 Pyrite_ + 8 H_2_O | 14 |
| **M4** | 3H_2_ + SO_4_^2-^ + 2H^+^ → S + 4H_2_O | 6 |
| **M5** | 4CH_4_ + 3SO_4_^2-^ + 6H^+^ → 3H_2_S + 4CO + 8H_2_O | 24 |
| **M6** | CH_4_ + SO_4_^2-^ + 2H^+^ → H_2_S + CO_2_ + 2H_2_O | 8 |
| **M7** | CH_4_ + SO_4_^2-^ + H^+^ → H_2_S + HCO_3_^-^ + H_2_O | 8 |
| **M8** | 7 CH_4_ + 6 SO_4_^2-^ + 3 Fe^2+^ + 6 H^+^ → 7 CO + 3 FeS_2 Pyrite_ + 17 H_2_O | 42 |
| **M9** | 7 CH_4_ + 8 SO_4_^2-^ + 4 Fe^2+^ + 8 H^+^ → 7 CO_2_ + 4 FeS_2 Pyrite_ + 18 H_2_O | 56 |
| **M10** | 7 CH_4_ + 8 SO_4_^2-^ + 4 Fe^2+^ + H^+^ → 7 HCO_3_^-^ + 4 FeS_2 Pyrite_ + 11 H_2_O | 56 |
| **M11** | CH_4_ + SO_4_^2-^ + 2H^+^ → S + CO + 3H_2_O | 6 |
| **M12** | 3CH_4_ + 4SO_4_^2-^ + 8H^+^ → 4S + 3CO_2_ + 10H_2_O | 24 |
| **M13** | 3CH_4_ + 4SO_4_^2-^ + 5H^+^ → 4S + 3HCO_3_^-^ + 7H_2_O | 24 |
| **M14** | 4 CO + SO_4_^2-^ + 2 H^+^ → H_2_S + 4 CO_2_ | 8 |
| **M15** | 4 CO + SO_4_^2-^ + 4 H_2_O → H_2_S + 4 HCO_3_^-^ + 2 H^+^ | 8 |
| **M16** | 7 CO + 2 SO_4_^2-^ + Fe^2+^ + 2 H^+^ → 7 CO_2_ + FeS_2 Pyrite_ + H_2_O | 14 |
| **M17** | 7 CO + 2 SO_4_^2-^ + Fe^2+^ + 6 H_2_O → 7 HCO_3_^-^ + FeS_2 Pyrite_ + 5 H^+^ | 14 |
| **M18** | 3 CO + SO_4_^2-^ + 2 H^+^ → S + 3 CO_2_ + H_2_O | 6 |
| **M19** | 3 CO + SO_4_^2-^ + 2 H_2_O → S + 3 HCO_3_^-^ + H^+^ | 6 |
| **M20** | 8 NH_4_^+^ + 3 SO_4_^2-^ → 4 N_2_ + 3 H_2_S + 2 H^+^ + 12 H_2_O | 24 |
| **M21** | 4 NH_4_^+^ + 3 SO_4_^2-^ → 4 NO_2_^-^ + 3 H_2_S + 2 H^+^ + 4 H_2_O | 24 |
| **M22** | NH_4_^+^ + SO_4_^2-^ → NO_3_^-^ + H_2_S + H_2_O | 8 |
| **M23** | 14 NH_4_^+^ + 6 SO_4_^2-^ + 3 Fe^2+^ → 7 N_2_ + 3 FeS_2 Pyrite_ + 8 H^+^ + 24 H_2_O | 42 |
| **M24** | 7 NH_4_^+^ + 6 SO_4_^2-^ + 3 Fe^2+^ → 7 NO_2_^-^ + 3 FeS_2 Pyrite_ + 8 H^+^ + 10 H_2_O | 42 |
| **M25** | 7 NH_4_^+^ + 8 SO_4_^2-^ + 4 Fe^2+^ → 7 NO_3_^-^ + 4 FeS_2 Pyrite_ + 6 H^+^ + 11 H_2_O | 56 |
| **M26** | 2 NH_4_^+^ + SO_4_^2-^ → N_2_ + S + 4 H_2_O | 6 |
| **M27** | NH_4_^+^ + SO_4_^2-^ → NO_2_^-^ + S + 2 H_2_O | 6 |
| **M28** | 3 NH_4_^+^ + 4 SO_4_^2-^ + 2 H^+^ → 3 NO_3_^-^ + 4 S + 7 H_2_O | 24 |
| **M29** | 4 N_2_ + 3 SO_4_^2-^ + 4 H_2_O → 8 NO_2_^-^ + 3 H_2_S + 2 H^+^ | 24 |
| **M30** | 4 N_2_ + 5 SO_4_^2-^ + 2 H^+^ + 4 H_2_O → 8 NO_3_^-^ + 5 H_2_S | 40 |
| **M31** | 7 N_2_ + 6 SO_4_^2-^ + 3 Fe^2+^ + 4 H_2_O → 14 NO_2_^-^ + 3 FeS_2 Pyrite_ + 8 H^+^ | 42 |
| **M32** | 7 N_2_ + 10 SO_4_^2-^ + 5 Fe^2+^ + 2 H_2_O → 14 NO_3_^-^ + 5 FeS_2 Pyrite_ + 4 H^+^ | 70 |
| **M33** | N_2_ + SO_4_^2-^ → 2 NO_2_^-^ + S | 6 |
| **M34** | 3 N_2_ + 5 SO_4_^2-^ + 4 H^+^ → 6 NO_3_^-^ + 5 S + 2 H_2_O | 30 |
| **M35** | 4 NO_2_^-^ + SO_4_^2-^ + 2 H^+^ → 4 NO_3_^-^ + H_2_S | 8 |
| **M36** | 7 NO_2_^-^ + 2 SO_4_^2-^ + Fe^2+^ + 2 H^+^ → 7 NO_3_^-^ + FeS_2 Pyrite_ + H_2_O | 14 |
| **M37** | 3 NO_2_^-^ + SO_4_^2-^ + 2 H^+^ → 3 NO_3_^-^ + S + H_2_O | 6 |
| **M38** | 7 H_2_S + SO_4_^2-^ + 4 Fe^2+^ → 4 FeS_2 Pyrite_ + 6 H^+^ + 4 H_2_O | 7 |
| **M39** | 3 H_2_S + SO_4_^2-^ + 2 H^+^ → 4 S + 4 H_2_O | 6 |
| **M40** | 3 FeS_2 Pyrite_ + SO_4_^2-^ + 8 H^+^ → 7 S + 3 Fe^2+^ + 4 H_2_O | 6 |
| **M41** | 9 FeS_2 Pyrite_ + 4 SO_4_^2-^ + 8 H^+^ → 22 S + 3 Fe_3_O_4 Magnetite_ + 4 H_2_O | 24 |
| **M42** | 12 Fe^2+^ + SO_4_^2-^ + 12 H_2_O → 4 Fe_3_O_4 Magnetite_ + H_2_S + 22 H^+^ | 8 |
| **M43** | 8 Fe^2+^ + SO_4_^2-^ + 8 H_2_O → 4 Fe_2_O_3 Hematite_ + H_2_S + 14 H^+^ | 8 |
| **M44** | 8 Fe^2+^ + SO_4_^2-^ + 12 H_2_O → 8 FeOOH _Goethite_ + H_2_S + 14 H^+^ | 8 |
| **M45** | 8 Fe^2+^ + SO_4_^2-^ + 12 H_2_O → 8 FeOOH _Ferrihydrite_ + H_2_S + 14 H^+^ | 8 |
| **M46** | 22 Fe^2+^ + 2 SO_4_^2-^ + 20 H_2_O → 7 Fe_3_O_4 Magnetite_ + FeS_2 Pyrite_ + 40 H^+^ | 14 |
| **M47** | 15 Fe^2+^ + 2 SO_4_^2-^ + 13 H_2_O → 7 Fe_2_O_3 Hematite_ + FeS_2 Pyrite_ + 26 H^+^ | 14 |
| **M48** | 15 Fe^2+^ + 2 SO_4_^2-^ + 20 H_2_O → 14 FeOOH _Goethite_ + FeS_2 Pyrite_ + 26 H^+^ | 14 |
| **M49** | 15 Fe^2+^ + 2 SO_4_^2-^ + 20 H_2_O → 14 FeOOH _Ferrihydrite_ + FeS_2 Pyrite_ + 26 H^+^ | 14 |
| **M50** | 9 Fe^2+^ + SO_4_^2-^ + 8 H_2_O → 3 Fe_3_O_4 Magnetite_ + S + 16 H^+^ | 6 |
| **M51** | 6 Fe^2+^ + SO_4_^2-^ + 5 H_2_O → 3 Fe_2_O_3 Hematite_ + S + 10 H^+^ | 6 |
| **M52** | 6 Fe^2+^ + SO_4_^2-^ + 8 H_2_O → 6 FeOOH _Goethite_ + S + 10 H^+^ | 6 |
| **M53** | 6 Fe^2+^ + SO_4_^2-^ + 8 H_2_O → 6 FeOOH _Ferrihydrite_ + S + 10 H^+^ | 6 |
| **M54** | 8 Fe_3_O_4 Magnetite_ + SO_4_^2-^ + 2 H^+^ → 12 Fe_2_O_3 Hematite_ + H_2_S | 8 |
| **M55** | 8 Fe_3_O_4 Magnetite_ + SO_4_^2-^ + 2 H^+^ + 12 H_2_O → 24 FeOOH _Goethite_ + H_2_S | 8 |
| **M56** | 8 Fe_3_O_4 Magnetite_ + SO_4_^2-^ + 2 H^+^ + 12 H_2_O → 24 FeOOH _Ferrihydrite_ + H_2_S | 8 |
| **M57** | 15 Fe_3_O_4 Magnetite_ + 2 SO_4_^2-^ + 4 H^+^ → 22 Fe_2_O_3 Hematite_ + FeS_2 Pyrite_ + 2 H_2_O | 14 |
| **M58** | 15 Fe_3_O_4 Magnetite_ + 2 SO_4_^2-^ + 4 H^+^ + 20 H_2_O → 44 FeOOH _Goethite_ + FeS_2 Pyrite_ | 14 |
| **M59** | 15 Fe_3_O_4 Magnetite_ + 2 SO_4_^2-^ + 4 H^+^ + 20 H_2_O → 44 FeOOH _Ferrihydrite_ + FeS_2 Pyrite_ | 14 |
| **M60** | 6 Fe_3_O_4 Magnetite_ + SO_4_^2-^ + 2 H^+^ → 9 Fe_2_O_3 Hematite_ + S + H_2_O | 6 |
| **M61** | 6 Fe_3_O_4 Magnetite_ + SO_4_^2-^ + 2 H^+^ + 8 H_2_O → 18 FeOOH _Goethite_ + S | 6 |
| **M62** | 6 Fe_3_O_4 Magnetite_ + SO_4_^2-^ + 2 H^+^ + 8 H_2_O → 18 FeOOH _Ferrihydrite_ + S | 6 |
| **M63** | 4 Mn^2+^ + SO_4_^2-^ + 4 H_2_O → 4 MnO_2 Pyrolusite_ + H_2_S + 6 H^+^ | 8 |
| **M64** | 7 Mn^2+^ + 2 SO_4_^2-^ + Fe^2+^ + 6 H_2_O → 7 MnO_2 Pyrolusite_ + FeS_2 Pyrite_ + 12 H^+^ | 14 |
| **M65** | 3 Mn^2+^ + SO_4_^2-^ + 2 H_2_O → 3 MnO_2 Pyrolusite_ + S + 4 H^+^ | 6 |
| **M66** | 4 H_3_AsO_3_ + SO_4_^2-^ → 4 H_2_AsO_4_^-^ + H_2_S + 2 H^+^ | 8 |
| **M67** | 7 H_3_AsO_3_ + 2 SO_4_^2-^ + Fe^2+^ → 7 H_2_AsO_4_^-^ + FeS_2 Pyrite_ + 5 H^+^ + H_2_O | 14 |
| **M68** | 3 H_3_AsO_3_ + SO_4_^2-^ → 3 H_2_AsO_4_^-^ + S + H^+^ + H_2_O | 6 |
|  | **Reduction of Magnetite** |  |
| **N1** | 2 Fe_3_O_4 Magnetite_ + 12 H^+^ → O_2_ + 6 Fe^2+^ + 6 H_2_O | 4 |
| **N2** | H_2_ + Fe_3_O_4 Magnetite_ + 6H^+^ → 3Fe^2+^ + 4H_2_O | 2 |
| **N3** | CH_4_ + 3Fe_3_O_4 Magnetite_ + 18H^+^ → 9Fe^2+^ + CO + 11H_2_O | 6 |
| **N4** | CH_4_ + 4Fe_3_O_4 Magnetite_ + 24H^+^ → 12Fe^2+^ + CO_2_ + 14H_2_O | 8 |
| **N5** | CH_4_ + 4Fe_3_O_4 Magnetite_ + 23H^+^ → 12Fe^2+^ + HCO_3_^-^ + 13H_2_O | 8 |
| **N6** | CO + Fe_3_O_4 Magnetite_ + 6 H^+^ → 3 Fe^2+^ + CO_2_ + 3 H_2_O | 2 |
| **N7** | CO + Fe_3_O_4 Magnetite_ + 5 H^+^ → 3 Fe^2+^ + HCO_3_^-^ + 2 H_2_O | 2 |
| **N8** | 2 NH_4_^+^ + 3 Fe_3_O_4 Magnetite_ + 16 H^+^ → N_2_ + 9 Fe^2+^ + 12 H_2_O | 6 |
| **N9** | NH_4_^+^ + 3 Fe_3_O_4 Magnetite_ + 16 H^+^ → NO_2_^-^ + 9 Fe^2+^ + 10 H_2_O | 6 |
| **N10** | NH_4_^+^ + 4 Fe_3_O_4 Magnetite_ + 22 H^+^ → NO_3_^-^ + 12 Fe^2+^ + 13 H_2_O | 8 |
| **N11** | N_2_ + 3 Fe_3_O_4 Magnetite_ + 16 H^+^ → 2 NO_2_^-^ + 9 Fe^2+^ + 8 H_2_O | 6 |
| **N12** | N_2_ + 5 Fe_3_O_4 Magnetite_ + 28 H^+^ → 2 NO_3_^-^ + 15 Fe^2+^ + 14 H_2_O | 10 |
| **N13** | NO_2_^-^ + Fe_3_O_4 Magnetite_ + 6 H^+^ → NO_3_^-^ + 3 Fe^2+^ + 3 H_2_O | 2 |
| **N14** | 2 H_2_S + Fe_3_O_4_ + 4 H^+^ → FeS_2 pyrite_ + 2 Fe^2+^ + 4 H_2_O | 2 |
| **N15** | H_2_S + Fe_3_O_4 Magnetite_ + 6 H^+^ → S + 3 Fe^2+^ + 4 H_2_O | 2 |
| **N16** | H_2_S + 4 Fe_3_O_4 Magnetite_ + 22 H^+^ → SO_4_^2-^ + 12 Fe^2+^ + 12 H_2_O | 8 |
| **N17** | S + 3 Fe_3_O_4 Magnetite_ + 16 H^+^ → SO_4_^2-^ + 9 Fe^2+^ + 8 H_2_O | 6 |
| **N18** | FeS_2 Pyrite_ + Fe_3_O_4 Magnetite_ + 8 H^+^ → 2 S + 4 Fe^2+^ + 4 H_2_O | 2 |
| **N19** | FeS_2 Pyrite_ + 7 Fe_3_O_4 Magnetite_ + 40 H^+^ → 2 SO_4_^2-^ + 22 Fe^2+^ + 20 H_2_O | 14 |
| **N20** | Mn^2+^ + Fe_3_O_4 Magnetite_ + 4 H^+^ → MnO_2 Pyrolusite_ + 3 Fe^2+^ + 2 H_2_O | 2 |
| **N21** | H_3_AsO_3_ + Fe_3_O_4 Magnetite_ + 5 H^+^ → H_2_AsO_4_^-^ + 3 Fe^2+^ + 3 H_2_O | 2 |
|  | **Reduction of Hematite** |  |
| **O1** | 2 Fe_2_O_3 Hematite_ + 8 H^+^ → O_2_ + 4 Fe^2+^ + 4 H_2_O | 4 |
| **O2** | 6 Fe_2_O_3 Hematite_ → O_2_ + 4 Fe_3_O_4 Magnetite_ | 4 |
| **O3** | H_2_ + Fe_2_O_3 Hematite_ + 4H^+^ → 2Fe^2+^ + 3H_2_O | 2 |
| **O4** | H_2_ + 3Fe_2_O_3 Hematite_ → 2Fe_3_O_4_ _Magnetite_ + H_2_O | 2 |
| **O5** | CH_4_ + 3Fe_2_O_3 Hematite_ + 12H^+^ → 6Fe^2+^ + CO + 8H_2_O | 6 |
| **O6** | CH_4_ + 4Fe_2_O_3 Hematite_ + 16H^+^ → 8Fe^2+^ + CO_2_ + 10H_2_O | 8 |
| **O7** | CH_4_ + 4Fe_2_O_3 Hematite_ + 15H^+^ → 8Fe^2+^ + HCO_3_^-^ + 9H_2_O | 8 |
| **O8** | CH_4_ + 9Fe_2_O_3 Hematite_ → 6Fe_3_O_4 Magnetite_ + CO + 2H_2_O | 6 |
| **O9** | CH_4_ + 12Fe_2_O_3 Hematite_ → 8Fe_3_O_4 Magnetite_ + CO_2_ + 2H_2_O | 8 |
| **O10** | CH_4_ + 12Fe_2_O_3 Hematite_ → 8Fe_3_O_4 Magnetite_ + HCO_3_^-^ + H^+^ + H_2_O | 8 |
| **O11** | CO + Fe_2_O_3 Hematite_ + 4 H^+^ → 2 Fe^2+^ + CO_2_ + 2 H_2_O | 2 |
| **O12** | CO + Fe_2_O_3 Hematite_ + 3 H^+^ → 2 Fe^2+^ + HCO_3_^-^ + H_2_O | 2 |
| **O13** | CO + 3 Fe_2_O_3 Hematite_ → 2 Fe_3_O_4 Magnetite_ + CO_2_ | 2 |
| **O14** | CO + 3 Fe_2_O_3 Hematite_ + H_2_O → 2 Fe_3_O_4 Magnetite_ + HCO_3_^-^ + H^+^ | 2 |
| **O15** | 2 NH_4_^+^ + 3 Fe_2_O_3 Hematite_ + 10 H^+^ → N_2_ + 6 Fe^2+^ + 9 H_2_O | 6 |
| **O16** | NH_4_^+^ + 3 Fe_2_O_3 Hematite_ + 10 H^+^ → NO_2_^-^ + 6 Fe^2+^ + 7 H_2_O | 6 |
| **O17** | NH_4_^+^ + 4 Fe_2_O_3 Hematite_ + 14 H^+^ → NO_3_^-^ + 8 Fe^2+^ + 9 H_2_O | 8 |
| **O18** | 2 NH_4_^+^ + 9 Fe_2_O_3 Hematite_ → N_2_ + 6 Fe_3_O_4 Magnetite_ + 2 H^+^ + 3 H_2_O | 6 |
| **O19** | NH_4_^+^ + 9 Fe_2_O_3 Hematite_ → NO_2_^-^ + 6 Fe_3_O_4 Magnetite_ + 2 H^+^ + H_2_O | 6 |
| **O20** | NH_4_^+^ + 12 Fe_2_O_3 Hematite_ → NO_3_^-^ + 8 Fe_3_O_4 Magnetite_ + 2 H^+^ + H_2_O | 8 |
| **O21** | N_2_ + 3 Fe_2_O_3 Hematite_ + 10 H^+^ → 2 NO_2_^-^ + 6 Fe^2+^ + 5 H_2_O | 6 |
| **O22** | N_2_ + 5 Fe_2_O_3 Hematite_ + 18 H^+^ → 2 NO_3_^-^ + 10 Fe^2+^ + 9 H_2_O | 10 |
| **O23** | N_2_ + 9 Fe_2_O_3 Hematite_ + H_2_O → 2 NO_2_^-^ + 6 Fe_3_O_4 Magnetite_ + 2 H^+^ | 6 |
| **O24** | N_2_ + 15 Fe_2_O_3 Hematite_ + H_2_O → 2 NO_3_^-^ + 10 Fe_3_O_4 Magnetite_ + 2 H^+^ | 10 |
| **O25** | NO_2_^-^ + Fe_2_O_3 Hematite_ + 4 H^+^ → NO_3_^-^ + 2 Fe^2+^ + 2 H_2_O | 2 |
| **O26** | NO_2_^-^ + 3 Fe_2_O_3 Hematite_ → NO_3_^-^ + 2 Fe_3_O_4 Magnetite_ | 2 |
| **O27** | 2 H_2_S + Fe_2_O_3 Hematite_ + 2 H^+^ → FeS_2 Pyrite_ + Fe^2+^ + 3 H_2_O | 2 |
| **O28** | H_2_S + Fe_2_O_3 Hematite_ + 4 H^+^ → S + 2 Fe^2+^ + 3 H_2_O | 2 |
| **O29** | H_2_S + 4 Fe_2_O_3 Hematite_ + 14 H^+^ → SO_4_^2-^ + 8 Fe^2+^ + 8 H_2_O | 8 |
| **O30** | 2 H_2_S + 2 Fe_2_O_3 Hematite_ → FeS_2 Pyrite_ + Fe_3_O_4 Magnetite_ + 2 H_2_O | 2 |
| **O31** | H_2_S + 3 Fe_2_O_3 Hematite_ → S + 2 Fe_3_O_4 Magnetite_ + H_2_O | 2 |
| **O32** | H_2_S + 12 Fe_2_O_3 Hematite_ → SO_4_^2-^ + 8 Fe_3_O_4 Magnetite_ + 2 H^+^ | 8 |
| **O33** | S + 3 Fe_2_O_3 Hematite_ + 10 H^+^ → SO_4_^2-^ + 6 Fe^2+^ + 5 H_2_O | 6 |
| **O34** | S + 9 Fe_2_O_3 Hematite_ + H_2_O → SO_4_^2-^ + 6 Fe_3_O_4 Magnetite_ + 2 H^+^ | 6 |
| **O35** | FeS_2 Pyrite_ + Fe_2_O_3 Hematite_ + 6 H^+^ → 2 S + 3 Fe^2+^ + 3 H_2_O | 2 |
| **O36** | FeS_2 Pyrite_ + 7 Fe_2_O_3 Hematite_ + 26 H^+^ → 2 SO_4_^2-^ + 15 Fe^2+^ + 13 H_2_O | 14 |
| **O37** | FeS_2 Pyrite_ + 22 Fe_2_O_3 Hematite_ + 2 H_2_O → 2 SO_4_^2-^ + 15 Fe_3_O_4 Magnetite_ + 4 H^+^ | 14 |
| **O38** | FeS_2 Pyrite_ + 4 Fe_2_O_3 Hematite_ → 2 S + 3 Fe_3_O_4 Magnetite_ | 2 |
| **O39** | Mn^2+^ + Fe_2_O_3 Hematite_ + 2 H^+^ → MnO_2 Pyrolusite_ + 2 Fe^2+^ + H_2_O | 2 |
| **O40** | Mn^2+^ + 3 Fe_2_O_3 Hematite_ + H_2_O → MnO_2 Pyrolusite_ + 2 Fe_3_O_4 Magnetite_ + 2 H^+^ | 2 |
| **O41** | H_3_AsO_3_ + Fe_2_O_3 Hematite_ + 3 H^+^ → H_2_AsO_4_^-^ + 2 Fe^2+^ + 2 H_2_O | 2 |
| **O42** | H_3_AsO_3_ + 3 Fe_2_O_3 Hematite_ → H_2_AsO_4_^-^ + 2 Fe_3_O_4 Magnetite_ + H^+^ | 2 |
|  | **Reduction of Goethite** |  |
| **P1** | 4 FeOOH _Goethite_ + 8 H^+^ → O_2_ + 4 Fe^2+^ + 6 H_2_O | 4 |
| **P2** | 12 FeOOH _Goethite_ → O_2_ + 4 Fe_3_O_4 Magnetite_ + 6 H_2_O | 4 |
| **P3** | H_2_ + 2FeOOH _Goethite_ + 4H^+^ → 2Fe^2+^ + 4H_2_O | 2 |
| **P4** | H_2_ + 6FeOOH _Goethite_ → 2Fe_3_O_4_ _Magnetite_ + 4H_2_O | 2 |
| **P5** | CH_4_ + 6FeOOH _Goethite_ + 12H^+^ → 6Fe^2+^ + CO + 11H_2_O | 6 |
| **P6** | CH_4_ + 8FeOOH _Goethite_ + 16H^+^ → 8Fe^2+^ + CO_2_ + 14H_2_O | 8 |
| **P7** | CH_4_ + 8FeOOH _Goethite_ + 15H^+^ → 8Fe^2+^ + HCO_3_^-^ + 13H_2_O | 8 |
| **P8** | CH_4_ + 18FeOOH _Goethite_ → 6Fe_3_O_4 Magnetite_ + CO + 11H_2_O | 6 |
| **P9** | CH_4_ + 24FeOOH _Goethite_ → 8Fe_3_O_4 Magnetite_ + CO_2_ + 14H_2_O | 8 |
| **P10** | CH_4_ + 24FeOOH _Goethite_ → 8Fe_3_O_4 Magnetite_ + HCO_3_^-^ + H^+^ + 13H_2_O | 8 |
| **P11** | CO + 2 FeOOH _Goethite_ + 4 H^+^ → 2 Fe^2+^ + CO_2_ + 3 H_2_O | 2 |
| **P12** | CO + 2 FeOOH _Goethite_ + 3 H^+^ → 2 Fe^2+^ + HCO_3_^-^ + 2 H_2_O | 2 |
| **P13** | CO + 6 FeOOH _Goethite_ → 2 Fe_3_O_4 Magnetite_ + CO_2_ + 3 H_2_O | 2 |
| **P14** | CO + 6 FeOOH _Goethite_ → 2 Fe_3_O_4 Magnetite_ + HCO_3_^-^ + H^+^ + 2 H_2_O | 2 |
| **P15** | 2 NH_4_^+^ + 6 FeOOH _Goethite_ + 10 H^+^ → N_2_ + 6 Fe^2+^ + 12 H_2_O | 6 |
| **P16** | NH_4_^+^ + 6 FeOOH _Goethite_ + 10 H^+^ → NO_2_^-^ + 6 Fe^2+^ + 10 H_2_O | 6 |
| **P17** | NH_4_^+^ + 8 FeOOH _Goethite_ + 14 H^+^ → NO_3_^-^ + 8 Fe^2+^ + 13 H_2_O | 8 |
| **P18** | 2 NH_4_^+^ + 18 FeOOH _Goethite_ → N_2_ + 6 Fe_3_O_4 Magnetite_ + 2 H^+^ + 12 H_2_O | 6 |
| **P19** | NH_4_^+^ + 18 FeOOH _Goethite_ → NO_2_^-^ + 6 Fe_3_O_4 Magnetite_ + 2 H^+^ + 10 H_2_O | 6 |
| **P20** | NH_4_^+^ + 24 FeOOH _Goethite_ → NO_3_^-^ + 8 Fe_3_O_4 Magnetite_ + 2 H^+^ + 13 H_2_O | 8 |
| **P21** | N_2_ + 6 FeOOH _Goethite_ + 10 H^+^ → 2 NO_2_^-^ + 6 Fe^2+^ + 8 H_2_O | 6 |
| **P22** | N_2_ + 10 FeOOH _Goethite_ + 18 H^+^ → 2 NO_3_^-^ + 10 Fe^2+^ + 14 H_2_O | 10 |
| **P23** | N_2_ + 18 FeOOH _Goethite_ → 2 NO_2_^-^ + 6 Fe_3_O_4 Magnetite_ + 2 H^+^ + 8 H_2_O | 6 |
| **P24** | N_2_ + 30 FeOOH _Goethite_ → 2 NO_3_^-^ + 10 Fe_3_O_4 Magnetite_ + 2 H^+^ + 14 H_2_O | 10 |
| **P25** | NO_2_^-^ + 2 FeOOH _Goethite_ + 4 H^+^ → NO_3_^-^ + 2 Fe^2+^ + 3 H_2_O | 2 |
| **P26** | NO_2_^-^ + 6 FeOOH _Goethite_ → NO_3_^-^ + 2 Fe_3_O_4 Magnetite_ + 3 H_2_O | 2 |
| **P27** | 2 H_2_S + 2 FeOOH _Goethite_ + 2 H^+^ → FeS_2 Pyrite_ + Fe^2+^ + 4 H_2_O | 2 |
| **P28** | H_2_S + 2 FeOOH _Goethite_ + 4 H^+^ → S + 2 Fe^2+^ + 4 H_2_O | 2 |
| **P29** | H_2_S + 8 FeOOH _Goethite_ + 14 H^+^ → SO_4_^2-^ + 8 Fe^2+^ + 12 H_2_O | 8 |
| **P30** | 2 H_2_S + 4 FeOOH _Goethite_ → FeS_2 Pyrite_ + Fe_3_O_4 Magnetite_ + 4 H_2_O | 2 |
| **P31** | H_2_S + 6 FeOOH _Goethite_ → S + 2 Fe_3_O_4 Magnetite_ + 4 H_2_O | 2 |
| **P32** | H_2_S + 24 FeOOH _Goethite_ → SO_4_^2-^ + 8 Fe_3_O_4 Magnetite_ + 2 H^+^ + 12 H_2_O | 8 |
| **P33** | S + 6 FeOOH _Goethite_ + 10 H^+^ → SO_4_^2-^ + 6 Fe^2+^ + 8 H_2_O | 6 |
| **P34** | S + 18 FeOOH _Goethite_ → SO_4_^2-^ + 6 Fe_3_O_4 Magnetite_ + 2 H^+^ + 8 H_2_O | 6 |
| **P35** | FeS_2 Pyrite_ + 2 FeOOH _Goethite_ + 6 H^+^ → 2 S + 3 Fe^2+^ + 4 H_2_O | 2 |
| **P36** | FeS_2 Pyrite_ + 14 FeOOH _Goethite_ + 26 H^+^ → 2 SO_4_^2-^ + 15 Fe^2+^ + 20 H_2_O | 14 |
| **P37** | FeS_2 Pyrite_ + 8 FeOOH _Goethite_ → 2 S + 3 Fe_3_O_4_ + 4 H_2_O | 2 |
| **P38** | FeS_2 Pyrite_ + 44 FeOOH _Goethite_ → 2 SO_4_^2-^ + 15 Fe_3_O_4 Magnetite_ + 4 H^+^ + 20 H_2_O | 14 |
| **P39** | Fe^2+^ + 2 FeOOH _Goethite_ → Fe_3_O_4 Magnetite_ + 2 H^+^ | 0 |
| **P40** | Mn^2+^ + 2 FeOOH _Goethite_ + 2 H^+^ → MnO_2 Pyrolusite_ + 2 Fe^2+^ + 2 H_2_O | 2 |
| **P41** | Mn^2+^ + 6 FeOOH _Goethite_ → MnO_2 Pyrolusite_ + 2 Fe_3_O_4 Magnetite_ + 2 H^+^ + 2 H_2_O | 2 |
| **P42** | H_3_AsO_3_ + 2 FeOOH _Goethite_ + 3 H^+^ → H_2_AsO_4_^-^ + 2 Fe^2+^ + 3 H_2_O | 2 |
| **P43** | H_3_AsO_3_ + 6 FeOOH _Goethite_ → H_2_AsO_4_^-^ + 2 Fe_3_O_4 Magnetite_ + H^+^ + 3 H_2_O | 2 |
|  | **Reduction of Ferrihydrite** |  |
| **Q1** | 4 FeOOH _Ferrihydrite_ + 8 H^+^ → O_2_ + 4 Fe^2+^ + 6 H_2_O | 4 |
| **Q2** | 12 FeOOH _Ferrihydrite_ → O_2_ + 4 Fe_3_O_4 Magnetite_ + 6 H_2_O | 4 |
| **Q3** | H_2_ + 2FeOOH _Ferrihydrite_ + 4H^+^ → 2Fe^2+^ + 4H_2_O | 2 |
| **Q4** | H_2_ + 6FeOOH _Ferrihydrite_ → 2Fe_3_O_4_ _Magnetite_ + 4H_2_O | 2 |
| **Q5** | CH_4_ + 6FeOOH _Ferrihydrite_ + 12H^+^ → 6Fe^2+^ + CO + 11H_2_O | 6 |
| **Q6** | CH_4_ + 8FeOOH _Ferrihydrite_ + 16H^+^ → 8Fe^2+^ + CO_2_ + 14H_2_O | 8 |
| **Q7** | CH_4_ + 8FeOOH _Ferrihydrite_ + 15H^+^ → 8Fe^2+^ + HCO_3_^-^ + 13H_2_O | 8 |
| **Q8** | CH_4_ + 18FeOOH _Ferrihydrite_ → 6Fe_3_O_4 Magnetite_ + CO + 11H_2_O | 6 |
| **Q9** | CH_4_ + 24FeOOH _Ferrihydrite_ → 8Fe_3_O_4 Magnetite_ + CO_2_ + 14H_2_O | 8 |
| **Q10** | CH_4_ + 24FeOOH _Ferrihydrite_ → 8Fe_3_O_4 Magnetite_ + HCO_3_^-^ + H^+^ + 13H_2_O | 8 |
| **Q11** | CO + 2 FeOOH _Ferrihydrite_ + 4 H^+^ → 2 Fe^2+^ + CO_2_ + 3 H_2_O | 2 |
| **Q12** | CO + 2 FeOOH _Ferrihydrite_ + 3 H^+^ → 2 Fe^2+^ + HCO_3_^-^ + 2 H_2_O | 2 |
| **Q13** | CO + 6 FeOOH _Ferrihydrite_ → 2 Fe_3_O_4 Magnetite_ + CO_2_ + 3 H_2_O | 2 |
| **Q14** | CO + 6 FeOOH _Ferrihydrite_ → 2 Fe_3_O_4 Magnetite_ + HCO_3_^-^ + H^+^ + 2 H_2_O | 2 |
| **Q15** | 2 NH_4_^+^ + 6 FeOOH _Ferrihydrite_ + 10 H^+^ → N_2_ + 6 Fe^2+^ + 12 H_2_O | 6 |
| **Q16** | NH_4_^+^ + 6 FeOOH _Ferrihydrite_ + 10 H^+^ → NO_2_^-^ + 6 Fe^2+^ + 10 H_2_O | 6 |
| **Q17** | NH_4_^+^ + 8 FeOOH _Ferrihydrite_ + 14 H^+^ → NO_3_^-^ + 8 Fe^2+^ + 13 H_2_O | 8 |
| **Q18** | 2 NH_4_^+^ + 18 FeOOH _Ferrihydrite_ → N_2_ + 6 Fe_3_O_4 Magnetite_ + 2 H^+^ + 12 H_2_O | 6 |
| **Q19** | NH_4_^+^ + 18 FeOOH _Ferrihydrite_ → NO_2_^-^ + 6 Fe_3_O_4 Magnetite_ + 2 H^+^ + 10 H_2_O | 6 |
| **Q20** | NH_4_^+^ + 24 FeOOH _Ferrihydrite_ → NO_3_^-^ + 8 Fe_3_O_4 Magnetite_ + 2 H^+^ + 13 H_2_O | 8 |
| **Q21** | N_2_ + 6 FeOOH _Ferrihydrite_ + 10 H^+^ → 2 NO_2_^-^ + 6 Fe^2+^ + 8 H_2_O | 6 |
| **Q22** | N_2_ + 10 FeOOH _Ferrihydrite_ + 18 H^+^ → 2 NO_3_^-^ + 10 Fe^2+^ + 14 H_2_O | 10 |
| **Q23** | N_2_ + 18 FeOOH _Ferrihydrite_ → 2 NO_2_^-^ + 6 Fe_3_O_4 Magnetite_ + 2 H^+^ + 8 H_2_O | 6 |
| **Q24** | N_2_ + 30 FeOOH _Ferrihydrite_ → 2 NO_3_^-^ + 10 Fe_3_O_4 Magnetite_ + 2 H^+^ + 14 H_2_O | 10 |
| **Q25** | NO_2_^-^ + 2 FeOOH _Ferrihydrite_ + 4 H^+^ → NO_3_^-^ + 2 Fe^2+^ + 3 H_2_O | 2 |
| **Q26** | NO_2_^-^ + 6 FeOOH _Ferrihydrite_ → NO_3_^-^ + 2 Fe_3_O_4 Magnetite_ + 3 H_2_O | 2 |
| **Q27** | 2 H_2_S + 2 FeOOH _Ferrihydrite_ + 2 H^+^ → FeS_2 Pyrite_ + Fe^2+^ + 4 H_2_O | 2 |
| **Q28** | H_2_S + 2 FeOOH _Ferrihydrite_ + 4 H^+^ → S + 2 Fe^2+^ + 4 H_2_O | 2 |
| **Q29** | H_2_S + 8 FeOOH _Ferrihydrite_ + 14 H^+^ → SO_4_^2-^ + 8 Fe^2+^ + 12 H_2_O | 8 |
| **Q30** | 2 H_2_S + 4 FeOOH _Ferrihydrite_ → FeS_2 Pyrite_ + Fe_3_O_4 Magnetite_ + 4 H_2_O | 2 |
| **Q31** | H_2_S + 6 FeOOH _Ferrihydrite_ → S + 2 Fe_3_O_4 Magnetite_ + 4 H_2_O | 2 |
| **Q32** | H_2_S + 24 FeOOH _Ferrihydrite_ → SO_4_^2-^ + 8 Fe_3_O_4 Magnetite_ + 2 H^+^ + 12 H_2_O | 8 |
| **Q33** | S + 6 FeOOH _Ferrihydrite_ + 10 H^+^ → SO_4_^2-^ + 6 Fe^2+^ + 8 H_2_O | 6 |
| **Q34** | S + 18 FeOOH _Ferrihydrite_ → SO_4_^2-^ + 6 Fe_3_O_4 Magnetite_ + 2 H^+^ + 8 H_2_O | 6 |
| **Q35** | FeS_2 Pyrite_ + 2 FeOOH _Ferrihydrite_ + 6 H^+^ → 2 S + 3 Fe^2+^ + 4 H_2_O | 2 |
| **Q36** | FeS_2 Pyrite_ + 14 FeOOH _Ferrihydrite_ + 26 H^+^ → 2 SO_4_^2-^ + 15 Fe^2+^ + 20 H_2_O | 14 |
| **Q37** | FeS_2 Pyrite_ + 8 FeOOH _Ferrihydrite_ → 2 S + 3 Fe_3_O_4_ + 4 H_2_O | 2 |
| **Q38** | FeS_2 Pyrite_ + 44 FeOOH _Ferrihydrite_ → 2 SO_4_^2-^ + 15 Fe_3_O_4 Magnetite_ + 4 H^+^ + 20 H_2_O | 14 |
| **Q39** | Fe^2+^ + 2 FeOOH _Ferrihydrite_ → Fe_3_O_4 Magnetite_ + 2 H^+^ | 0 |
| **Q40** | Mn^2+^ + 2 FeOOH _Ferrihydrite_ + 2 H^+^ → MnO_2 Pyrolusite_ + 2 Fe^2+^ + 2 H_2_O | 2 |
| **Q41** | Mn^2+^ + 6 FeOOH _Ferrihydrite_ → MnO_2 Pyrolusite_ + 2 Fe_3_O_4 Magnetite_ + 2 H^+^ + 2 H_2_O | 2 |
| **Q42** | H_3_AsO_3_ + 2 FeOOH _Ferrihydrite_ + 3 H^+^ → H_2_AsO_4_^-^ + 2 Fe^2+^ + 3 H_2_O | 2 |
| **Q43** | H_3_AsO_3_ + 6 FeOOH _Ferrihydrite_ → H_2_AsO_4_^-^ + 2 Fe_3_O_4 Magnetite_ + H^+^ + 3 H_2_O | 2 |
|  | **Reduction of Pyrolusite** |  |
| **R1** | H_2_ + MnO_2 Pyrolusite_ + 2H^+^ → Mn^2+^ + 2H_2_O | 2 |
| **R2** | CH_4_ + 3MnO_2 Pyrolusite_ + 6H^+^ → 3Mn^2+^ + CO + 5H_2_O | 6 |
| **R3** | CH_4_ + 4MnO_2 Pyrolusite_ + 8H^+^ → 4Mn^2+^ + CO_2_ + 6H_2_O | 8 |
| **R4** | CH_4_ + 4MnO_2 Pyrolusite_ + 7H^+^ → 4Mn^2+^ + HCO_3_^-^ + 5H_2_O | 8 |
| **R5** | CO + MnO_2 Pyrolusite_ + 2 H^+^ → Mn^2+^ + CO_2_ + H_2_O | 2 |
| **R6** | CO + MnO_2 Pyrolusite_ + H^+^ → Mn^2+^ + HCO_3_^-^ | 2 |
| **R7** | 2 NH_4_^+^ + 3 MnO_2 Pyrolusite_ + 4 H^+^ → N_2_ + 3 Mn^2+^ + 6 H_2_O | 6 |
| **R8** | NH_4_^+^ + 3 MnO_2 Pyrolusite_ + 4 H^+^ → NO_2_^-^ + 3 Mn^2+^ + 4 H_2_O | 6 |
| **R9** | NH_4_^+^ + 4 MnO_2 Pyrolusite_ + 6 H^+^ → NO_3_^-^ + 4 Mn^2+^ + 5 H_2_O | 8 |
| **R10** | N_2_ + 3 MnO_2 Pyrolusite_ + 4 H^+^ → 2 NO_2_^-^ + 3 Mn^2+^ + 2 H_2_O | 6 |
| **R11** | N_2_ + 5 MnO_2 Pyrolusite_ + 8 H^+^ → 2 NO_3_^-^ + 5 Mn^2+^ + 4 H_2_O | 10 |
| **R12** | NO_2_^-^ + MnO_2 Pyrolusite_ + 2 H^+^ → NO_3_^-^ + Mn^2+^ + H_2_O | 2 |
| **R13** | 2 H_2_S + MnO_2 Pyrolusite_ + Fe^2+^ → FeS_2 Pyrite_ + Mn^2+^ + 2 H_2_O | 2 |
| **R14** | H_2_S + MnO_2 Pyrolusite_ + 2 H^+^ → S + Mn^2+^ + 2 H_2_O | 2 |
| **R15** | H_2_S + 4 MnO_2 Pyrolusite_ + 6 H^+^ → SO_4_^2-^ + 4 Mn^2+^ + 4 H_2_O | 8 |
| **R16** | S + 3 MnO_2 Pyrolusite_ + 4 H^+^ → SO_4_^2-^ + 3 Mn^2+^ + 2 H_2_O | 6 |
| **R17** | FeS_2 Pyrite_ + MnO_2 Pyrolusite_ + 4 H^+^ → 2 S + Fe^2+^ + Mn^2+^ + 2 H_2_O | 2 |
| **R18** | FeS_2 Pyrite_ + 7 MnO_2 Pyrolusite_ + 12 H^+^ → 2 SO_4_^2-^ + Fe^2+^ + 7 Mn^2+^ + 6 H_2_O | 14 |
| **R19** | 3 FeS_2 Pyrite_ + 4 MnO_2 Pyrolusite_ + 8 H^+^ → 6 S + Fe_3_O_4 Magnetite_ + 4 Mn^2+^ + 4 H_2_O | 6 |
| **R20** | 3 FeS_2 Pyrite_ + 22 MnO_2 Pyrolusite_ + 32 H^+^ → 6 SO_4_^2-^ + Fe_3_O_4 Magnetite_ + 22 Mn^2+^ + 16 H_2_O | 44 |
| **R21** | 3 Fe^2+^ + MnO_2 Pyrolusite_ + 2 H_2_O → Fe_3_O_4 Magnetite_ + Mn^2+^ + 4 H^+^ | 2 |
| **R22** | 2 Fe^2+^ + MnO_2 Pyrolusite_ + H_2_O → Fe_2_O_3 Hematite_ + Mn^2+^ + 2 H^+^ | 2 |
| **R23** | 2 Fe^2+^ + MnO_2 Pyrolusite_ + 2 H_2_O → 2 FeOOH _Goethite_ + Mn^2+^ + 2 H^+^ | 2 |
| **R24** | 2 Fe^2+^ + MnO_2 Pyrolusite_ + 2 H_2_O → 2 FeOOH _Ferrihydrite_ + Mn^2+^ + 2 H^+^ | 2 |
| **R25** | 2 Fe_3_O_4 Magnetite_ + MnO_2 Pyrolusite_ + 2 H^+^ → 3 Fe_2_O_3 Hematite_ + Mn^2+^ + H_2_O | 2 |
| **R26** | 2 Fe_3_O_4 Magnetite_ + MnO_2 Pyrolusite_ + 2 H^+^ + 2 H_2_O → 6 FeOOH _Goethite_ + Mn^2+^ | 2 |
| **R27** | 2 Fe_3_O_4 Magnetite_ + MnO_2 Pyrolusite_ + 2 H^+^ + 2 H_2_O → 6 FeOOH _Ferrihydrite_ + Mn^2+^ | 2 |
| **R28** | H_3_AsO_3_ + MnO_2 Pyrolusite_ + H^+^ → H_2_AsO_4_^-^ + Mn^2+^ + H_2_O | 2 |
|  | **Reduction of Arsenate** |  |
| **S1** | H_2_ + H_2_AsO_4_^-^ + H^+^ → H_3_AsO_3_ + H_2_O | 2 |
| **S2** | CH_4_ + 3H_2_AsO_4_^-^ + 3H^+^ → 3H_3_AsO_3_ + CO + 2H_2_O | 6 |
| **S3** | CH_4_ + 4H_2_AsO_4_^-^ + 4H^+^ → 4H_3_AsO_3_ + CO_2_ + 2H_2_O | 8 |
| **S4** | CH_4_ + 4H_2_AsO_4_^-^ + 3H^+^ → 4H_3_AsO_3_ + HCO_3_^-^ + H_2_O | 8 |
| **S5** | CO + H_2_AsO_4_^-^ + H^+^ → H_3_AsO_3_ + CO_2_ | 2 |
| **S6** | CO + H_2_AsO_4_^-^ + H_2_O → H_3_AsO_3_ + HCO_3_^-^ | 2 |
| **S7** | 2 NH_4_^+^ + 3 H_2_AsO_4_^-^ + H^+^ → N_2_ + 3 H_3_AsO_3_ + 3 H_2_O | 6 |
| **S8** | NH_4_^+^ + 3 H_2_AsO_4_^-^ + H^+^ → NO_2_^-^ + 3 H_3_AsO_3_ + H_2_O | 6 |
| **S9** | NH_4_^+^ + 4 H_2_AsO_4_^-^ + 2 H^+^ → NO_3_^-^ + 4 H_3_AsO_3_ + H_2_O | 8 |
| **S10** | N_2_ + 3 H_2_AsO_4_^-^ + H^+^ + H_2_O → 2 NO_2_^-^ + 3 H_3_AsO_3_ | 6 |
| **S11** | N_2_ + 5 H_2_AsO_4_^-^ + 3 H^+^ + H_2_O → 2 NO_3_^-^ + 5 H_3_AsO_3_ | 10 |
| **S12** | NO_2_^-^ + H_2_AsO_4_^-^ + H^+^ → NO_3_^-^ + H_3_AsO_3_ | 2 |
| **S13** | 2 H_2_S + H_2_AsO_4_^-^ + Fe^2+^ → FeS_2 Pyrite_ + H_3_AsO_3_ + H^+^ + H_2_O | 2 |
| **S14** | H_2_S + H_2_AsO_4_^-^ + H^+^ → S + H_3_AsO_3_ + H_2_O | 2 |
| **S15** | H_2_S + 4 H_2_AsO_4_^-^ + 2 H^+^ → SO_4_^2-^ + 4 H_3_AsO_3_ | 8 |
| **S16** | S + 3 H_2_AsO_4_^-^ + H^+^ + H_2_O → SO_4_^2-^ + 3 H_3_AsO_3_ | 6 |
| **S17** | FeS_2 Pyrite_ + H_2_AsO_4_^-^ + 3 H^+^ → 2 S + Fe^2+^ + H_3_AsO_3_ + H_2_O | 2 |
| **S18** | FeS_2 Pyrite_ + 7 H_2_AsO_4_^-^ + 5 H^+^ + H_2_O → 2 SO_4_^2-^ + Fe^2+^ + 7 H_3_AsO_3_ | 14 |
| **S19** | 3 FeS_2 Pyrite_ + 4 H_2_AsO_4_^-^ + 4 H^+^ → 6 S + Fe_3_O_4 Magnetite_ + 4 H_3_AsO_3_ | 8 |
| **S20** | 3 FeS_2 Pyrite_ + 22 H_2_AsO_4_^-^ + 10 H^+^ + 6 H_2_O → 6 SO_4_^2-^ + Fe_3_O_4 Magnetite_ + 22 H_3_AsO_3_ | 44 |
| **S21** | 3 Fe^2+^ + H_2_AsO_4_^-^ + 3 H_2_O → Fe_3_O_4 Magnetite_ + H_3_AsO_3_ + 5 H^+^ | 2 |
| **S22** | 2 Fe^2+^ + H_2_AsO_4_^-^ + 2 H_2_O → Fe_2_O_3 Hematite_ + H_3_AsO_3_ + 3 H^+^ | 2 |
| **S23** | 2 Fe^2+^ + H_2_AsO_4_^-^ + 3 H_2_O → 2 FeOOH _Goethite_ + H_3_AsO_3_ + 3 H^+^ | 2 |
| **S24** | 2 Fe^2+^ + H_2_AsO_4_^-^ + 3 H_2_O → 2 FeOOH _Ferrihydrite_ + H_3_AsO_3_ + 3 H^+^ | 2 |
| **S25** | 2 Fe_3_O_4 Magnetite_ + H_2_AsO_4_^-^ + H^+^ → 3 Fe_2_O_3 Hematite_ + H_3_AsO_3_ | 2 |
| **S26** | 2 Fe_3_O_4 Magnetite_ + H_2_AsO_4_^-^ + H^+^ + 3 H_2_O → 6 FeOOH _Goethite_ + H_3_AsO_3_ | 2 |
| **S27** | 2 Fe_3_O_4 Magnetite_ + H_2_AsO_4_^-^ + H^+^ + 3 H_2_O → 6 FeOOH _Ferrihydrite_ + H_3_AsO_3_ | 2 |
| **S28** | Mn^2+^ + H_2_AsO_4_^-^ + H_2_O → MnO_2 Pyrolusite_ + H_3_AsO_3_ + H^+^ | 2 |
