# Supplementary material for: Bioenergetic characterization of a shallow-sea hydrothermal vent system: Milos Island, Greece
Source: PLoS One. 2020 Jun 5;15(6):e0234175. doi: 10.1371/journal.pone.0234175 (PMC7274409; doi:10.1371/journal.pone.0234175)
Supplement: S2 Table — (DOCX) [file pone.0234175.s002.docx]

Table S2. Order of reactions shown in Fig. 2 from top (1) to bottom (379)

| # | Rxn |  | # | Rxn |  | # | Rxn |  | # | Rxn |  | # | Rxn |  | # | Rxn |  | # | Rxn |  |
| --- | --- | --- | --- | --- | --- | --- | --- | --- | --- | --- | --- | --- | --- | --- | --- | --- | --- | --- | --- | --- |
| 1 | H13 |  | 57 | I22 |  | 113 | I67 |  | 169 | N2 |  | 225 | O30 |  | 281 | Q12 |  | 337 | P9 |  |
| 2 | H14 |  | 58 | B19 |  | 114 | H7 |  | 170 | A4 |  | 226 | P30 |  | 282 | L38 |  | 338 | K26 |  |
| 3 | B5 |  | 59 | R16 |  | 115 | H39 |  | 171 | L14 |  | 227 | H47 |  | 283 | D17 |  | 339 | D42 |  |
| 4 | I18 |  | 60 | I42 |  | 116 | I9 |  | 172 | O27 |  | 228 | S3 |  | 284 | R27 |  | 340 | E3 |  |
| 5 | B6 |  | 61 | R15 |  | 117 | H3 |  | 173 | P27 |  | 229 | H26 |  | 285 | B28 |  | 341 | L7 |  |
| 6 | R5 |  | 62 | N6 |  | 118 | I4 |  | 174 | N17 |  | 230 | I39 |  | 286 | M9 |  | 342 | I23 |  |
| 7 | H19 |  | 63 | R7 |  | 119 | O12 |  | 175 | M13 |  | 231 | S26 |  | 287 | L47 |  | 343 | O10 |  |
| 8 | I19 |  | 64 | I44 |  | 120 | I6 |  | 176 | B12 |  | 232 | S25 |  | 288 | C3 |  | 344 | M26 |  |
| 9 | R6 |  | 65 | I29 |  | 121 | Q37 |  | 177 | M17 |  | 233 | I47 |  | 289 | L9 |  | 345 | P10 |  |
| 10 | Q13 |  | 66 | H28 |  | 122 | I43 |  | 178 | B9 |  | 234 | O33 |  | 290 | N18 |  | 346 | G13 |  |
| 11 | H9 |  | 67 | I10 |  | 123 | B21 |  | 179 | O13 |  | 235 | Q39 |  | 291 | L5 |  | 347 | M8 |  |
| 12 | H44 |  | 68 | R18 |  | 124 | Q6 |  | 180 | B8 |  | 236 | C10 |  | 292 | G4 |  | 348 | D3 |  |
| 13 | H43 |  | 69 | Q9 |  | 125 | H22 |  | 181 | P13 |  | 237 | P33 |  | 293 | M10 |  | 349 | R11 |  |
| 14 | H10 |  | 70 | H30 |  | 126 | R17 |  | 182 | I56 |  | 238 | S4 |  | 294 | G21 |  | 350 | G9 |  |
| 15 | H4 |  | 71 | Q30 |  | 127 | I34 |  | 183 | H48 |  | 239 | O29 |  | 295 | M6 |  | 351 | S23 |  |
| 16 | B13 |  | 72 | R20 |  | 128 | N14 |  | 184 | G7 |  | 240 | L10 |  | 296 | M3 |  | 352 | Q25 |  |
| 17 | H23 |  | 73 | R14 |  | 129 | Q43 |  | 185 | I73 |  | 241 | P29 |  | 297 | M55 |  | 353 | O35 |  |
| 18 | Q14 |  | 74 | R2 |  | 130 | I36 |  | 186 | N16 |  | 242 | S1 |  | 298 | G20 |  | 354 | S22 |  |
| 19 | H21 |  | 75 | Q10 |  | 131 | H18 |  | 187 | M15 |  | 243 | O15 |  | 299 | B11 |  | 355 | Q17 |  |
| 20 | H15 |  | 76 | Q4 |  | 132 | I27 |  | 188 | I75 |  | 244 | P15 |  | 300 | M54 |  | 356 | Q16 |  |
| 21 | I28 |  | 77 | B29 |  | 133 | Q7 |  | 189 | N8 |  | 245 | M38 |  | 301 | C13 |  | 357 | P11 |  |
| 22 | H20 |  | 78 | Q34 |  | 134 | S6 |  | 190 | H45 |  | 246 | B27 |  | 302 | Q36 |  | 358 | M39 |  |
| 23 | H8 |  | 79 | N7 |  | 135 | Q3 |  | 191 | S13 |  | 247 | Q11 |  | 303 | M12 |  | 359 | K25 |  |
| 24 | R13 |  | 80 | L15 |  | 136 | I16 |  | 192 | M19 |  | 248 | O36 |  | 304 | M61 |  | 360 | K28 |  |
| 25 | B3 |  | 81 | H16 |  | 137 | H25 |  | 193 | L11 |  | 249 | N21 |  | 305 | L2 |  | 361 | K26 |  |
| 26 | B26 |  | 82 | I25 |  | 138 | I38 |  | 194 | R24 |  | 250 | G8 |  | 306 | G5 |  | 362 | L34 |  |
| 27 | H11 |  | 83 | Q32 |  | 139 | I15 |  | 195 | L12 |  | 251 | Q26 |  | 307 | H35 |  | 363 | S17 |  |
| 28 | B25 |  | 84 | I31 |  | 140 | I46 |  | 196 | N15 |  | 252 | S16 |  | 308 | I52 |  | 364 | K8 |  |
| 29 | I20 |  | 85 | Q18 |  | 141 | L13 |  | 197 | H34 |  | 253 | Q20 |  | 309 | M60 |  | 365 | K29 |  |
| 30 | B4 |  | 86 | H36 |  | 142 | Q33 |  | 198 | H33 |  | 254 | K23 |  | 310 | G2 |  | 366 | K27 |  |
| 31 | B1 |  | 87 | Q38 |  | 143 | I40 |  | 199 | L6 |  | 255 | Q19 |  | 311 | M7 |  | 367 | K9 |  |
| 32 | B16 |  | 88 | B23 |  | 144 | H17 |  | 200 | N19 |  | 256 | O28 |  | 312 | L36 |  | 368 | K2 |  |
| 33 | I11 |  | 89 | R19 |  | 145 | K44 |  | 201 | I51 |  | 257 | P28 |  | 313 | C12 |  | 369 | P12 |  |
| 34 | I65 |  | 90 | B22 |  | 146 | I26 |  | 202 | I50 |  | 258 | H32 |  | 314 | O41 |  | 370 | N13 |  |
| 35 | B20 |  | 91 | I74 |  | 147 | I53 |  | 203 | A5 |  | 259 | I49 |  | 315 | L35 |  | 371 | N10 |  |
| 36 | B15 |  | 92 | O11 |  | 148 | I48 |  | 204 | N3 |  | 260 | S15 |  | 316 | I60 |  | 372 | N9 |  |
| 37 | I64 |  | 93 | Q31 |  | 149 | L42 |  | 205 | I59 |  | 261 | O5 |  | 317 | P42 |  | 373 | L32 |  |
| 38 | B7 |  | 94 | Q8 |  | 150 | H5 |  | 206 | Q42 |  | 262 | P5 |  | 318 | M2 |  | 374 | L31 |  |
| 39 | I12 |  | 95 | Q27 |  | 151 | I32 |  | 207 | I58 |  | 263 | I57 |  | 319 | C4 |  | 375 | E20 |  |
| 40 | I5 |  | 96 | B17 |  | 152 | I7 |  | 208 | O6 |  | 264 | S7 |  | 320 | L17 |  | 376 | S21 |  |
| 41 | H49 |  | 97 | R28 |  | 153 | L41 |  | 209 | O14 |  | 265 | M20 |  | 321 | M4 |  | 377 | K30 |  |
| 42 | H12 |  | 98 | I55 |  | 154 | Q29 |  | 210 | P6 |  | 266 | H1 |  | 322 | I71 |  | 378 | L37 |  |
| 43 | I17 |  | 99 | I54 |  | 155 | I13 |  | 211 | R12 |  | 267 | S18 |  | 323 | A2 |  | 379 | L30 |  |
| 44 | B18 |  | 100 | I41 |  | 156 | M16 |  | 212 | P14 |  | 268 | I66 |  | 324 | A21 |  |  |  |  |
| 45 | R3 |  | 101 | L16 |  | 157 | Q15 |  | 213 | R9 |  | 269 | J1 |  | 325 | C15 |  |  |  |  |
| 46 | R26 |  | 102 | S5 |  | 158 | G6 |  | 214 | L4 |  | 270 | E17 |  | 326 | M23 |  |  |  |  |
| 47 | I21 |  | 103 | H6 |  | 159 | R21 |  | 215 | R8 |  | 271 | S20 |  | 327 | E42 |  |  |  |  |
| 48 | R25 |  | 104 | I8 |  | 160 | M14 |  | 216 | O7 |  | 272 | S14 |  | 328 | A20 |  |  |  |  |
| 49 | B14 |  | 105 | H41 |  | 161 | L29 |  | 217 | P7 |  | 273 | C2 |  | 329 | G11 |  |  |  |  |
| 50 | R4 |  | 106 | I62 |  | 162 | I33 |  | 218 | K22 |  | 274 | C23 |  | 330 | E41 |  |  |  |  |
| 51 | B2 |  | 107 | I14 |  | 163 | B24 |  | 219 | H24 |  | 275 | M58 |  | 331 | D41 |  |  |  |  |
| 52 | R1 |  | 108 | H40 |  | 164 | N4 |  | 220 | O3 |  | 276 | S2 |  | 332 | C17 |  |  |  |  |
| 53 | I35 |  | 109 | I61 |  | 165 | M18 |  | 221 | P3 |  | 277 | L8 |  | 333 | O9 |  |  |  |  |
| 54 | I30 |  | 110 | R23 |  | 166 | Q28 |  | 222 | I37 |  | 278 | C22 |  | 334 | C11 |  |  |  |  |
| 55 | H38 |  | 111 | I68 |  | 167 | Q5 |  | 223 | L20 |  | 279 | L39 |  | 335 | G10 |  |  |  |  |
| 56 | H37 |  | 112 | R22 |  | 168 | N5 |  | 224 | I45 |  | 280 | M57 |  | 336 | A3 |  |  |  |  |
